# Stress-associated developmental reprogramming in moss protonemata by synthetic activation of the common symbiosis pathway

**DOI:** 10.1016/j.isci.2022.103754

**Published:** 2022-01-11

**Authors:** Thomas J. Kleist, Anthony Bortolazzo, Zachary P. Keyser, Adele M. Perera, Thomas B. Irving, Muthusubramanian Venkateshwaran, Fatiha Atanjaoui, Ren-Jie Tang, Junko Maeda, Heather N. Cartwright, Michael L. Christianson, Peggy G. Lemaux, Sheng Luan, Jean-Michel Ané

**Affiliations:** 1 Department of Plant & Microbial Biology, University of California-Berkeley, Berkeley, CA 94720, USA; 2 Department of Plant Biology, Carnegie Institute for Science, Stanford, CA 94305, USA; 3 Laboratory of Genetics, University of Wisconsin-Madison, Madison, WI 53706, USA; 4 Department of Bacteriology, University of Wisconsin-Madison, Madison, WI 53706, USA; 5 Department of Agronomy, University of Wisconsin-Madison, Madison, WI 53706, USA; 6 Institute for Molecular Physiology, Department of Biology, Heinrich Heine University, Düsseldorf 40225, Germany; 7 School of Agriculture, University of Wisconsin-Platteville, Platteville, WI 53818, USA

**Keywords:** Mycology, Developmental biology, Plant biology

## Abstract

Symbioses between angiosperms and rhizobia or arbuscular mycorrhizal fungi are controlled through a conserved signaling pathway. Microbe-derived, chitin-based elicitors activate plant cell surface receptors and trigger nuclear calcium oscillations, which are decoded by a calcium/calmodulin-dependent protein kinase (CCaMK) and its target transcription factor interacting protein of DMI3 (IPD3). Genes encoding CCaMK and IPD3 have been lost in multiple non-mycorrhizal plant lineages yet retained among non-mycorrhizal mosses. Here, we demonstrated that the moss *Physcomitrium* is equipped with a *bona fide* CCaMK that can functionally complement a *Medicago* loss-of-function mutant. Conservation of regulatory phosphosites allowed us to generate predicted hyperactive forms of *Physcomitrium* CCaMK and IPD3. Overexpression of synthetically activated CCaMK or IPD3 in *Physcomitrium* led to abscisic acid (ABA) accumulation and ectopic development of brood cells, which are asexual propagules that facilitate escape from local abiotic stresses. We therefore propose a functional role for *Physcomitrium* CCaMK-IPD3 in stress-associated developmental reprogramming

## Highlights

• Conservation of a ‘symbiotic’ calcium-decoding module in a non-mycorrhizal moss
• Restoration of symbiosis in legume mutants by complementation with moss homologs
• Stress-associated developmental reprogramming by synthetic activation in moss

## Graphical abstract

**Figure:**
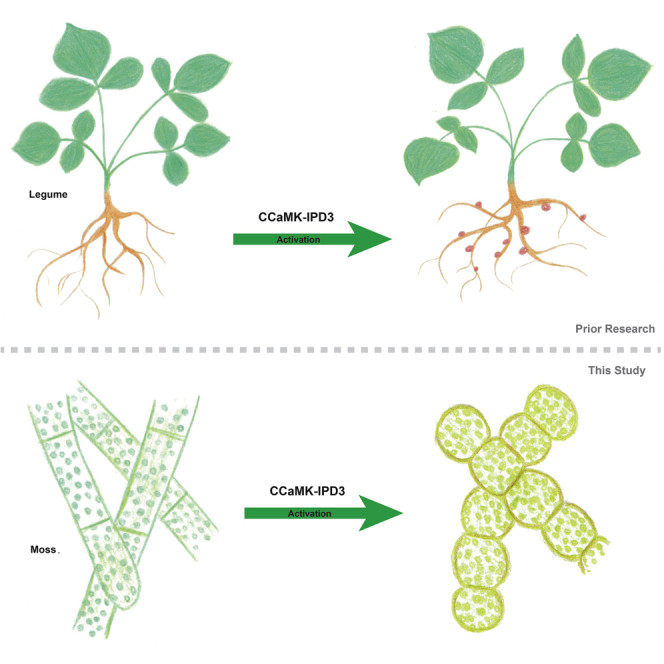

## Introduction

During their early evolution, plants faced numerous challenges in the shift from freshwater to terrestrial environments. These problems included decreased water availability, the sparsity of nutrients, and increased UV radiation levels. The shared ancestor of extant land plants evolved several strategies to surmount these stressors. For example, arbuscular mycorrhizal fungi (AMF) and AMF-like interactions with fungal mutualists likely aided early land plants in acquiring water and nutrients (Bonfante and Genre, 2008; Pirozynski and Malloch, 1975; Read et al., 2000). Arbuscular mycorrhizae are controlled infections of plant roots by fungi of the Glomeromycotina (Parniske, 2008; Spatafora et al., 2016). The establishment of intracellular arbuscules within cortical root cells enables the fungus to provide the plant host with greater access to resources such as phosphate, nitrogen, potassium, and water in exchange for host photosynthates (Garcia et al., 2017; Parniske, 2008; Smith and Read, 2010).

Endomycorrhizal, AMF-like interactions occur in early-diverging plant lineages, including some liverworts. Moreover, fossil samples provide evidence for ancient AMF-like associations. Endophytic structures with a striking similarity to arbuscules are present in the Early Devonian fossil record of the Rhynie chert (Remy et al., 1994; Strullu-Derrien et al., 2014, 2015). Fossilized fungal spores with similar morphology to extant AMF have been found in the Ordovician (Redecker et al., 2000).The broad phylogenetic distribution of AMF and AMF-like host lineages among land plants and the available fossil evidence point toward establishing plant-fungal symbioses early in land plant evolution (Wang and Qiu, 2006). Mosses are one of the earliest diverging and most diverse lineages of extant land plants. Whereas numerous pathogenic, saprotrophic, and commensal fungal interactions have been described in mosses (Davey and Currah, 2006), no convincing evidence has been published to date for *bona fide* mutualistic interactions among mosses and AMF with the possible exception of *Takakia*, which is distantly related to other extant mosses (Newton et al., 2000; Liu et al., 2019). A few reports describing observations of AMF within moss samples (e.g., Rabatin, 1980; Carleton and Read, 1991) were likely due to misinterpretation of fungal growth present in senescent or dead plant tissues.

Perception and accommodation of AMF are achieved through a conserved signal transduction pathway in plants, often referred to to as the “common symbiosis pathway” (Delaux et al., 2013; Oldroyd, 2013). AM fungi exude chitooligosaccharides and lipo-chitooligosaccharides (LCOs) into the rhizosphere. LysM-receptor-like kinases (RLKs) at the plasma membrane of plant cells directly bind to these chitin-derived molecules and are required for colonization (Maillet et al., 2011; Brohammer et al., 2012; Fliegmann et al., 2013; Buendia et al., 2015; Sun et al., 2015). Activation of RLKs upon ligand binding ultimately leads to repetitive oscillations of calcium concentrations, often referred to as “calcium spikes”, in plant nuclei (Ané et al., 2004; Capoen et al., 2011). These oscillatory calcium signals are decoded by a calcium/calmodulin-dependent protein kinase (CCaMK, also known as DOESN’T MAKE INFECTIONS three or DMI3 in *Medicago truncatula*) (Levy et al., 2004; Miller et al., 2013). Activated CCaMK phosphorylates the transcription factor interacting protein of DMI3 (IPD3, also known as CYCLOPS in *Lotus japonicus*) at two serine residues required for infection (Chen et al., 2008; Horváth et al., 2011; Messinese et al., 2007; Singh et al., 2014). CCaMK and IPD3 initiate transcriptional cascades required for developmental reprogramming and AMF colonization by working in concert with numerous GRAS transcription factors (Gobbato et al., 2012; Xue et al., 2015). Endosymbiotic interactions with nitrogen-fixing rhizobial bacteria arose roughly 90 million years ago in select land plant lineages, most notably the “nitrogen-fixing clade” of the Rosids (Doyle, 2011). Similar to AMF, rhizobia communicate with their host-plant in the rhizosphere through LCO exudates. Indeed, many of the core symbiosis signaling components are also required for rhizobial colonization of legume roots and nitrogen fixation (Venkateshwaran et al., 2013).

**Figure 1:**
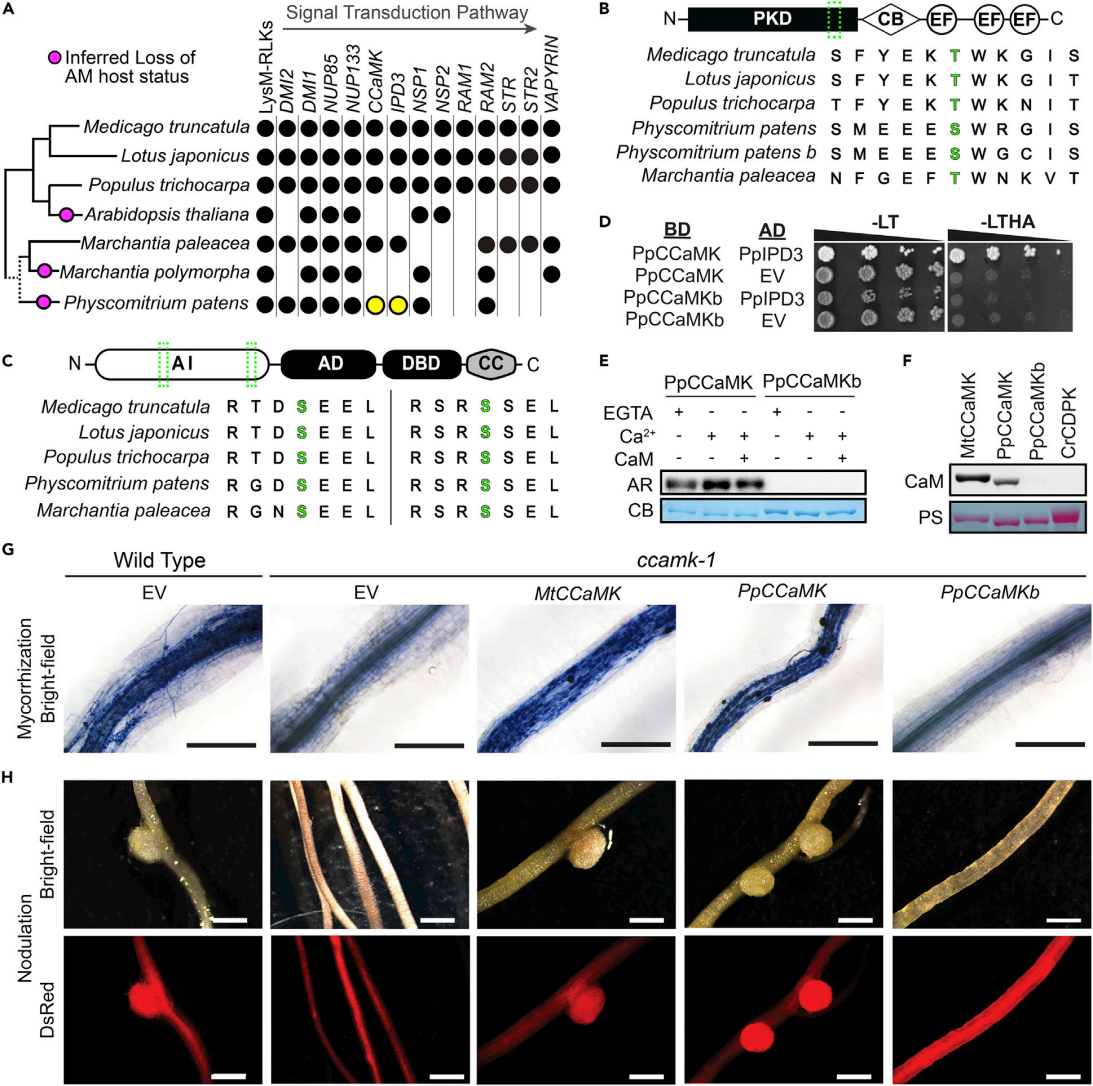
Functional conservation of CCaMK and IPD3 in Physcomitrium (A) Species cladogram (left) showing the presence or absence of symbiotic signaling genes in corresponding lineages (right). Physcomitrium *CCaMK* and *IPD3* are highlighted in yellow. See Table S1 for details. (B) Domain architecture diagram of CCaMK and multiple sequence alignment of the region (green) surrounding the regulatory autophosphorylation site (green). CB: CaM-binding domain, PKD: protein-kinase domain, EF: Calcium-binding EF-hand. (C) Domain architecture diagram of IPD3 and multiple sequence alignments of regions (green) surrounding two regulatory phospho-sites (green) that are necessary and sufficient for activation of LjIPD3/CYCLOPS. AI: autoinhibitory domain, AD: activation domain, DBD: DNA-binding domain, CC: coiled-coil domain. (D) PpCCaMK interacted with PpIPD3 in yeast two-hybrid assay, whereas PpCCaMKb or empty vector (EV) controls did not. The left panel shows growth on control (-LT) media; the right panel shows growth on the test (-LTHA) media to screen for physical interactions. AD: activating-domain, BD: DNA-binding-domain. (E) Kinase assays using purified recombinant proteins showed that PpCCaMK but not PpCCaMKb exhibited kinase activity and that kinase activity is responsive to calcium (Ca^2+^) and CaM. AR: autoradiogram, CB: Coomassie Brilliant Blue stain. (F) Calmodulin-binding assays show that PpCCaMK or positive control (MtCCaMK) binds calmodulin, whereas PpCCaMKb or negative control from *Chlamydomonas reinhardtii* (CrCDPK) does not. PS: Ponceau S staining. (G) *PpCCaMK* rescues the arbuscular mycorrhizal defects of the *Medicago truncatula ccamk-1*mutant. Roots transformed with *MtCCaMK* or *PpCCaMK* developed intracellular hyphae, arbuscules, and vesicles after inoculation with *Rhizophagus irregularis*. (H) *PpCCaMK* rescues the nodulation defects of the *Medicago truncatula ccamk-1*mutant. Roots transformed with *MtCCaMK* or *PpCCaMK* developed root nodules after inoculation with *Sinorhizobium meliloti*. Black scale bars = 500 μm. White scale bars = 2 mm

Nearly all AMF-host plants that have been studied possess the full complement of this core signaling pathway, from angiosperms to liverworts (Delaux et al., 2015; Wang et al., 2010). In several instances, plant lineages that have lost the ability to host AMF have also lost several symbiosis pathway genes. This correlation is exemplified by the Brassicaceae in which many species, including the model plant *Arabidopsis thaliana* (Figure 1A, Table S1), are unable to host AMF and have concomitantly lost many of the core common signaling components (Delaux et al., 2014; Garcia et al., 2015). The retention of symbiosis signaling genes in non-mycorrhizal mosses, including the model organism *Physcomitrium patens* (Physcomitrium, formerly *Physcomitrella patens*)*,* provides a striking counter-example (Delaux et al., 2015; Rensing et al., 2020; Wang et al., 2010). Given that mosses have retained the vertically inherited symbiosis signaling pathway yet cannot establish AMF or AMF-like interactions, we pursued an investigation of the biochemical properties and physiological function(s) of these proteins in mosses using Physcomitrium as a model.

*CCaMK* and *IPD3* are two of the genes whose presence or absence most strongly correlates with AMF host compatibility or incompatibility, respectively, in studied plant lineages (Delaux et al., 2014; Garcia et al., 2015; Wang et al., 2010). Moreover, genetic studies in legumes have elucidated mutational strategies to produce gain-of-function variants of either of these two proteins that can auto-activate root nodule development in the absence of symbionts or symbiont-derived signals, a phenomenon termed spontaneous nodulation. Expression of a constitutively active CCaMK, lacking the C-terminal autoinhibitory domain, in *Medicago truncatula* (Medicago) or *Lotus japonicus* (Lotus) is sufficient to cause the development of root nodules in the absence of rhizobia or rhizobial exudates (Gleason et al., 2006; Tirichine et al., 2006). Spontaneous nodule development can also be achieved by substituting an aspartate for a threonine residue in the kinase auto-activation loop of Medicago CCaMK. In Lotus, nuclear-localized and constitutively active CCaMK induced the partial development of the pre-penetration apparatus, a structure that facilitates hyphal entry of AMF into host roots (Genre et al., 2008; Takeda et al., 2012). A pair of phosphomimetic substitutions in the IPD3 ortholog, CYCLOPS, in Lotus is likewise sufficient to induce spontaneous development of root nodules (Singh et al., 2014). These legume gain-of-function mutants revealed the pivotal role of the CCaMK-IPD3 module in this signaling pathway. We hypothesized that similar molecular genetic manipulations in Physcomitrium might lead to phenotypes that could provide clues to the possible biological relevance of these genes in mosses.

In this study, we investigated the evolutionary conservation, biochemical activities, and physiological function(s) of the two CCaMK and sole IPD3 homologs present in the Physcomitrium genome. We cloned the coding sequence of each homolog from cDNA. We used yeast two-hybrid and biochemical assays to demonstrate that one of two CCaMKs and the sole IPD3 homolog from Physcomitrium have retained many of the biochemical properties required for CCaMK and IPD3 functionality in angiosperms. We further demonstrated that the Physcomitrium CCaMK, which shared biochemical properties with angiosperm CCaMKs, could restore both nodulation and mycorrhization when expressed in a Medicago *ccamk-1* mutant background defective for both symbioses. Additionally, Physcomitrium IPD3 is capable of partially restoring nodulation defects in Medicago *ipd3-1* mutants. Transgenic expression of modified forms of CCaMK and IPD3 predicted to show constitutive activation in Physcomitrium (but not the unmodified forms driven by the same promoter) promoted ectopic development of brood cells, a well-characterized developmental program of mosses in response to drought or osmotic stress. Brood cell development was accompanied by changes in abiotic stress-responsive *LEA* gene transcript levels and elevated amounts of abscisic acid (ABA). Whereas activation of PpCCaMK or PpIPD3 promoted brood cell development, genetic deletion of either the *CCaMK* or *IPD3* loci from Physcomitrium was insufficient to block brood cell development in response to osmotic stress treatment, suggesting other pathways exist for activation of brood cell development. Unexpectedly, we observed prominent nuclear calcium oscillations in Physcomitrium protonemata in the absence of any experimental treatment (i.e., spontaneous). This is in stark contrast to published data on root cells of legume species (Ehrhardt et al., 1996; Chabaud et al., 2011). We therefore propose that CCaMK-IPD3 activation by nuclear calcium levels may be more complex than in studied model legumes, as changes in oscillation frequency or amplitude may trigger activation in moss protonemata. Our results collectively indicate that the Physcomitrium CCaMK-IPD3 signaling module has retained many of the biochemical properties that typify these components in symbiont host plants and that the CCaMK-IPD3 module regulates ABA levels and associated developmental reprogramming to promote escape from adverse environmental conditions.

## Results

### Conservation of the CCaMK-IPD3 signaling module in Physcomitrium

Homologs of CCaMK encoded in the *Physcomitrium patens* genome sequence version 3.3 were identified by BLAST (Altschul et al., 1990) of the predicted proteome using Medicago CCaMK/DMI3 (MtCCaMK, Phytozome: Medtr8g043970) and Lotus CCaMK (LjCCaMK, Lotus Base: Lj3g3v1739280) as queries. The top five hits were used for reciprocal BLAST against the predicted Medicago or Lotus proteomes (Figures S1A, S1B). The two most significant BLAST hits in Physcomitrium, Phytozome: *Pp3c21_15330V3* (E-value = 0, hereafter PpCCaMK) and Phytozome: *Pp3c19_20580V3* (E = 6 × 10^−171^, hereafter PpCCaMKb), each returned MtCCaMK or LjCCaMK as the top reciprocal BLAST hit with highly significant E-values (E ≤ 6 × 10^−176^). The top reciprocal BLAST hits for other loci were identified as calcium-dependent protein kinases (CDPKs), which lack the distinctive CaM-binding site found in CCaMKs. Thus, it appears that up to two loci in the Physcomitrium genome may encode functional CCaMKs. Full-length coding sequences (CDS) were cloned from each locus to validate inferred gene models. Amino acid sequences were aligned using MUSCLE (Edgar, 2004), and the resulting sequence alignment corroborated that the protein kinase domain, auto-activation loop, predicted CaM-binding site, and three calcium-binding EF-hand domains were each conserved in candidate PpCCaMKs (Figure S1C). Closer inspection of the auto-activation loop, which is required for MtCCaMK function, revealed that PpCCaMK and PpCCaMKb each have a serine residue at the position orthologous to the auto-phosphorylated threonine residue (T271) in MtCCaMK (Figure 1B), which suggests that PpCCaMK and/or PpCCaMKb may likewise be subject to regulatory autophosphorylation.

To identify potential IPD3 homologs encoded in the Physcomitrella genome, we employed a similar strategy. The protein sequences of Medicago IPD3 (MtIPD3, Phytozome: Medtr5g026850) or Lotus IPD3/CYCLOPS (LjIPD3, Lotus Base: Lj2g3v1549600) were used as queries; and, in each search, only a single locus (Phytozome: *Pp3c23_22500V3*) yielded a significant E-value (E = 1 × 10^−21^ or E = 1 × 10^−22^, respectively). Results of reciprocal BLAST searches of the predicted proteomes of Medicago or Lotus corroborated that Phytozome: *Pp3c23_22500V3* is the sole Physcomitrium locus encoding an IPD3 homolog (E < 1 × 10^−16^, Figures S2A, S2B). Cloning and sequencing of Physcomitrium *IPD3* from total gametophyte RNA revealed that the most abundant transcript spliceform was annotated as Phytozome: *Pp3c23_22500V3.2* (or Phytozome: *Pp3c23_22500V3.5*, which differ only in untranslated regions). The inferred full-length PpIPD3 protein sequence was aligned to MtIPD3, LjIPD3, and a recently identified paralog of MtIPD3 from Medicago named IPD3L (Jin et al., 2018). The multiple sequence alignment revealed that each of the functionally important regions described for Medicago or Lotus IPD3, including CCaMK-targeted phospho-motifs and a C-terminal coiled-coil domain, are also present in PpIPD3 (Figure S2C). In particular, two sequence motifs surrounding CCaMK-targeted phosphosites necessary and sufficient for activation of LjIPD3 are strongly conserved in PpIPD3 (Figure 1C), suggesting that CCaMK-mediated phosphoregulation of IPD3 may be conserved in Physcomitrium.

If identified CCaMK and IPD3 homologs constitute a functional signaling module in Physcomitrium, the respective genes should be co-expressed in the same cell types. To determine and compare the relative expression patterns of *PpCCaMK*, *PpCCaMKb*, and *PpIPD3*, we mined their expression profiles from two Physcomitrium transcriptome atlas studies (Frank and Scanlon, 2015; Ortiz-Ramírez et al., 2016).Data from both studies confirmed that *PpCCaMK*, *PpCCaMKb*, and *PpIPD3*show overlapping expression patterns. Each is expressed in protonema, which we had expected based on our ability to clone each CDS from protonemal cDNA. Moreover, *PpCCaMK* showed greater transcript abundance than *PpCCaMKb* in all tested tissues (Figure S3). Physical interaction between CCaMK and IPD3 has been demonstrated in multiple legume models (Messinese et al., 2007; Yano et al., 2008). We tested whether PpCCaMK or PpCCaMKb could interact with PpIPD3 in yeast two-hybrid (Y2H) assays. PpIPD3 was fused to the GAL4 split-transcription factor activation domain (AD) and tested in pairwise combination with PpCCaMK or PpCCaMKb fused to the GAL4 DNA-binding domain (BD). Co-transformation of PpIPD3-AD with PpCCaMK-BD facilitated robust growth on selective media, indicative of strong physical interaction. However, no growth or evidence for interaction was detected between PpIPD3-AD and PpCCaMKb-BD (Figure 1D). The lower expression levels of *PpCCaMKb* compared to *PpCCaMK*, along with the apparent inability of its gene product to bind PpIPD3, suggest that *PpCCaMKb* may encode a non-functional protein or function in a different context.

Legume CCaMKs have been characterized biochemically, and their autophosphorylation activity is known to be stimulated by elevated levels of calcium and inhibited by calmodulin (CaM) in the presence of high calcium levels (e.g., Miller et al., 2013). Based on the conservation of the autoactivation loop shown in Figure 1B, we predicted that PpCCaMK and/or PpCCaMKb would lead to similar activities *in vitro*. To test if either Physcomitrium CCaMK homolog showed calcium/CaM-dependent protein kinase activity, we purified recombinant PpCCaMK and PpCCaMKb, along with positive and negative controls, and assayed autophosphorylation activity using radiolabeled ATP. Autophosphorylation of purified PpCCaMK was detectable and enhanced in buffer containing free calcium ions compared to the EGTA control (Figure 1E). The autophosphorylation of PpCCaMK was attenuated in the presence of calcium and calmodulin, as described for Medicago CCaMK (Miller et al., 2013). No detectable kinase activity was observed for PpCCaMKb under the same conditions. These results demonstrated that PpCCaMK has retained similar calcium- and calmodulin-regulated kinase activity and suggested that PpCCaMKb may not be enzymatically active. To further assess whether PpCCaMK and/or PpCCaMKb are *bona fide* CCaMKs, we tested whether either could bind calmodulin (CaM) *in vitro*. Biotin-labeled CaM was applied to immobilized recombinant PpCCaMK and PpCCaMKb and detected by chemiluminescence to check for binding (Figure 1F). PpCCaMK showed similar CaM-binding activity levels to MtCCaMK, the positive control; however, PpCCaMKb showed nearly undetectable CaM-binding activity under the same conditions. Thus, consistent with gene expression and Y2H data, biochemical data supported a model wherein PpCCaMK but not PpCCaMKb comprises a functional signaling module with PpIPD3. Given the presence of this symbiosis signaling module in Physcomitrium, co-culture of wild-type moss with the model mycorrhizal fungus *Rhizophagus irregularis* was attempted. Still, no evidence of intracellular infection was obtained after six months of co-culture (Figure S4), consistent with the prevailing interpretation that Physcomitrium is not an AMF host plant.

### Heterologous expression of synthetically activated PpCCaMK stimulates symbiotic signaling in Medicago

Deleting the C-terminal autoinhibitory domains in Medicago or Lotus CCaMK leads to autoactivation and spontaneous nodule formation (Gleason et al., 2006). We investigated whether an equivalent deletion of the *PpCCaMK* C-terminus could promote spontaneous activation of the common symbiosis pathway by heterologous expression of native or modified PpCCaMK in Medicago roots. *M. truncatula* plants carrying the *pENOD11::GUS* reporter were transformed with constructs expressing *MtCCaMK*, *PpCCaMK*, or just the kinase domain of these proteins (*MtCCaMK*^*K*^ and *PpCCaMK*^*K*^*,* respectively). Plants transformed with a vector control either treated with *S. meliloti* LCOs or not were used as positive and negative controls, respectively. Roots expressing *MtCCaMK* and *PpCCaMK* did not exhibit any detectable *ENOD11* expression (Figure S5A). In contrast, roots expressing *MtCCaMK*^*K*^ and *PpCCaMK*^*K*^ not only expressed *MtENOD11* strongly but also elicited spontaneous nodules (Figure S5B), indicating that *PpCCaMK* is functionally capable of activating the symbiosis signaling pathway in Medicago.

### Complementation of symbiosis-defective phenotypes of Medicago *ccamk* loss-of-function mutants by heterologous expression of *PpCCaMK*

To further interrogate the functionality of PpCCaMK or PpCCaMKb *in vivo*, within the functional context of the symbiotic signaling pathway, we tested their ability to rescue the phenotype of Medicago *ccamk-1* mutants, which are defective for both nodulation and mycorrhization. ‘Hairy root’ genetic transformations mediated by *Agrobacterium rhizogenes* were used to introduce expression vectors containing the CDS from *MtCCaMK* (positive control), *PpCCaMK*, *PpCCaMKb*, or the empty vector (EV) negative control into roots of Medicago *ccamk-1* plants. A red fluorescent protein (RFP) visual marker was used to confirm that transformations were successful. To test for AMF colonization, transformed roots were inoculated with *Rhizophagus irregularis* and grown in co-culture for six weeks. Trypan blue staining was used to visualize arbuscules and revealed that roots transformed with vectors containing PpCCaMK or MtCCaMK formed arbuscules indicative of colonization. In contrast, roots transformed with PpCCaMKb or the EV did not show any instances of arbuscule formation (Figure 1G). To test for the ability to nodulate, transformed roots were inoculated with *Sinorhizobium meliloti* and co-cultured for two weeks. Whereas roots transformed with the EV or *PpCCaMKb* did not form any nodules, roots transformed with *PpCCaMK* formed nodules similarly to roots transformed with *MtCCaMK* (Figure 1H). These data corroborate the conservation of key functional features of CCaMK between legumes and mosses and demonstrate that PpCCaMK can decode symbiotic signals when heterologously expressed in legumes.

### *PpIPD3* partially rescues the symbiotic defects of Medicago *ipd3* mutants

To determine the extent to which*PpIPD3* can functionally substitute for *MtIPD3*, we assessed the ability of heterologously expressed *PpIPD3* to rescue the symbiotic defects of the *M. truncatula ipd3-1*mutant. Roots of the *ipd3-1*mutant were transformed with vectors driving transgenic expression of *MtIPD3* or *PpIPD3* or with an empty vector (EV) for a negative control. Roots of wild-type plants transformed with the empty vector were used as a positive control. All constructs also contained a tdTomato fluorescent reporter for the confirmation of transformation. In each case, roots were inoculated with the *Sinorhizobium meliloti* multi-reporter strain CL304 expressing a construct carrying both *hemA::lacZ* and *PnifH::GUS* (Lang et al., 2018a). As expected, based on the findings of Horváth et al. (2011), roots of the *Mtipd3-1* mutants transformed with the EV control developed nodules; however, few nodules were infected by rhizobia, and none of these nodules showed detectable expression nifH, in contrast to wild-type plants transformed with the same EV. Nodules produced on the *Mtipd3-1* mutants transformed with *MtIPD3* were similar to those on wild-type plants transformed with the EV, indicating a rescue of the symbiotic phenotype (Figure S6). Interestingly, the transformation of the *Mtipd3-1*mutant roots with *PpIPD3* only partially rescued the symbiotic defects with many colonized nodules observed, but none containing rhizobia showing expression of *nifH* (Figure S6). These findings indicate that *PpIPD3* contains some of the molecular features necessary for coordinating nodule infection but is not fully capable of restoring mutually beneficial symbiosis when heterologously expressed.

### Developmental reprogramming and brood cell formation associated with synthetic activation of CCaMK-IPD3 in Physcomitrium

**Figure 2:**
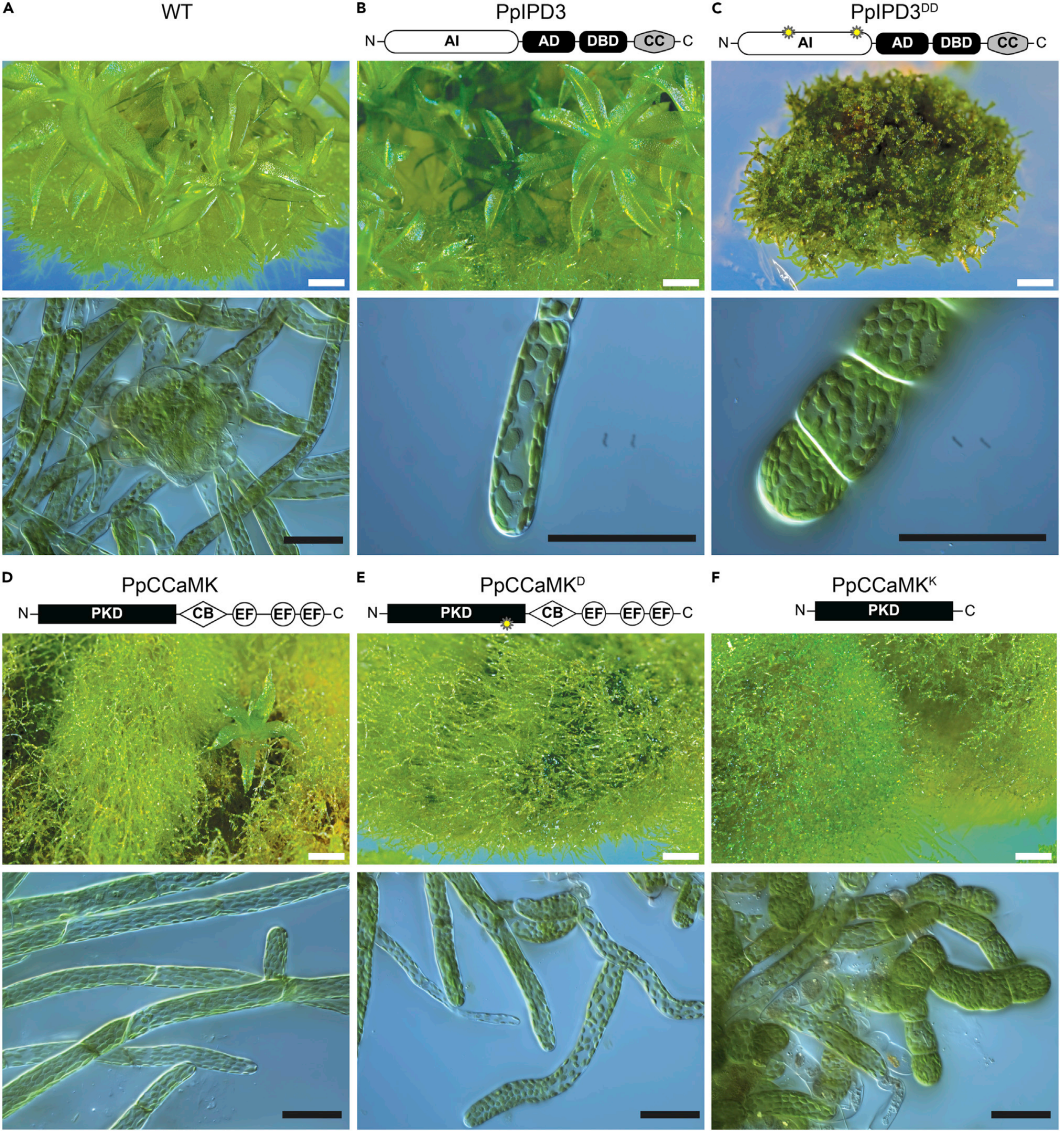
Ectopic development of brood cells in Physcomitrium expressing engineered, synthetically activated forms of PpCCaMK or PpIPD3 (A) Example of protonema and gametophores in wild-type Physcomitrium under standard *in vitro* growth conditions. (B) Lines transformed with unmodified PpIPD3 driven by a *Zea mays UBIQUITIN1* (ZmUBI1) promoter did not display abnormal gametophore or protonemal morphology. (C) Lines transformed with a modified *PpIPD3* (*PpIPD3*^*DD*^) carrying two phosphomimetic substitutions in the autoinhibitory domain, driven by the same promoter, constitutively formed brood cells and failed to develop gametophores. (D) Lines transformed with the native form of *PpCCaMK* developed excess protonema and fewer gametophores compared to WT controls, but protonemal morphology was not strongly affected. (E) Lines transformed with a modified *PpCCaMK* containing a phosphomimetic substitution in the regulatory domain (*PpCCaMK*^*D*^) typically did not develop any gametophores, and gametophores that did develop were stunted (see Figure S7B). Brood cells were commonly found among protonema under unstressed conditions (see Figures S7B and S7C) (F) Lines transformed with a modified *PpCCaMK* with the C-terminal regulatory region deleted (*PpCCaMK*^*K*^) constitutively developed brood cells under standard growth conditions. Constructs were driven by the same promoter. Samples were grown in BCDAT medium under the same conditions. Images are representatives of 4-week- (top) or 2-week-old (bottom) subcultured lines. White scale bars = 500 μm. Black scale bars = 50 μm

**Figure 3:**
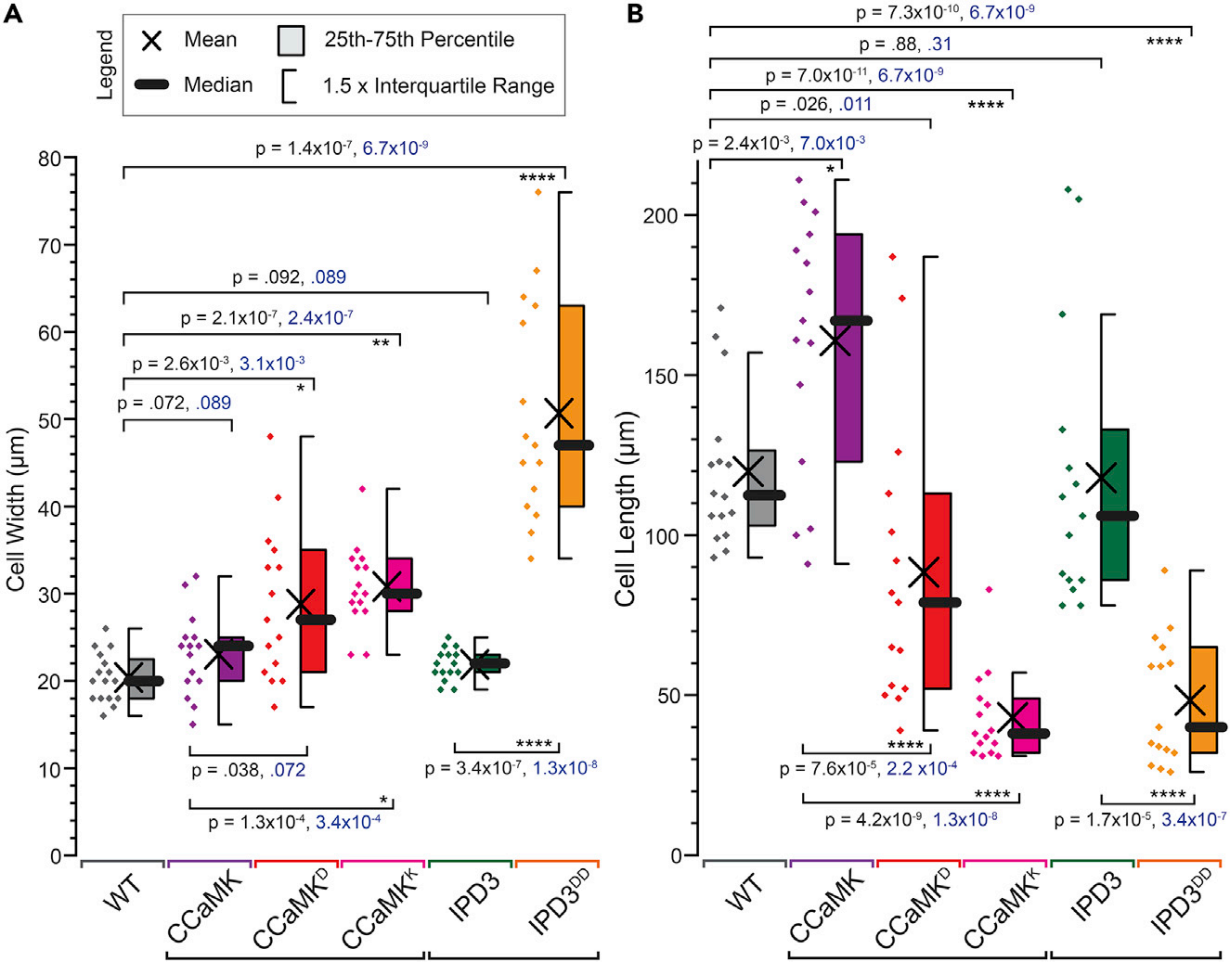
Quantification and statistical analysis of protonemal cell dimensions of lines expressing native or modified forms of CCaMK or IPD3 (A) Measured protonemal cell widths of gain-of-function CCaMK or IPD3 lines in comparison to controls expressing unmodified forms or WT. Statistics summarized by legend. (B) Measured protonemal cell lengths of gain-of-function CCaMK or IPD3 lines in comparison to controls expressing unmodified forms or wild-type (WT). Measurements were taken of protonemal cells at the edges of approximately 3-week-old cultures. Apical cells were excluded. Welch’s t-test was used to calculate p-values displayed in black; and Mann-Whitney U-test was used to calculate p-values shown in blue (n = 15–16 cells total from three independent transformants). Note that each comparison was pre-planned (i.e., non-exploratory). One-way ANOVA analyses of the same datasets were performed for comparison. Asterisks indicate significance levels (∗: p ≤ 0.05; ∗∗: p ≤ 0.01; ∗∗∗: p ≤ 0.001; ∗∗∗∗: p ≤ 0.0001). Full ANOVA results provided in Table S2.

**Figure 4:**
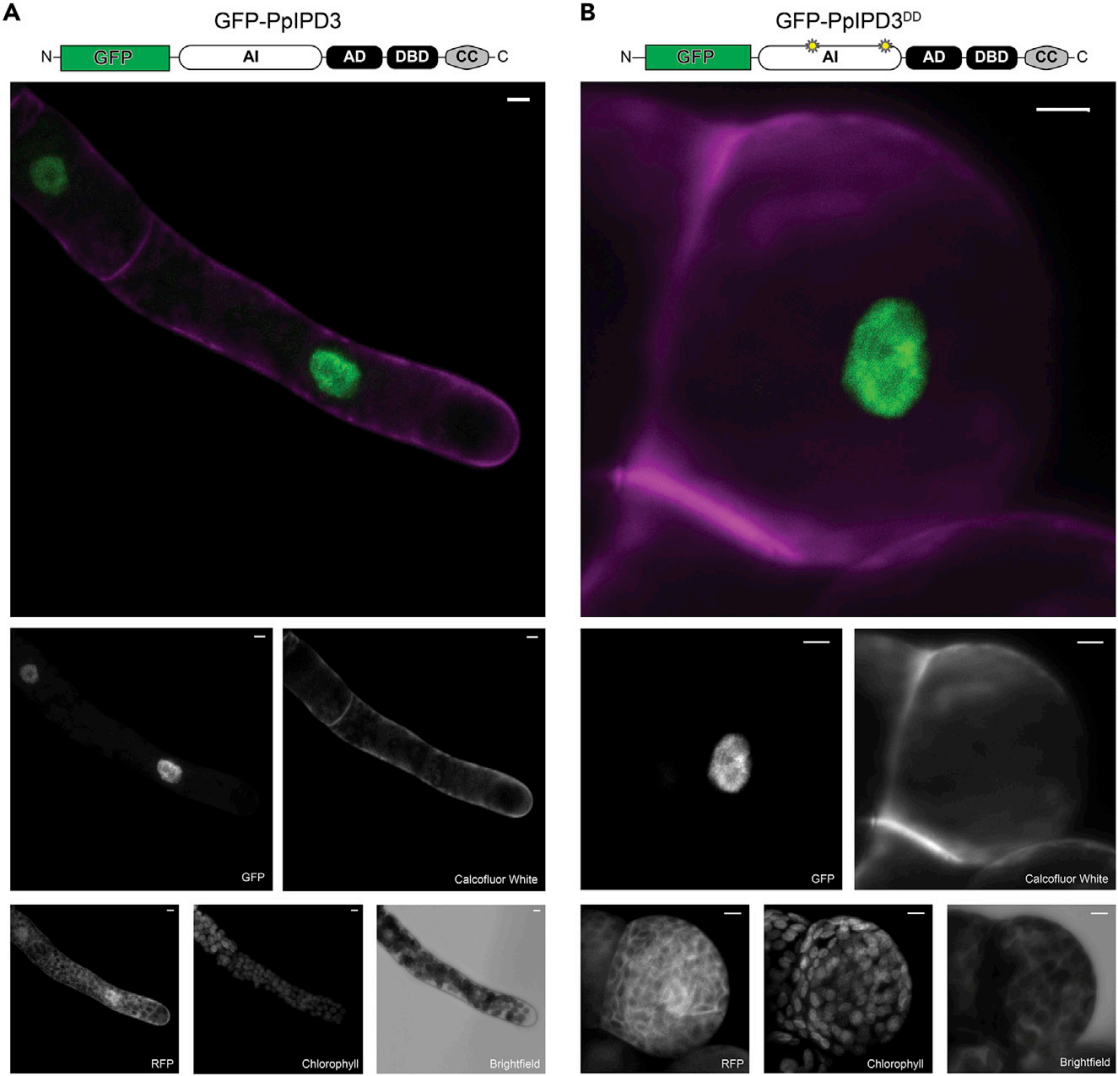
Subcellular localization of GFP-tagged PpIPD3 and PpIPD3^DD^ driven by *Zea maysUBIQUITIN1* promoter Images are maximal projections of confocal z-stack acquisitions. Color images show merged GFP (green) and calcofluor white (magenta) channels. Individual channels are labeled below. Free soluble red fluorescent protein (RFP) and chlorophyll autofluorescence were imaged for comparison. (A) N-terminally GFP-tagged native IPD3 localizes to nuclei, and protonemal growth and development is not noticeably affected. (B) N-terminally GFP-tagged native IPD3^DD^, which differs by only two amino acid substitutions, also localizes to the nucleus and drives ectopic development of brood cells under non-stressed conditions. Images are intended strictly for qualitative spatial distribution and were not acquired under identical acquisition settings or rendered under identical brightness and contrast settings. All scale bars = 5 μm

Previous studies in legumes have shown that mutated forms of CCaMK or IPD3 are sufficient to cause striking gain-of-function phenotypes: the development of nodules or the pre-penetration apparatus in the absence of rhizobial or mycorrhizal symbionts (Gleason et al., 2006; Singh et al., 2014; Takeda et al., 2012; Tirichine et al., 2006). We introduced equivalent amino acid substitutions or deletions into PpCCaMK or PpIPD3 to engineer predicted gain-of-function variants. Native or modified forms (hereafter referred to to as PpCCaMK^K^, PpCCaMK^D^, and PpIPD3^DD^) were transgenically expressed in Physcomitrium under the control of a maize ubiquitin (ZmUBI1) promoter. Transgenes were delivered by particle bombardment, as described in a previous study (Kleist et al., 2017). A minimum of eight independently transformed lines were examined for phenotypic consistency (Table 1). Expression of unmodified PpIPD3 in this manner did not cause any noticeable effects on the development or morphology of protonemata or gametophores under standard axenic growth conditions, as these lines closely resembled wild-type Physcomitrium or empty vector controls (Figures 2A, 2B, Figure S7A). Under identical conditions, lines expressing the predicted constitutively active variant, PpIPD3^DD^, driven by the same promoter, developed branched chains of slowly growing, nearly isodiametric cells with dense chloroplasts and prominent cell wall thickenings (Figures 2C, S7B and S7C). These features are diagnostic of brood cells, which are stress-resistant asexual propagules found in mosses (Correns, 1899; Duckett and Ligrone, 1992; Pressel and Duckett, 2010; Schnepf and Reinhard, 1997). Lines expressing PpIPD3^DD^ failed to form normal chloronema or caulonema and did not develop gametophores (Figure S7D). Transformants expressing unmodified PpCCaMK displayed typical protonemal morphology and were able to develop gametophores, albeit with reduced frequency and size (Figure 2D). Brood cell formation was not observed in lines expressing unmodified PpCCaMK under standard growth conditions. Transformants expressing a phosphomimetic variant, PpCCaMK^D^, developed mixed populations of phenotypically normal protonema and brood cells under standard growth conditions (Figures 2E, S7E). Gametophores were rarely observed and, when present, were stunted and malformed (Figure S7F). Lines expressing PpCCaMK^K^ showed similar but more severe phenotypes, with frequent brood cell development and scarce instances of gametophore formation (Figure 2F). Quantitative analysis of protonemal cell dimensions revealed highly statistically significant differences in cell length and width for lines expressing gain-of-function forms of PpCCaMK or PpIPD3 compared to untransformed lines or lines expressing the native form of PpCCaMK or PpIPD3 (Figure 3, Table S2). Quantitative real-time PCR analysis showed elevated transcript abundances for each of the transgenically expressed forms of PpCCaMK and PpIPD3, demonstrating that transgenes were transcribed (Figure S8). Expression of native IPD3 or gain-of-function PpIPD3^DD^ tagged with green fluorescent protein (GFP) using the same vector demonstrated that protein product is present in either case, causes similar phenotypes to expression of untagged forms, and fusion proteins shows preferential localization to nuclei (Figure 4). The developmental phenotypes that we observed in CCaMK and IPD3 gain-of-function lines, particularly the constitutive development of brood cells, which generally only occurs in response to stress, led us to hypothesize that the Physcomitrium CCaMK-IPD3 module functions in developmental reprogramming to mediate resistance to or escape from stress conditions.

**Table 1:** Modified forms of PpCCaMK and PpIPD3 used in this study

Name	Mutation(s)	Predicted Effect	# Transformants	Reference(s)
PpCCaMK	None	–	12	–
PpCCaMK^D^	S252D	Constitutive Activation	8	Banba et al., 2008; Takeda et al. (2012)
PpCCaMK^K^	Δ307-504	Constitutive Activation	14	Gleason et al. (2006)
PpIPD3	None	–	18	–
PpIPD3^DD^	S107D, S241D	Constitutive Activation	>36	Singh et al. (2014)

The name, introduced mutations (if applicable), and predicted effects of introduced mutations are listed. For each construct, the number (#) of independent transformants that were generated and analyzed is provided. The reference for each study that guided our directed mutagenesis are provided and cited in the main text. Delta (Δ) indicates deletion. Dashes (−) indicate not applicable.

### Elevated levels of ABA and *LATE EMBRYOGENESIS ABUNDANT* transcripts in physcomitrium expressing synthetically activated forms of CCaMK or IPD3

**Figure 5:**
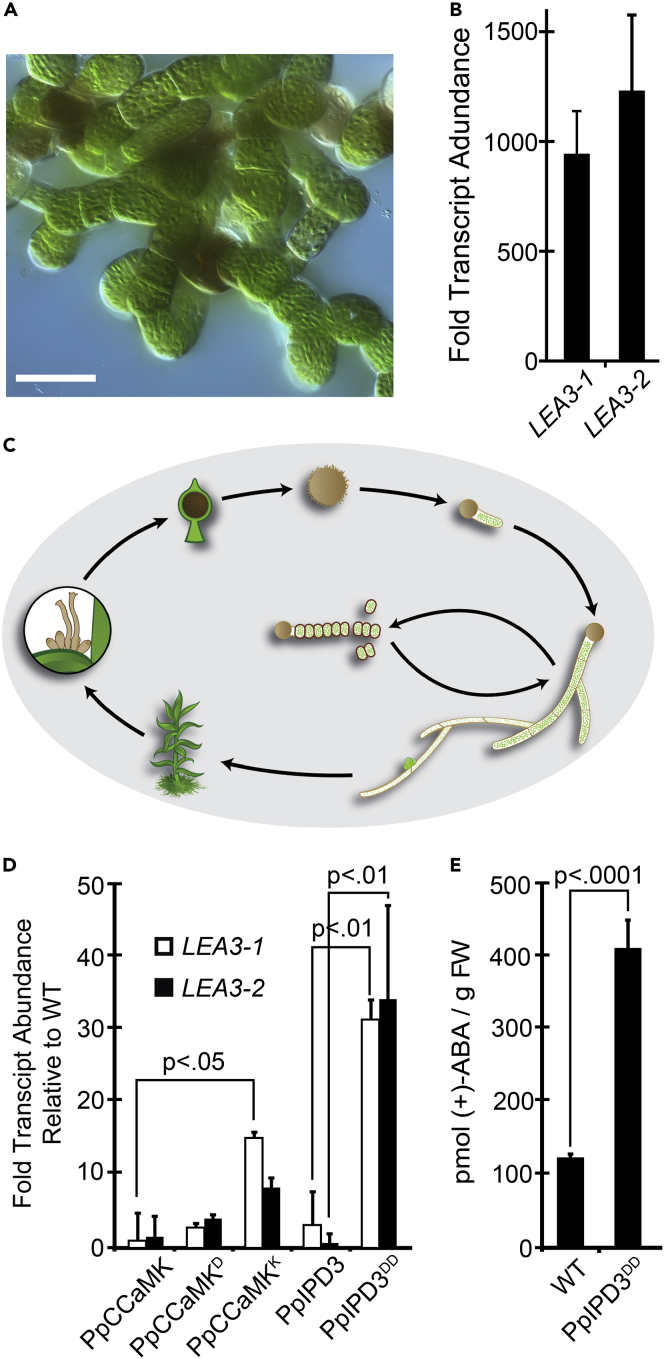
Phenotypic comparison of Physcomitrium CCaMK-IPD3 gain-of-function lines to ABA-treated WT protonema and hypothetical model for CCaMK-IPD3 function (A) WT protonema developed brood cells under unstressed conditions in BCDAT growth medium within two weeks of treatment with 100 μM ABA. Scale bar = 50 μm. (B) Quantitative reverse transcription PCR (RT-qPCR) demonstrated that WT protonemal tissues treated with ABA contain elevated transcript levels for the ABA response marker genes *LEA3-1* and *LEA3-2*. Error bars indicate the SE of the mean (SEM) among treatments. (C) Illustrated life cycle of Physcomitrium. Spores (top) germinate and give rise to chloronema, which give rise to caulonema and gametophores. Gametangia develop in leaf axils of gametophores, and motile sperm swim through the environment to achieve fertilization. The mature zygote forms a spore capsule. From germination until fertilization, the moss is dependent on locally available water. Under stress conditions (e.g., drought), brood cells serve as stress-resistant ‘vegetative spores’ that fragment to facilitate dispersal. Brood cells germinate upon relief from stress; the life cycle resumes with the development of chloronema. (D) RT-qPCR analyses indicated that activation of the CCaMK-IPD3 signaling module is associated with elevated transcript levels for the ABA response marker genes *LEA3-1* and *LEA3-2*. Error bars indicate SEM among biological replicates. Results were statistically evaluated using the Tukey honestly significant difference (HSD) test. The p-values that indicated statistical significance below a threshold of 0.05 are annotated. (E) Enzyme-linked immunosorbent assays (ELISAs) revealed substantially elevated levels of (+)-ABA in protonemal tissues of lines expressing PpIPD3^DD^ compared to wild-type controls under standard *in vitro* growth conditions. Error bars indicate SEM among three biological replicates each from two independently transformed lines. The p-value was obtained using a two-sample Student’s t-test.

The stress-associated phytohormone abscisic acid (ABA) has long been linked to the induction of brood cells (Bopp, 2000; Schnepf and Reinhard, 1997). As expected, treatment of wild-type protonema with ABA phenocopied the gain-of-function effects of PpCCaMK^K^ or PpIPD3^DD^ and stimulated the development of brood cells (Figure 5A). Quantitative RT-PCR was used to test whether stress-associated, ABA-inducible marker genes were likewise upregulated in CCaMK-IPD3 gain-of-function lines. We selected two previously described marker genes, *LEA3-1* and *LEA3-2*, which encode late embryogenesis abundant (LEA) proteins (Shinde et al., 2012, 2013) and confirmed that transcript levels were elevated in wild-type protonemata treated with exogenously supplied ABA (Figure 5B). Initially characterized in seeds, LEA proteins serve as osmoprotective molecules and are thought to confer abiotic stress resistance in brood cells, thereby enhancing their dispersal ability (Figure 5C). Transgenic lines that constitutively form brood cells accumulated elevated levels of *LEA3-1* and *LEA3-2* under standard growth conditions (i.e., in the absence of any stress agent) relative to wildtype (Figure 5D). Transcript levels were more abundant in gain-of-function lines compared to lines expressing unmodified PpCCaMK or PpIPD3. For example, expression of PpCCaMK^K^ was associated with significantly higher levels of *LEA3-1* transcript compared to lines expressing native PpCCaMK from the same promoter (p < .01). As observed for developmental phenotypes, expression of PpIPD3^DD^ had the most substantial effect on *LEA* transcript abundance, and the accumulation of *LEA3-1* and *LEA3-2* transcripts was significantly higher in lines expressing PpIPD3^DD^ compared to lines expressing PpIPD3 (p < .05), providing further evidence for a functional link between activation of the Physcomitrium CCaMK-IPD3 module and ABA signaling.

The observed phenotypic similarities between ABA-treated wild-type Physcomitrium and PpCCaMK-IPD3 gain-of-function lines may, in theory, be caused by an increase in ABA accumulation, an increase in ABA sensitivity, activation of a different pathway with similar effects, or a combination of these scenarios. To further investigate the mechanism whereby the CCaMK-IPD3 module elicited these responses, we quantified ABA levels in tissues overexpressing IPD3^DD^ compared to wildtype by ELISA (enzyme-linked immunosorbent assays). The results showed that IPD3^DD^ gain-of-function lines contained significantly higher ABA levels than wildtype (Figure 5E), suggesting that the ABA-associated responses we observed are likely due, at least in part, to increased ABA accumulation.

### Brood cell formation in Physcomitrium *ccamk* and *ipd3* loss-of-function mutants

**Figure 6:**
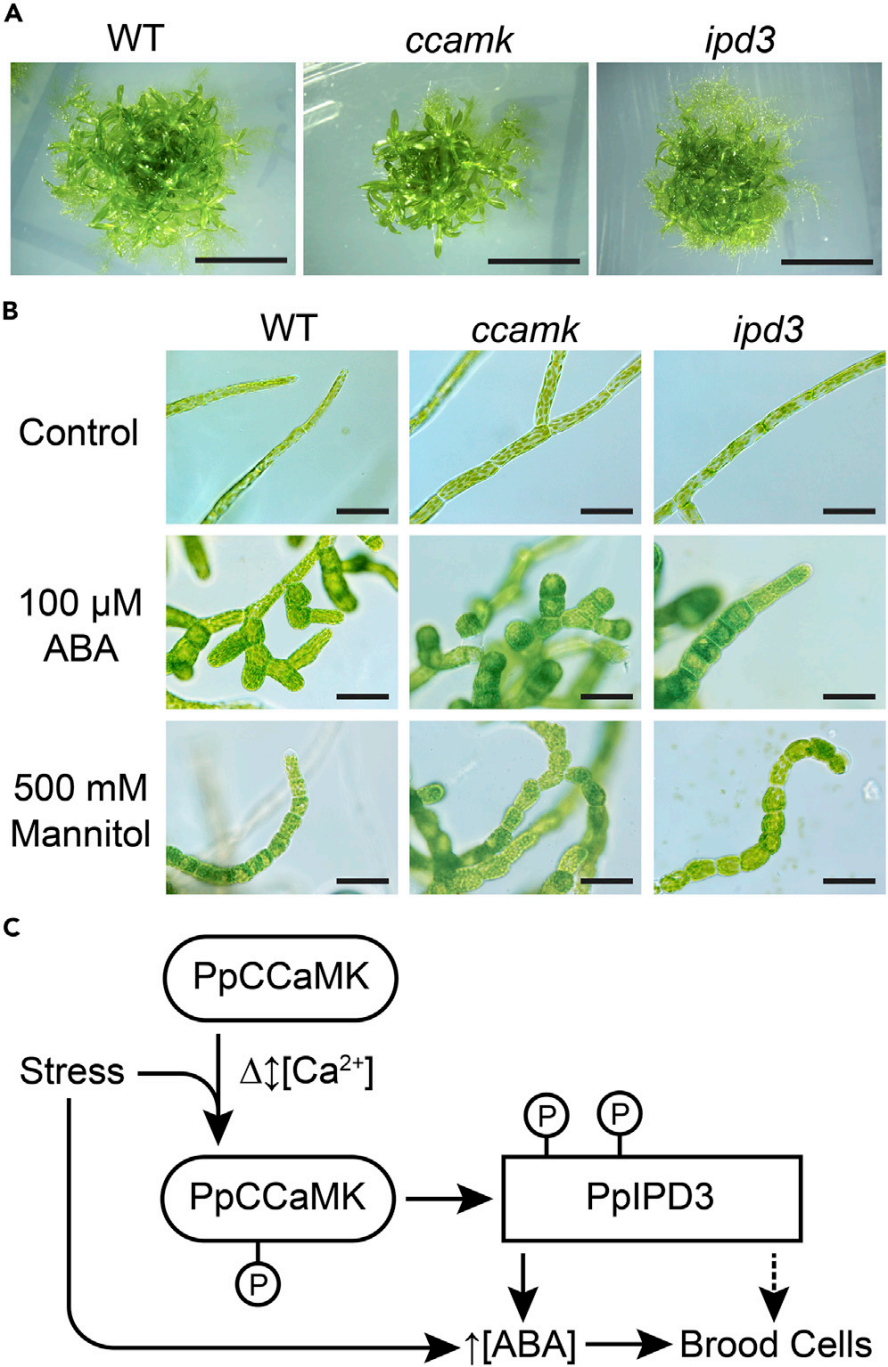
Neither CCaMK nor IPD3 is required for brood cell formation under ABA treatment or osmotic stress conditions (A) Physcomitrium *ccamk* and*ipd3* deletion mutants did not show any obvious phenotypic aberrations relative to WT under standard *in vitro* growth conditions in the BCDAT medium. Scale bar = 500 mm (B) Physcomitrium *ccamk* and *ipd3* deletion mutants were able to develop brood cells when 100 μM ABA was added to BCDAT medium under standard growth conditions or when hyperosmotic stress was applied by addition of 500 mM mannitol to the growth medium. Treatments were performed for two weeks before images were taken. Two independently generated deletion mutant lines each were tested for *IPD3* and *CCaMK* with similar results. Data from a single experiment are shown. Scale bar = 50 μm. (C) Diagram showing a hypothetical model for PpCCaMK-IPD3 function during stress signaling in relation to ABA accumulation and brood cell formation. In this model, stress conditions provoke changes in nucleocytoplasmic Ca^2+^ levels, leading to activation of PpCCaMK. Trans-phosphorylation of PpIPD3 renders it active and leads to elevated levels of ABA and the development of brood cells. The ability of *ccamk* and *ipd3* deletion lines to develop brood cells in response to tested stress treatments indicates that there are likely other pathways that trigger stress-induced ABA accumulation. The dotted line indicates uncertainty whether PpIPD3 acts exclusively through ABA signaling to promote brood cell development

To investigate if the CCaMK-IPD3 module is required to develop brood cells, we assayed responses to stress treatments in deletion lines lacking either the *CCaMK* or *IPD3* genomic locus. Each locus was deleted by homologous recombination with antibiotic selective markers. Disruption of the respective locus was confirmed by PCR genotyping using genomic DNA and by RT-PCR (Figure S9). Deletion lines displayed stereotypical protonematal and gametophore morphology when grown under standard conditions (Figures 6A and 6B). When treated with ABA or hyperosmotic media supplemented with mannitol, multiple independently generated *ccamk* and *ipd3* knockout lines responded similarly to wild-type controls by developing brood cells, which we did not observe in wild-type moss under standard laboratory growth conditions (Figure 6B). These results suggest that while sufficient to stimulate ABA accumulation and brood cell formation, the CCaMK-IPD3 module is not required for brood cell development in response to ABA or osmotic stress treatments. We did not observe any noticeable differences in levels of brood cell formation between mutant and wild-type on both treatments, indicating that genetic perturbation of the CCaMK-IPD3 module does not substantially alter the sensitivity of Physcomitrium to ABA. The lack of phenotypic defects in *ccamk* or *ipd3* deletion lines may imply functional redundancy in stress-induced developmental programming. Our results are collectively consistent with the hypothesis that the CCaMK-IPD3 module operates in the context of a broader signaling network that mediates stress-responsive developmental reprogramming in Physcomitrium (Figure 6C).

### Nuclear calcium oscillations in Physcomitrium protonemata

**Figure 7:**
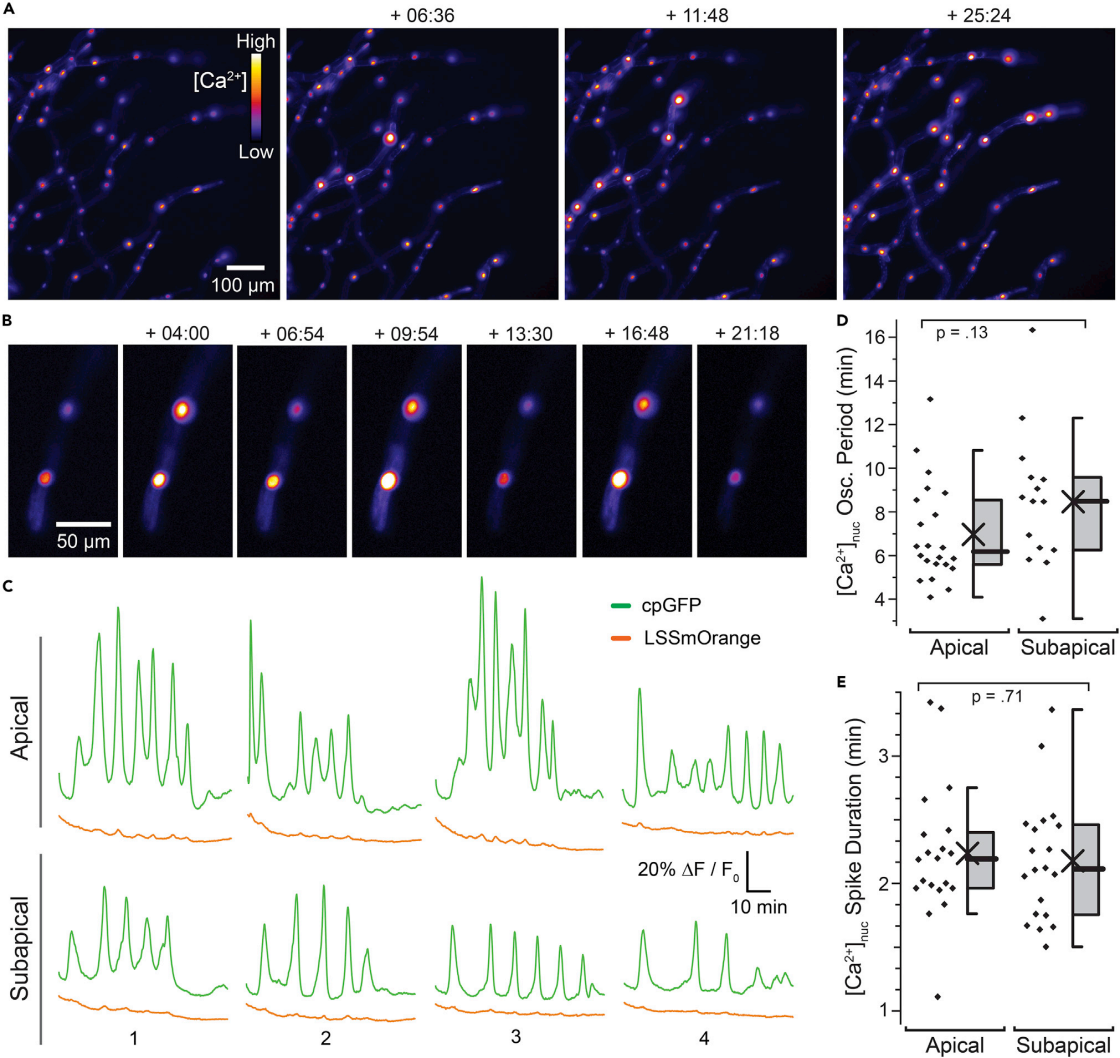
Spontaneous calcium spiking in nuclei of Physcomitrium protonemata (A) Calcium imaging of protonemata expressing NLS-GCaMP6s. Maximum z-stack projection is presented in ‘Fire’ lookup table (LUT). Fluorescence intensity and brightness of coloration are positively correlated with nuclear calcium concentration ([Ca^2+^]_nuc_). Some fluorescence in the cytosol was also observed. Timestamp is in minute:second format. See Video S1 for complete timelapse acquisition. (B) Calcium imaging of protonemata expressing NLS-MatryoshCaMP6s. Maximum z-stack projections of reporter circularly permutated GFP fluorescence are shown. Timestamp and LUT presented in same format as (A). See Video S2 for complete timelapse acquisition. (C) Timecourse quantification of manually drawn nuclear regions of interest (ROIs) in protonemata expresing NLS-MatryoshCaMP6s. Relative changes in fluorescence intensity (ΔF/F_0_) for reporter cpGFP channel (green) and reference LSSmOrange (orange) channel are shown. Four ROIs each for apical (growing tip) cells and subapical (secondary cells adjacent to growing tip) are shown as examples. Additional examples from an independent experiment using NLS-GCaMP6s are shown in Figure S10. (D) Quantitative analysis of nuclear calcium oscillation ([Ca^2+^]_nuc_ Osc.) periods shown in minutes (min). Chi: mean. Bar: Median. Box: 25th - 27th percentile. Whiskers: 1.5 x IQR. Displayed p-value was calculated using Welch’s t-test (n = 15–22). (E) Quantitative analysis of [Ca_2+_]_nuc_ spike durations (defined here as time from half-maximal rise to half-maximal decay). Statistical symbols are formatted identically to (D). Displayed p-value was calculated by Welch’s t-test (n = 20 spikes from four different nuclei). Experiments were independently replicated more than seven times with similar results. See Figure S10 for additional related data and STAR Methods for details of imaging setup

In legumes and other plants, the CCaMK-IPD3 signaling module is activated by nuclear calcium oscillations elicited by nod or myc factors. We therefore deployed nuclear targeted genetically encoded calcium indicators (GECIs) in Physcomitrium and sought to identify conditions or treatments that led to elicitation of nuclear calcium oscillations. In lines expressing nuclear-targeted intensiometric GCaMP6s (Chen et al., 2013), we unexpectedly observed prominent spontaneous calcium spiking in protonematal nuclei without performing any experimental treatment (Figure 7A, Figure S10, Video S1). To corroborate these observations, we repeated these experiments using MatryoshCaMP6s, which contains a stable internal reference fluorophore (Ast et al., 2017). We observed similar spontaneous spikes using MatryoshCaMP6s (Figure 7B, Video S2). Quantification showed that fluorescence intensity changes were pronounced in the reporter circularly permutated GFP channel but not in the reference LSSmOrange channel, which is consistent with spontaneous oscillatory calcium concentration changes in protonematal nuclei (Figure 7C). Average oscillatory periods we observed were approximately seven and a half minutes (mean: 7.6 min, SEM: 0.45 min, median: 6.4 min, n = 37 nuclei). Average nuclear calcium spike duration was approximately 2 minutes (mean: 2.2 min, SEM: 0.078 min, median: 2.2 min, n = 40 spikes from eight different nuclei). We did not observe any statistically distinguishable differences in oscillation period between apical and subapical cells (Figure 7D) nor in calcium spike duration, which we took as time from half-maximal rise to half-maximal decay (Figure 7E). Similarly, we did not observe statistically significant difference between apical and subapical cells in calcium spike amplitude (Figure S10B). Thus, we uncovered robust evidence that nuclear calcium spiking occurs in protonemata without application of any chemical elicitors, suggesting unexpected complexity in the nuclear calcium signaling code in Physcomitrium.

Video S1. Spontaneous calcium spiking in Physcomitrium protonematal nuclei detected using GCaMP6s, related to Figure 7and Figure S10 Calcium dynamics were recorded using GCaMP6s targeted for nuclear enrichment using an N-terminal nuclear localization sequence. Sensor is apparently present in the cytosol as well as nuclei. Movie shows z-stack projections displayed using the Fire lookup table (FIJI). Time stamp is in minute:second format.

Video S2. Spontaneous calcium spiking in Physcomitrium protonematal nuclei detected using MatryroshCaMP6s, related to Figure 7 and Figure S10 Calcium dynamics were recorded using MatryoshCaMP6s targeted for nuclear enrichment using an N-terminal nuclear localization sequence. The circularly permutated GFP channel is shown. Sensor is detectable in the cytosol as well as nuclei. We hypothesize that observed increase in chloroplast fluorescence (starting after the 30 minute mark) is due to autofluorescence and may be indicative of light stress. Movie shows z-stack projections displayed using the Fire lookup table (FIJI). Time stamp is in minute:second format.

## Discussion

### Evolution of the CCaMK-IPD3 signaling module across land plants

Many of the critical components of the symbiosis pathway were present in the algal ancestors of land plants, indicating that they have been vertically inherited across land plants (Delaux et al., 2015). Across evolutionary time, spanning from the divergence of bryophytes to the emergence of angiosperms, AMF-like interactions have remained morphologically similar (Remy et al., 1994; Strullu-Derrien et al., 2014). In light of plant comparative genomics of AMF-host versus non-host lineages, the symbiosis pathway in the earliest land plants likely contributed to the recognition and intracellular infection of AMF, as the presence/absence of the symbiosis pathway is strongly correlated with host/non-host status, respectively (Delaux, 2017; Delaux et al., 2014; Garcia et al., 2015; Kamel et al., 2016). Among embryophytes, the moss clade is a striking exception to this genomic signature. To date, there has been no demonstration of the mutualistic transfer of nutrients between mosses and AMF. On the contrary, endophytic fungal interactions described in mosses have appeared restricted to dead or senescing tissues (Pressel et al., 2010). This peculiarity piqued our interest in the conserved components of the common symbiosis pathway in mosses.

In the present study, we investigated the functional reason for retaining the CCaMK-IPD3 signaling module in non-mycorrhizal mosses, which stands in stark contrast to multiple independent losses of these genes in non-mycorrhizal angiosperm and liverwort lineages. Biochemical and mutant-rescue assays demonstrate that the biochemical activities of CCaMK and IPD3 are conserved broadly throughout land plants, which suggests that CCaMK may decode similar oscillatory calcium signals in bryophytes. The biochemical similarity of Physcomitrium CCaMK to homologs in angiosperms is consistent with a previously published *in vitro* comparison (Okada et al., 2003). We used a gain-of-function strategy in Physcomitrium to shed light on the physiological consequences of CCaMK-IPD3 activation. The developmental phenotypes observed in Physcomitrium cells expressing CCaMK and IPD3 carrying predicted gain-of-function mutations imply a functional link between the CCaMK-IPD3 module and ABA signaling in mosses. The heterologous expression of native and engineered forms of PpCCaMK or IPD3 in Medicago roots corroborated the predicted effects of gain-of-function mutations and demonstrated partial functional conservation with Medicago homologs. The most striking finding in this study is the developmental phenotype of IPD3^DD^-expressing Physcomitrium lines: IPD3^DD^ transgenics displayed prolific, nearly constitutive formation of brood cells, mainly to the exclusion of other cell types. The effect does not appear to be attributable merely to overexpression, as controls transformed with the same vectors differing only by two codon changes to introduce phosphomimetic substitutions putatively. The observation that gain-of-function lines expressing modified forms of CCaMK showed a less severe phenotype than lines expressing modified ones might simply reflect a signaling bottleneck of natively expressed IPD3 upon the activities of expressed CCaMK. Comparative genomics across green plants have shown that core ABA signaling components are conserved in bryophytes (Wang et al., 2015). Although ABA biosynthesis and downstream signal transduction have been studied in the context of moss protonemal development (Komatsu et al., 2013; Schnepf and Reinhard, 1997; Shinde et al., 2012; Takezawa et al., 2015; Vesty et al., 2016; Yotsui et al., 2013), our data provide the first link between CCaMK-IPD3 and ABA signaling in Physcomitrium.

### Calcium signaling in Physcomitrium protonemata

We propose a model wherein calcium-dependent activation of CCaMK and trans-phosphorylation of IPD3 in Physcomitrium leads to ABA accumulation and brood cell formation. The rescue of Medicago *ccamk-1* mutants by *PpCCaMK* seemingly suggests that PpCCaMK may be activated by similar oscillatory calcium signals, as seen in legumes in response to symbionts. Additionally, the observed partial rescue of the Medicago *ipd3-1*mutant indicates partial conservation of function between PpIPD3 and MtIPD3, with PpIPD3 likely retaining the ability to be activated by CCaMK and stimulate expression of some (but perhaps not all) known downstream transcription factors. Whereas calcium oscillations in growing protonemal tips have been well documented (e.g., Bascom et al., 2018), nuclear calcium oscillations and potential elicitors have not been previously described in Physcomitrium. Hyperosmotic stress has been shown to elicit a pronounced transient elevation of cytosolic calcium levels. However, this response was neither oscillatory nor predominantly restricted to the nuclear region (Kleist et al., 2017). Recently, Galotto et al. (2020) reported that chitin can elicit oscillatory calcium signals in Physcomitrium, although these oscillations appear to occur primarily in the cytosol.

In legume root cells, nuclear calcium spiking is elicited by specific chemical signals from potential symbionts (Ehrhardt et al., 1996; Chabaud et al., 2011). We therefore posited that nuclear-targeted GECIs may allow us to screen for treatments expected to activate Physcomitrium CCaMK-IPD3 but unexpectedly found that protonematal nuclei exhibited repetitive calcium spiking absent any experimental treatment. Observed oscillations were neither restricted to nor noticeably distinct in nuclei of apical cells, therefore, they did not appear to be associated with mechanical stimuli caused by tip growth, as has been described in pollen tubes (Moser et al., 2020). It will therefore be highly informative for future studies to examine the activation mechanism of Physcomitrium CCaMK and decipher the calcium signatures it decodes in the context of moss protonemata. It is noteworthy that excitation of GECIs with blue light may have inadvertently stressed cells during our calcium imaging experiments or participated in elicitation of nuclear calcium oscillations. We noticed increased autofluorescence from chloroplasts during acquisitions extending over roughly 30 min (Videos S1 and S2), suggesting cell stress that may also influence calcium oscillations. Blue light has been linked to calcium signaling in plants, including Physcomitrium (Russell et al., 1998; Stoelzle et al., 2003); and, interestingly, blue light can have inhibitory effects on nodulation in Lotus (Shimomura et al., 2016).

While our observations open new avenues for inquiry, further work is needed to identify factors involved in the coding and decoding stress-induced calcium signals in moss. Specifically, a central question for further investigation identified by this study is how nuclear calcium oscillations are coupled to CCaMK activation status and phospho-regulation of IPD3 in Physcomitrium. This topic has been extensively investigated and modeled in the context of legume symbioses with rhizobia and mycorrhiza (e.g., Kosuta et al., 2008; Miller et al., 2013). Our findings collectively implicate a different physiological function and possibly a more complex decoding mechanism for CCaMK-IPD3 in Physcomitrium, given that nuclear calcium oscillations appear to occur constitutively without any apparent evidence for CCaMK-IPD3 activation (e.g., ABA-associated responses or brood cell formation). Inspired by engineering of optical reporters from mammalian CaMKII (e.g., Bossuyt and Bers, 2013), development of a fluorescent biosensor from Physcomitrium CCaMK that reports the active conformation of the kinase would provide a powerful tool to more precisely examine the physiological function of the moss CCaMK-IPD3 signaling module. A similar strategy could be pursued using IPD3, although could be complicated as almost nothing is known about IPD3 structure except for a coiled-coil domain. Förster resonance energy transfer (FRET) between fluorophores separately tagged to CCaMK and IPD3, as implemented in a previous study in Lotus (Singh et al., 2014), may also be an effective strategy to pinpoint stimuli that endogenously activate CCaMK-IPD3 in Physcomitrium.

### Physiological function of CCaMK-IPD3 in moss and elicitation of brood cells

The established physiological function of brood cells is to serve as stress-resistant asexual propagules that break away from parent plants and enable mosses to escape osmotic stress and dehydration. For this reason, they have also been referred to to as ‘vegetative spores.’ The phenotypes of Physcomitrium CCaMK and IPD3 gain-of-function lines suggest that these components may also be linked to osmotic stress and dehydration responses. Because osmotic stress and dehydration are closely related to oxidative stress, it is worth noting that CCaMK has been associated with oxidative stress responses in other plants. CCaMK expression is induced by ABA or oxidative stress in rice; CCaMK was also required for ABA-mediated antioxidant responses (Shi et al., 2012, 2014). Similarly, CCaMK has been reported to be activated by nitric oxide and required for ABA-mediated antioxidant activity in maize (Ma et al., 2012; Yan et al., 2015). In wheat (*Triticum aestivum*), CCaMK expression is modulated by ABA and osmotic stress, likely through the activity of numerous predicted ABA-response elements in its promoter region (Yang et al., 2011). Moreover, we noticed using the Physcomitrella Expression Atlas Tool (PEATmoss) that CCaMK transcript abundance is elevated under heat stress conditions (Fernandez-Pozo et al., 2020). In light of these observations, the CCaMK-IPD3 may play a role in abiotic stress acclimation in Physcomitrium and other plants. This possibility may explain retention of the CCaMK-IPD3 signaling module in non-mycorrhizal mosses.

The cell wall thickenings, lipid reserves, and enhanced dispersal ability of brood cells could conceivably be useful in evading pathogenesis, although this idea is presently unsubstantiated. Fungal pathogens, as well as oomycetes, have been shown to induce reactive oxygen species (ROS) production, cell-wall depositions (including callose depositions mediated in part through ABA signaling), and altered fatty acid metabolism (Oliver et al., 2009; Ponce de León, 2011; de León et al., 2015). There are also mechanistic links between pathogen perception and the symbiosis pathway in angiosperms. In rice, chitin-receptor *cerk1* mutants were impaired in both mycorrhizal and blast fungus infections (Miyata et al., 2014), and CERK1 is conserved as a chitin-induced immunity signaling receptor in Physcomitrium, possibly hinting at a further link between the common symbiosis pathway and immunity signaling (Bressendorff et al., 2016). These observations may warrant further investigation into the possibility that brood cells and the CCaMK-IPD3 pathway could also serve a heretofore unnoticed function in moss acclimation to biotic stresses.

Functional dissection of possible contributions of CCaMK-IPD3 to stress signaling in Physcomitrium will require further investigation and will likely rely on combining loss-of-function mutations and/or extensive screening of stress treatments (or combinations of stress treatments). We hypothesize that functional redundancy may occur through an ABA-dependent pathway in addition to the CCaMK-IPD3 pathway investigated in this study and that there may be crosstalk among stress signaling pathways upstream of stress-associated developmental reprogramming and brood cell formation. A similarly complex scenario has been described for stress signaling in angiosperm guard cells, wherein ABA and calcium function in a partially independent yet also synergistic manner (Webb et al., 2001; Huang et al., 2019; Schulze et al., 2021). In addition to identifying putatively functionally redundant signaling components, other interesting topics for future work include whether parallel pathways are calcium-dependent or-independent and whether ABA hyperaccumulation, which we observed, is required for the developmental phenotypes of CCaMK-IPD3 gain-of-function lines. Calcium is a ubiquitous secondary messenger with a vast array of functions in plant cells. How specificity can be achieved and maintained when a common signal is employed for diverse functions has been a long-standing mystery. Parallel signaling pathways may be one mechanistic explanation; oscillatory calcium signals may be another mechanism for specificity and fidelity. Advances in calcium imaging and other biosensor technologies may empower future studies to demystify how calcium signals are coded in the model moss Physcomitrium.

### Summary

In this study, we demonstrated that moss homologs of CCaMK and IPD3 have retained biochemical properties critical for functionality in legumes and are able to at least partially genetically complement cognate mutants in heterologous expression assays. Nonetheless, Physcomitrium does not appear to host canonical microbial symbionts such as mycorrhizal fungi. Synthetic activation of CCaMK or its downstream target transcriptional activator IPD3 in Physcomitrium induces ABA signaling and the constitutive formation of brood cells, which serve as asexual propagules that enable escape from abiotic stresses. The unexpected finding that protonematal nuclei exhibit spontaneous calcium spiking prompts questions about the regulation of CCaMK in Physcomitrium by calcium and provides fertile ground for future studies. Overall, our observations are consistent with a model wherein PpCCaMK-IPD3 functions to decode stress-associated calcium signatures and developmental reprogramming. Functional inquiries into CCaMK and IPD3 homologs in other early-diverging embryophytes such as the mycorrhizal host plant *Marchantia paleacea* and charophyte green algae are expected to complement these efforts and provide a fuller perspective of the evolutionary establishment of the molecular mechanisms underpinning the plant-microbe common symbiosis pathway.

### Limitations of the study

Here, we have shown that synthetic activation of CCaMK or its target transcription factor triggers ABA-associated developmental reprogramming and formation of asexual propagules termed brood cells. Notably, *ccamk* or *ipd3* knockout mutants are still able to form brood cells in response to stress or ABA treatments, which would be consistent with parallel or alternative signaling processes; future work should target these putative components (e.g., by combining loss-of-function mutations). Mechanistic insight may also be gleaned by testing whether expression of synthetically activated forms of CCaMK or IPD3 is sufficient to trigger brood cell formation in mutants defective for ABA biosynthesis (e.g., Takezawa et al., 2015). Interpretation of the constitutive brood cell formation phenotype of gain-of-function lines is complicated by overexpression driven by a strong heterologous promoter. Expression of modified CCaMK or IPD3 forms from their native locus via homologous recombination may provide clearer insight into the endogenous function of CCaMK-IPD3 in Physcomitrium. Nonetheless, it is worth noting that seminal studies of CCaMK in legumes relied on constitutive strong (rather than native) promoters (Gleason et al., 2006; Tirichine et al., 2006). Deeper understanding of the role of CCaMK-IPD3 in elicitation of brood cell development may be facilitated by inducible expression of synthetically activated forms of CCaMK or IPD3 or engineering of light-controllable CCaMK or IPD3 derivatives (Zhou et al., 2012; Kubo et al., 2013). Further investigation of processes that govern CCaMK-IPD3 activation in Physcomitrium is needed, and observations of spontaneous nuclear calcium spiking hint that CCaMK-IPD3 regulation may be more complex in Physcomitrium protonemata than in legume root cells, wherein calcium spiking has been observed specifically in response to chemical elicitors (Ehrhardt et al., 1996; Chabaud et al., 2011). Except for extrapolation from legume homologs and heterologous complementation assays in this study, nothing is presently known about endogenous calcium signatures that lead to Physcomitrium CCaMK activation. The calcium imaging tools generated here provide a valuable route for further investigation. Next steps could examine effects of stress treatment on nuclear calcium oscillations; such efforts could be bolstered by inclusion of additional GECI with different spectral properties such as XCaMP-Yellow (Inoue et al., 2019), as blue and red light have been reported to trigger calcium signals in Physcomitrium protonemata (Ermolayeva et al., 1997; Russell et al., 1998).

## STAR★Methods

### Key resources table

**Table:** 

REAGENT or RESOURCE	SOURCE	IDENTIFIER
**Bacterial and virus strains**		

*Sinorhizobium meliloti* multi reporter strain CL304	Lang et al., (2018a, 2018b)	CL304
*Agrobacterium rhizogenes* MSU440	Valdés-López et al., (2019)	MSU440
*Escherichia coli* strain Rosetta2	Sigma	Cat#71400
*Escherichia coli* strain B834-pRARE2	Gromek et al., (2013)	B834-pRARE2

**Chemicals, peptides, and recombinant proteins**		

Diammonium tartrate	Sigma	Cat#09985
Abscisic acid	Sigma	Cat#A4906
X-gluc	Gold Biotechnology	Cat#G1281C
Magenta-X-gal	Gold Biotechnology	Cat#B-378
Radiolabelled ATP (μCi [g-^32^P]ATP)	Perkin Elmer	Cat#NEG002A
Trypan blue	Gibco	Cat#15250061
Wheat Germ Agglutinin Alexa Fluor®	Invitrogen	Cat#W11261
Driselase	Sigma	Cat#D8037
Hygromycin B	Thermo Fisher	Cat#10687010
G418	Thermo Fisher	Cat#11811031
Isopropyl-b-D-1-thiogalactopyranoside	Sigma	Cat#I5502
Pepstatin A	Sigma	Cat#77170
Aprotinin	Sigma	Cat#A1153
Leupeptin	Thermo Fisher	Cat#78435
BugBuster	Novagen	Cat#70584
Universal Nuclease	Pierce	Cat#88700
Amylose Resin	New England Biolabs	Cat#E8021
bovine brain Calmodulin	EMD Millipore	Cat#20-869
NuPage LDS Sample Buffer	Thermo Fisher	Cat#NP0008
Coomassie Brilliant Blue	BIO-RAD	Cat#1610436
Storage Phosphor Screen	Amersham Biosciences	Cat#21573
Streptavidin-horseradish peroxidase conjugate	GE Healthcare Life Sciences	Cat#RPN1231
Ponceau S	Sigma	Cat#P3504
Ampicillin	Sigma	Cat#A0166
Fluorescent Brightener 28 (Calcofluor white)	Sigma	Cat#F3543
D-Mannitol	Sigma	Cat#M1902
CSM-Leu-Trp	MP Biomedical	Cat#114520012-CF
CSM-Leu-Trp-His-Ade	MP Biomedical	Cat#114540412-CF
DOB Medium	MP Biomedical	Cat#114025012-CF
FastDigest DpnI	Thermo Fisher	Cat#FD1703
EGTA	Sigma	Cat#E3889
TWEEN 20	Sigma	Cat#P1379
Quantitect Reverse Transcription Kit	Qiagen	Cat#205313
iTaq Universal SYBR Green Supermix	BIO-RAD	Cat#1725121
TURBO DNase	Thermo Fisher	Cat#AM2238
RevertAid First Strand cDNA Synthesis Kit	Thermo Fisher	Cat#K1621
RNeasy Plant Mini Kit	Qiagen	Cat#74904
In-Fusion	Takara Bio	Cat#638911
Gateway BP Enzyme	Thermo Fisher	Cat#11789020
Gateway LR Enzyme	Thermo Fisher	Cat#11791020
High gel strength agar	Sigma	Cat#A9799
Phytoblend	Caisson Labs	Cat#PTP01
Phytagel	Sigma	Cat#P8169
Low-melt agarose	Bio & SELL	Cat#BS20.46
Primestar GXL DNA Polymerase	Takara	Cat#R050
PureLink PCR Purification Kit	Thermo Fisher	Cat#K310001

**Critical commercial assays**		

Pierce BSA Protein Assay	Thermo Fisher	Cat#PI23227
ECL Protein Biotinylation Module	Cytiva	Cat#RPN2202
Phytodetek ELISA kit	Agdia	Cat#PDK 09347/0096

**Deposited data**		

LjCCaMK sequence	UniProt	A0AAR7
LjIPD3 sequence	UniProt	A9XMT3
MtCCaMK sequence	UniProt	Q6RET7
MtIPD3 sequence	UniProt	A7TUE1
*Physcomitrium patens V3.3 proteome*	Phytozome 12	Lang et al., (2018a, 2018b); https://phytozome.jgi.doe.gov/pz/portal.html
*Lotus japonicus* v3.0 protein	MG20	Tang et al., (2014); https://lotus.au.dk/blast/#database-protein
*Medicago truncatula* Mt4.0 proteome	Phytozome 12	Mun et al., (2016); https://phytozome.jgi.doe.gov/pz/portal.html
Calcium imaging (GCaMP6s)	This paper	https://osf.io/hwtzb/?view_only=7fd1c63621a840b9a3b90f74a9cb26fe
Calcium imaging (MatryoshCaMP6s)	This paper	https://osf.io/hwtzb/?view_only=7fd1c63621a840b9a3b90f74a9cb26fe

**Experimental models: Cell lines**		

*Saccharomyces cerevisiae* strain AH109	Clontech	Cat#630489

**Experimental models: Organisms/strains**		

*Physcomitrium* (formerly *Physcomitrella*) *patens*ssp. *patens*	Rensing et al., (2008)	Gransden 2004
*Medicago truncatula* Jemalong A17	Ané et al., (2004)	A17
*Rhizophagus irregularis* IRBV’95	Premier Tech	ASP-A (182744)

**Oligonucleotides**		

Provided in Table S2		

**Recombinant DNA**		

pANIC5AΔRFP:NLS-GCaMP6s	This paper	AddGene 180288
pANIC5AΔRFP:NLS-MatryoshCaMP6s	This paper	AddGene 180289
pANIC5A:CCaMK	This paper	AddGene 180290
pANIC5A:CCaMK^D^	This paper	AddGene 180291
pANIC5A:CCaMK^K^	This paper	AddGene 180292
pANIC5A:IPD3	This paper	AddGene 180293
pANIC5A:IPD3^DD^	This paper	AddGene 180294
pANIC5A:eGFP-IPD3	This paper	AddGene 180295
pANIC5A:eGFP-IPD3^DD^	This paper	AddGene 180296

**Software and algorithms**		

Genious Prime	Biomatters	2021.1
Prism	GraphPad	V.6
Molecular Dynamics ImageQuant	Bioz Stars	5.2
R	R Core Team	2014
Zen Blue	Zeiss	V2.6
Origin Pro 2020	OriginLab	2020
Helicon Focus	Helicon Soft	2012-2015
FIJI	Schindelin et al., (2012)	2017
Media Encoder	Adobe	2021

**Other**		

Particle Delivery System	BIO-RAD	PDS-1000/He
1,100 PSI Rupture Disks	BIO-RAD	Cat#1652329
DNAdel 1 micron gold particles	Seashell Technologies	Cat#s1000d
Microscope Cavity Slides	VWR	Cat#MARI1216530

### Resource availability

#### Lead Contact

Further information and requests for resources, data, and reagents should be directed to and will be fulfilled by the lead contact, Dr. Thomas Kleist (kleistt@hhu.de).

#### Materials availability

Plasmids generated in this study have been deposited to Addgene. Unique identifies are provided in the Key resources table.

### Experimental model and subject details

#### Moss culture and growth conditions

For moss growth and phenotypic assays, *Physcomitrium patens*, ecotype Gransden 2004, was used (Rensing et al*.*, 2008). Tissue was grown on BCD medium supplemented with 5 mM diammonium tartrate (BCDAT medium), pH 6.0, supplemented with 0.8% high gel strength agar (Sigma) or 1% Phytoblend (Caisson Labs). For stress assays, the medium was supplemented with (+/−)-abscisic acid (Sigma) or mannitol, as indicated. Moss cultures were grown in a growth chamber at 22°C under 50-100μmolm^−2^s^−1^ light with a 16-hour photoperiod. *Medicago truncatula* mutant-rescue assays were performed as previously described (Delaux et al., 2015).

#### Medicago mutant rescue and symbiotic phenotype screening

*Medicago truncatula* mutant-rescue assays were performed as previously described (Delaux et al*.*, 2015) but were instead inoculated with the *S. meliloti* multi reporter strain CL304 (Lang et al., 2018a, 2018b) for *ipd3-1* rescues. Following nodulation, we stained the roots with X-Guc and magenta-X-gal following procedures in Schiessl et al. (2019) without tissue prefixation. *pENOD11::GUS* plants expressing different variations of *CCaMK* were stained 3 weeks post-transformation with GUS staining solution as done in Radhakrishnan et al. (2020). As controls, *pENOD11::GUS* plants with transgenic roots expressing an EV control vector were either subject to a 24-hour treatment of 10^−8^ M LCOs from *S. meliloti* (GM16390) or left untreated before GUS staining.

#### Co-culture of Physcomitrium and *Rhizophagus irregularis*

Three-week-old gametophores were collected from cellophane-overlaid Knop agar plates. Gametophores were transferred to half-strength Knop semi-liquid medium containing 0.15% Phytagel (Sigma) in 24-well plates. In each well, 2 mL of semi-liquid Knop medium was poured, and approximately 50 spores of *R. irregularis* IRBV′95 were added. One gametophore was placed gently over the medium such that the rhizoids were immersed within the semi-liquid medium. This experimental setup was incubated at 25 °C with a light intensity of 55μmolm^−2^s^−1^, with a 16 h photoperiod, for six months. Starting from one month after co-culturing, AMF colonization was analyzed every week for up to six months using bright-field or confocal microscopy. Trypan blue staining of *R. irregularis* was performed as described (Koske and Gemma, 1989). For confocal microscopy, the fungal hyphae were stained using Wheat Germ Agglutinin Alexa Fluor® 488 (Excitation: 488 nm; Emission: 520 nm), and the rhizoids, and gametophyte tissues were observed by chlorophyll autofluorescence (Excitation: 488 nm, Emission: 670 nm).

### Method details

#### Bioinformatic analyses

To identify CCaMK and IPD3 homologs encoded in the Physcomitrium genome, the full-length protein sequences of CCaMK and IPD3 from *Lotus japonicus* (LjCCaMK, UniProt: A0AAR7, Lotus Base: Lj3g3v1739280; LjIPD3, UniProt: A9XMT3, Lotus Base: Lj2g3v1549600) and *Medicago truncatula* (MtCCaMK, UniProt: Q6RET7, Phytozome: Medtr8g043970; MtIPD3, UniProt: A7TUE1, Phytozome: Medtr5g026850) were retrieved from UniProt and used as BLASTp queries against the predicted *Physcomitrium patens* version 3.3 predicted proteome (Lang et al., 2018b) using Phytozome 12 (https://phytozome.jgi.doe.gov/pz/portal.html). The BLOSUM62 scoring matrix was used. E-value thresholds were set to −1 for CCaMK searches and 1 × 10^4^ for IPD3 searches. Other parameters followed default settings. Data were downloaded and analyzed from December 11 to 17, 2019. The top five hits were used as queries for reciprocal BLASTp searches against either the *Lotus japonicus* MG20 v3.0 protein database (https://lotus.au.dk/blast/#database-protein) using default settings or the *Medicago truncatula* Mt4.0 predicted proteome, accessed through Phytozome 12 and performed using default settings (Tang et al., 2014; Mun et al., 2016). For each reciprocal BLASTp search, the top hit was displayed. Multiple sequence alignments were made using MUSCLE (Edgar, 2004) version 3.8.425 plugin for Geneious Prime under default settings and were annotated manually or using the InterProScan feature in Geneious Prime (Biomatters).Accession numbers for putative orthologs referred to in Figure 1A can be found in Table S1.

#### Molecular cloning and plasmid construction

DNA and RNA were extracted from protonemal tissue by chloroform phase separation and cetrimonium bromide (CTAB) buffer as previously described (Chang et al., 1993; Kleist et al., 2014). The Quantitect (Qiagen) reverse transcription kit was used to synthesize cDNA for cloning and qPCR. PCR reactions were performed using Phusion (Thermo Fisher) or Primestar GXL DNA Polymerase (Clontech). The sequences of oligonucleotide primers used in this study are given in Table S3. Single- and multi-site Gateway (Thermo Fisher) cloning reactions were performed per the manufacturer’s recommendations. The coding sequences (CDSs) of *PpCCaMK, PpCCaMKb,* and *PpIPD3* were cloned into pDONR/Zeo and modified, as described, by site-directed mutagenesis using whole-plasmid amplification with anticomplementary primers followed by digestion with FastDigest *Dpn*I (Thermo Fisher). The coding sequences of *PpCCaMK*, *PpCCaMKb*, and *PpIPD3* were subcloned into pGAD-GH-GW or pGBT9-GW for yeast two-hybrid analysis. Assembly PCR was used to attach an eGFP tag and a polyglycine linker to the N-terminus of IPD3 or IPD3^DD^. The vectors containing NLS-GCaMP6s or NLS-MatryoshCaMP6s (Ast et al., 2017) driven by *Zea mays UBIQUITIN1* promoter were cloned by Gateway LR reaction into a modified pANIC5a vector with the region encoding *Porites porites* RFP deleted by PCR followed by In-Fusion reaction (Takara).

For protein expression and purification, the coding sequence of maltose-binding protein (MBP) was fused to the N-terminus of the native coding sequences of PpCCaMKa, PpCCaMKb, CrCDPK1, and MtCCaMK. Protein expression vectors were cloned by Gateway (Invitrogen) subcloning of pENTR-D/TOPO donor clones into the pVP16 destination clone via LR recombination (Invitrogen). MBP-PpCCaMKa, MBP-PpCCaMKb, and MBP-CrCDPK1 fusion constructs were transformed into *E. coli* strain Rosetta2 whereas the MBP-MtCCaMK fusion construct was transformed into *E. coli* strain B834-pRARE2 for protein expression.

Native or modified CDSs were subcloned into pANIC5A for plant expression (Mann et al., 2012), pVP16 for N-terminal fusion to maltose-binding protein (MBP), pK7FWG2 for expression in *Medicago truncatula* roots, and pGBT9-BS-GW (*PpCCaMK* and *PpCCaMKb*) or pGAD-GH-GW (*PpIPD3*) for yeast two-hybrid assays. Multisite Gateway recombination was used to generate the deletion construct for *IPD3*. Approximately one kilobase region located at the N-terminal or C-terminal end of the gene was cloned into pENTRY attB1-attB4 or attB3-attB2 vectors. *Porites* sp. red fluorescent protein (RFP), driven by a *Panicum virgatum UBIQUITIN* promoter (Mann et al., 2012), was cloned into a pENTRY attB4r-attB5r vector. An antibiotic resistance construct containing the *NPTII* gene driven by a cauliflower mosaic virus 35S promoter was cloned into a pENTRY attB5-attB3r vector. The four fragments were assembled by LR reaction using LR clonase plus enzyme (Thermo Fisher) using a Gateway-compatible destination vector with attL1 and attL2 sites. After sequence confirmation, the linear deletion construct was amplified by PCR using Primestar GXL DNA Polymerase (Takara). The PCR product was purified and concentrated to 1 μg/μL using the PureLink PCR purification kit (Invitrogen).

For rescue of *ipd3-1*, we amplified the 1,233 bp region upstream of the *MtIPD3* start codon (*MtIPD3* promoter) and 442 bp region downstream of the *MtIPD3* stop codon (*MtIPD3* terminator) from Medicago genomic DNA and cloned them into Golden-Gate level 0 acceptor plasmids (Horváth et al., 2011). For *ipd3-1* rescue experiments, the *MtIPD3* or *PpIPD3* coding sequences were then cloned into level 1 Golden-Gate cloning vectors along with the *MtIPD3* promoter and terminator sequences. For gain-of-function *CCaMK* experiments, the coding sequences of *MtCCaMK*, *MtCCaMK*^*K*^, *PpCCaMK*, and *PpCCaMK*^*K*^ were combined with a 35S promoter and terminator into Golden-Gate level 1 vectors. The *IPD3* and *CCaMK* Golden-Gate level 1 variants were then cloned into separate Golden-Gate level 2 vectors, containing a tdTomato fluorescent reporter. All Golden-Gate cloning reactions were performed following the procedures in Binder et al., (2014). Briefly, type IIs restriction enzymes were used to produce linear fragments for ligation.

#### Biochemistry and yeast two-hybrid assays

##### Heterologous protein expression and purification

Single bacterial colonies were grown overnight at 37°C shaking at 225 rpm in 10 mL of LB broth supplemented with 50 μg/mL ampicillin. 2 mL of overnight culture were inoculated into 2 L flasks containing 200 mL of LB broth supplemented with 50 μg/mL ampicillin and were grown at 37°C shaking at 225 rpm until cultures reached an optical density of OD_600_ = 0.5 at which time protein expression was induced and cultures were moved to 25°Cat 225 rpm for 4 h. Protein expression was induced by the addition of isopropyl-b-D-1-thiogalactopyranoside to a final concentration of 0.5 mM. After 4 h of protein expression, cultures were chilled on ice for 10 min and fully pelleted by centrifugation at 10,000 rcf for 10 min at 4°C. Bacterial pellets were fully resuspended in 9 mL of chilled protein extraction buffer (20 mM HEPES pH 7.4, 300 mM NaCl, 5 mM MgCl_2_, 10% glycerol, 2 mM mercaptoethanol). Protein extraction buffer was supplemented before use with 1 mM phenylmethylsulfonyl fluoride, 50 μg/mL pepstatin A, 50 μg/mL aprotinin, and 50 μg/mL leupeptin to inhibit proteolysis. 1 mL of 10x BugBuster (Novagen) reagent was added to bacterial pellet suspensions and mixed by gentle inversion. 1 μL of Universal Nuclease (Pierce) was added and mixed by gentle inversion to reduce cell lysate viscosity. Protein extractions were then incubated at room temperature for 30 min with gentle agitation at 30 rpm. Maltose-binding protein (MBP) fused to PpCCaMK, PpCCaMKb, and CrCDPK1 fusion constructs were transformed into *E. coli* strain Rosetta2, whereas the MBP-MtCCaMK fusion construct was transformed into *E. coli* strain B834-pRARE2 for protein expression. Protein expression and purification were carried out as previously described (Delaux et al., 2015) and described below. Following protein extraction, cell lysates were centrifuged at 15,000 rcf for 30 min at 4°C to pellet cell debris. Clarified lysates were transferred to clean tubes and chilled on ice while protein purification resin was prepared. Per each 10 mL lysate, 1 mL of Amylose Resin (New England Biolabs) affinity matrix was pre-washed twice in 10 mL of phosphate-buffered saline (PBS). Lysates were then transferred to washed amylose resin and incubated at 4°C with slow rotation for 4 h. After cold incubation, amylose resin was pelleted at 1,500 rcf at 4 °C for 3 min and cell lysates were discarded. The remaining resin was washed three times with 10 mL PBS using the same cold incubation and centrifugation conditions. After washing, bound protein was eluted from the amylose resin by resuspension in PBS supplemented with 50 mM maltose followed by incubation on ice for 10 min with occasional swirling. Amylose resin was pelleted at 1,500 rcf at 4 °C for 3 min and supernatant protein elution was transferred to 1.5 mL tubes, flash frozen in liquid nitrogen, and stored at −80 °C until assay use.

##### Kinase activity and calmodulin-binding assays

Kinase assays were performed as previously described (Miller et al., 2013) with slight modifications. Prior to assay, purified protein concentration was assessed by Pierce BCA Protein Assay (Pierce). 1 μg of MBP-PpCCaMK or MBP-PpCCaMKb protein was incubated in ATP-containing buffer spiked with radiolabeled ATP (50 mM HEPES, pH 7.5, 1 mM 1,4-dithiothreitol, 200 μM ATP, 5 μCi [ɣ-32P]ATP (Perkin Elmer), and 10 mM MgCl_2_). 20 μL reactions were incubated at 30°C for 5 min with 0.2 mM CaCl_2_ (+Ca^2+^) or 2.5 mM EGTA (+EGTA). Where stated, 0.5 μM bovine Calmodulin (Millipore) was added. Reactions were terminated by addition of 7 μL 4x NuPage LDS Sample Buffer (Thermo Fisher) and incubation at 95 °C for 5 min. Subsequently, samples were separated on 10% SDS-PAGE gels before imaging. Proteins were stained and fixed by incubation in a solution of 50% methanol, 10% glacial acetic acid, and 1% (w/v) Coomassie Brilliant Blue (BIO-RAD) on a shaking platform at ambient temperature at 30 rpm for 4 h. Stained, fixed gels were then destained in deionized water for 4 h. Gels were then exposed to a Storage Phosphor Screen (Amersham Biosciences) for 1 h immediately prior to imaging. Radioactivity was quantified by Molecular Dynamics Storm® 860 phosphorimager, and data were analyzed using the Molecular Dynamics ImageQuant® software. Calmodulin-binding assays using recombinant CCaMKs were performed similarly to a prior study (Routray et al., 2013) with slight modifications. Bovine brain calmodulin (EMD Millipore) was biotinylated using the ECL Protein Biotinylation Module (GE Healthcare Life Sciences). Recombinant CCaMKs were separated on a 10% SDS-PAGE gel and then immobilized by transfer to a polyvinylidene difluoride (PVDF) membrane. PVDF membranes were then incubated in Tris-buffered saline, 0.1% TWEEN 20 (TBS-T) supplemented with 1 mM CaCl_2_ and 3 μg/mL biotinylated calmodulin for 1 h on a shaking platform at 4°C at 30 rpm. Subsequently, the membrane was incubated in TBS-T supplemented with 1:6,000 streptavidin-horseradish peroxidase conjugate (GE Healthcare Life Sciences) for 1 h. Bound calmodulin was detected using ECL Prime (GE Healthcare Life Sciences) and imaged using a ImageQuant LAS 500 (GE Healthcare Life Sciences) chemiluminescence imager. After chemiluminescent assay, total protein content was determined by Ponceau S staining. PVDF membranes were washed in TBS-T and then stained in 0.1% Ponseau S, 10% acetic acid for 30 min followed by three brief destainings in deionized water. Ponceau S-stained protein gels were imaged using a Nikon D600 DSLR camera.

##### Extraction and quantification of ABA

Abscisic acid (ABA) extraction and quantitation was done was previously described with slight modifications (Ondzighi-Assoume et al., 2016). Extraction from moss tissue was performed using methanol. For each sample, 100 mg of fresh tissue was collected, flash frozen in liquid nitrogen, and lyophilized. After lyophilization, three sterile 3 mm glass beads were added to the dried samples and the samples were macerated at max speed in a bead-beater for 3 min. 1 mL of chilled methanol supplemented with 2.5 mM citric acid monohydrate and 0.5 mM 2,6-di-ter-butyl-4-methly-phenol was added to the sample tubes and the samples were incubated for 16 h on a shaking platform at 4°C at 30 rpm. After extraction, sample tubes were centrifuged at 5,000 rcf for 10 min at 4°C, and the supernatant methanol extractions were transferred to clean tubes. Sterile deionized water was added to each extraction to adjust to 70% methanol. To remove chlorophyll and other assay-inhibiting compounds, the adjusted extractions were each passed through a C18 Sep-Pak cartridge (Waters) that was pre-equilibrated with fresh 70% methanol. The resulting eluates were centrifuged and dried in a speed vac at ambient temperature for 16 h. The resulting dried eluates were resuspended in TBS prior to 40-fold dilution with TBS and subsequent assay by colorimetric Phytodetek ELISA kit (Agdia) using the manufacturer’s standard protocol. ELISA plate results were quantified using a Tecan Infinite M1000 Pro microplate reader.

##### Yeast two-hybrid assays

Expression vectors containing *PpCCaMK*, *PpCCaMKb*, and *PpIPD3* were co-transformed into *Saccharomyces cerevisiae* strain AH109 using the lithium acetate method established by Gietz and Schiestl (2007). Interactions were assayed by growth on solid complete synthetic media lacking leucine and tryptophan (CSM-Leu-Trp) or lacking leucine, tryptophan, histidine, and adenine (CSM-Leu,-Trp,-His,-Ade, MP Biomedicals) at 30°C, as previously described (Kleist et al., 2014).

#### Plant transformation

Biolistic transformation of Physcomitrium protonema was carried out as previously described (Kleist et al., 2017). Briefly, purified plasmid DNA was precipitated onto 1 μm spherical gold particles (Seashell Technologies), following manufacturer recommendations. 1,110 pounds per square inch (PSI) rupture disks were used (Bio-Rad). Particles were delivered to protonemal cultures grown on cellophane-overlaid solid BCDAT medium; parameters for bombardment were identical to step-by-step description provided in Kleist et al. (2017). Bombarded cultures were moved onto BCDAT medium supplemented with Hygromycin B (Thermo Fisher) 50 μg/mL concentration. The pANIC5A vector, which contains a free *Porites porites* red fluorescent protein (RFP) driven by a separate promoter (Mann et al., 2012), was used for each construct. Colonies that survived antibiotic selection were embedded in solid BCDAT medium supplemented with 50 μg/mL Hygromycin B (Thermo Fisher), and lines that showed RFP fluorescence were selected for characterization. Transgenic lines were maintained by transfer to fresh BCDAT medium supplemented with 50 μg/mL Hygromycin B (Thermo Fisher). Phenotypic analyses were performed using cultures grown on solid cellophane-overlaid BCDAT medium without antibiotic for 2-4 weeks. Retention of transgenes was verified by checking RFP fluorescence. After 4 weeks, a subset of transformants was transferred from antibiotic-free media back to media with 50 μg/mL Hygromycin B (Thermo Fisher) and were found to have retained antibiotic resistance, which is consistent with the interpretation that these lines were stably transformed. Multiple independent transformants for each gain-of-function construct described have been maintained in multiple laboratories by serial subculture for more than 4 years, which also suggests stability of transformation events. Nonetheless, it is unclear whether or not transgenes were chromosomally integrated, which we do not consider to be essential to the conclusions drawn here despite being theoretically desirable.

Protoplasts were obtained from one-week-old protonemal cultures using 2% Driselase (Sigma, D8037) in an 8% mannitol solution for cell wall digestion. Protoplast transformations to produce *ccamk* or *ipd3* deletion mutants were performed as previously with minor modifications (Hohe et al., 2004). Briefly, an 8% mannitol solution was made using 1/10 BCDAT medium set to pH 5.8 to improve protoplast survival. Antibiotic selection was performed using 50 μg/mL Hygromycin B or 40 μg/mL G418. For *ipd3* deletion lines, stable transformants were identified by RFP fluorescence using a Zeiss Lumar epifluorescence stereoscope. *Medicago truncatula* root transformations were carried out using *Agrobacterium rhizogenes* strain MSU440, harboring either the pK7FWG2 binary vector (Delaux et al.*,* 2015) or Golden-Gate Level 2 binary vector pAGM4673 for *ipd3-1* rescue assays.

#### Quantitative RT-PCR

Total mRNA was extracted from abscisic acid (ABA)-treated and untreated wild-type samples, as well as from gain-of-function lines. 3 μg RNA samples were reverse transcribed using the Quantitect Reverse Transcription kit (Qiagen), per manufacturer’s recommendations, and the resulting 60 μL cDNA samples were diluted by the addition of 90 μL nuclease-free water. The iTaq Universal SYBR Green Supermix (Bio-Rad) was used for qPCR reactions following the manufacturer's recommendations, and reactions were run on a DNA Engine Opticon™ continuous fluorescence detector. Ubiquitin-conjugating enzyme E2 (Pp1s34_302V6) was used throughout as a reference gene (Le Bail et al., 2013). After baseline subtraction, results were analyzed using the ΔΔC(t) method, wherein fold change is taken as 2ˆ[(reference gene – query gene)_transgenic/treated line_– (reference gene – query gene)_wildtype/untreated line_]. For each line, three to four biological replicates were used, with a minimum of two technical replicates (i.e., each qPCR run was performed multiple times). For transgene quantification, 100 mg fresh Physcomitrium tissue of was harvested into liquid nitrogen and the RNA was extracted using RNeasy Plant Mini Kit (QIAGEN). DNA was eliminated from the RNA samples by TURBO DNase (Thermo Fisher Scientific) and cDNA was synthesized using RevertAid First Strand cDNA Synthesis Kit (Thermo Fisher Scientific) from 1ug of RNA. Primer pairs (CCaMK-qF/R and IPD3qF/R) are provide in Table S2. Quantitative PCR was performed using CFX96 Touch Real-Time PCR Detection System (BioRad) and SsoAdvanced Universal SYBR Green Supermix (BioRad).

#### Microscopy, photography, and image processing

Differential interference contrast (DIC) micrographs were acquired using a Zeiss AxioImager M1 microscope with a 40X, 1.4 numerical aperture (NA) or 100X, 1.3 NA objective and a QImaging five MPix Micro-Publisher color camera or an Olympus BX60 microscope using a 20X, 0.5 NA and an Olympus DP 72 color camera. Macrophotography images were acquired using a Canon EOS 6D 20.2 Megapixel CMOS Digital SLR camera equipped with a Canon MP-E 65 mm f/2.8 1-5x macro lens and Canon MT-24EX macro twin-light flash. Manually acquired z-stacks were focus-stacked using Helicon Focus (HeliconSoft). A Zeiss AxioZoom.V16 equipped with Plan NeoFluar Y 1x/0.25 NA objective and Axiocam 305 color camera (Zeiss) was used for cell dimension measurements.

Confocal fluorescence microscopy was performed using a Leica TCS SP8 equipped with a resonant scanner and white light laser (WLL). Scan speed was set to 8000 Hertz. A 20x/0.70 NA multi-immersion objective was used with glycerin. Fluorescence images were collected in three-step sequences on HyD SMD detectors. RFP was detected at 575-625 nanometers (nm), and chlorophyll was detected from 650-750 nm under WLL excitation at 561 nm with a notch filter 488/561/633. eGFP was detected at 500-550 nm under WLL excitation at 488 nm with notch filter 488, and transmitted light was detected with a PMT. Calcofluor white was detected at 415-465 nm under 405 nm excitation. Samples were stained with 10 mg/mL calcofluor white/fluorescent brightener 28 (Sigma, F3543) for 30 min with gentle agitation and rinsed twice with sterile water before imaging. Subcellular localization images shown are average z-stack projections and were prepared using FIJI (Schindelin et al., 2012).

#### Calcium imaging

A Zeiss AxioZoom V16. zoom microscope equipped with a metal halide illuminator (HXP 200C, Zeiss) set to illumination setting 1, sCMOS camera (ORCA-Flash 4.0, Hamamatsu), and 2.3x objective lens (Plan-NEOFLUAR Z 2.3x, NA 0.5) to monitor calcium dynamics in *Physcomitrium patens*. Zoom magnification was set to 4.3-8x for acquisitions. Protonematal cells expressing NLS-GCaMP6s or NLS-MatryoshCaMP6s were mounted on 8 mm diameter cavity slides (VWR) using BCDAT medium either in liquid form or solidifed with 1% (w/v) low-melt agarose (Bio & SELL) for stabilization. No obvious differences were observed between samples imaged in liquid versus solid medium. Acquisition times varied between 30 min and 1 h with an exposure time of 15-20 ms and an interval time of 15-20 s with the bandpass excitation filter (λ_ex_) at 470/40 nm, bandpass emission filter (λ_em_) at 525/50 nm, with a longpass beam splitter at 495 nm for GFP and *λ*_ex_ = 436/20 nm, *λ*_ex_ = 575/40 nm with a longpass beam splitter 550 nm for LSSmOrange acquisition. Z-Stack acquisitions were performed to maximize focal depth. Maximum z-stack projections were performed using FIJI (Schindelin et al., 2012). Pseudo-color 16-bit images were converted to RGB format, and movies were rendered in FIJI. Movies were down-sampled and converted to mp4 format in Adobe Media Encoder. 16-bit maximum z-stack datasets were converted to 8-bit in FIJI and deposited at the Open Science Foundation, URL: https://osf.io/hwtzb/?view_only=7fd1c63621a840b9a3b90f74a9cb26fe.

### Quantification and statistical analyses

#### Measurement of cell dimensions

Physcomitrium samples were taken from edges of approximately month-old cultures grown under standard conditions, as described above. Three independently transformed lines were analyzed per construct. Measurements were performed manually in Zen Blue software version 2.6 (Zeiss). Graphs were made using Origin Pro 2020.

#### Statistical analyses

For quantitative measurements of ABA content, seven biological replicates were analyzed for wild-type, and three biological replicates of three independently transformed lines were analyzed for IPD3^DD^. Every biological replicate was tested in triplicate for the ABA ELISA. Data were analyzed and tested using a Student’s T-Test using the R statistical programming language (R Core Team, 2014). For statistical analyses of RT-qPCR data, samples were compared via one-way ANOVA analysis using R (R Core Team, 2014). Levene’s Test confirmed equality of variance for both sets of data (Levene, 1960). The Tukey honest significant difference (HSD) was used for posthoc analysis of ANOVA results (Tukey, 1949), and the p values that were reported were calculated using this method. For *ipd3-1* rescue experiments, each treatment was compared for differences in total nodule number per root, the number of colonized nodules per root, and the number of nodules containing rhizobia expressing nifH per root using R (R Core Team, 2014). The sample size per treatment varied from 16 roots to 44 roots. Levene’s test determined equal variance for total nodule number between samples, but not for either number of colonized nodules or number of nifH expressing nodules (Levene, 1960). We then performed an ANOVA for the total number of nodules and Kruskal-Wallace tests for both the number of colonized nodules and nifH expressing nodules. Dunn's test with a Benjamini-Hockberg p-value adjustment was performed for posthoc analysis of the Kruskal-Wallace results. For statistical analyses of moss cell dimension data, Welch’s t-test and Mann-Whitney U-test were performed using Origin Pro 2020. The same data were analyzed by one-way Anova (Tukey's multiple comparisons test) using Prism: GraphPad V.6. Blinding strategies were not used for any of the described experiments. Exact n values and definition are provided in each legend. Symbols used in statistical graphs are defined by legend. Sample size was not pre-determined.

## Data Availability

• Calcium imaging data have been deposited at the Open Science Foundation and are publicly available as of the date of publication. DOIs are listed in the key resources table. • This paper does not report any original code. • Any additional information required to reanalyze the data reported in this paper is available from the lead contact upon request.

## References

[bib121] Altschul S.F., Gish W., Miller W., Myers E.W., Lipman D.J. (1990). Basic local alignment search tool. J. Mol. Biol..

[bib1] Ané J.-M., Kiss G.B., Riely B.K., Penmetsa R.V., Oldroyd G.E.D., Ayax C., Lévy J., Debellé F., Baek J.-M., Kalo P. (2004). *Medicago truncatula DMI1* required for bacterial and fungal symbioses in legumes. Science.

[bib2] Ast C., Foret J., Oltrogge L.M., De Michele R., Kleist T.J., Ho C.H., Frommer W.B. (2017). Ratiometric Matryoshka biosensors from a nested cassette of green-and orange-emitting fluorescent proteins. Nat. Commun..

[bib3] Le Bail A., Scholz S., Kost B. (2013). Evaluation of reference genes for RT qPCR analyses of structure-specific and hormone regulated gene expression in *Physcomitrella patens* gametophytes. PLoS One.

[bib122] Banba M., Gutjahr C., Miyao A., Hirochika H., Paszkowski U., Kouchi H., Imaizumi-Anraku H. (2008). Divergence of evolutionary ways among common *sym* genes: CASTOR and CCaMK show functional conservation between two symbiosis systems and constitute the root of a common signaling pathway. Plant Cell Physiol.

[bib4] Bascom C.S., Winship L.J., Bezanilla M. (2018). Simultaneous imaging and functional studies reveal a tight correlation between calcium and actin networks. Proc. Natl. Acad. Sci. U S A.

[bib5] Binder A., Lambert J., Morbitzer R., Popp C., Ott T., Lahaye T., Parniske M. (2014). A modular plasmid assembly kit for multigene expression, gene silencing and silencing rescue in plants. PLoS One.

[bib6] Bonfante P., Genre A. (2008). Plants and arbuscular mycorrhizal fungi: an evolutionary-developmental perspective. Trends Plant Sci..

[bib7] Bopp M. (2000). Progress in Botany.

[bib8] Bossuyt J., Bers D.M. (2013). Visualizing CaMKII and CaM activity: a paradigm of compartmentalized signaling. J. Mol. Med..

[bib9] Broghammer A., Krusell L., Blaise M., Sauer J., Sullivan J.T., Maolanon N., Vinther M., Lorentzen A., Madsen E.B., Jensen K.J., Roepstorff P. (2012). Legume receptors perceive the rhizobial lipochitin oligosaccharide signal molecules by direct binding. Proc. Natl. Acad. Sci.U S A.

[bib10] Bressendorff S., Azevedo R., Kenchappa C.S., Ponce de Leon I., Olsen J.V., Rasmussen M.W., Erbs G., Newman M.-A., Petersen M., Mundy J. (2016). An innate immunity pathway in the moss *Physcomitrella patens*. Plant Cell.

[bib11] Buendia L., Wang T., Girardin A., Lefebvre B. (2015). The LysM receptor-like kinase SlLYK10 regulates the arbuscular mycorrhizal symbiosis in tomato. New Phytol..

[bib12] Capoen W., Sun J., Wysham D., Otegui M.S., Venkateshwaran M., Hirsch S., Miwa H., Downie J.A., Morris R.J., Ané J.-M. (2011). Nuclear membranes control symbiotic calcium signaling of legumes. Proc. Natl. Acad. Sci. U S A.

[bib13] Carleton T.J., Read D.J. (1991). Ectomycorrhizas and nutrient transfer in conifer–feather moss ecosystems. Can. J. Bot..

[bib14] Chabaud M., Genre A., Sieberer B.J., Faccio A., Fournier J., Novero M., Barker D.G., Bonfante P. (2011). Arbuscular mycorrhizal hyphopodia and germinated spore exudates trigger Ca^2+^ spiking in the legume and nonlegume root epidermis. New Phytol..

[bib15] Chang S., Puryear J., Cairney J. (1993). A simple and efficient method for isolating RNA from pine trees. Plant Mol. Biol. Rep..

[bib16] Chen C., Ané J., Zhu H. (2008). OsIPD3, an ortholog of the *Medicago truncatula* DMI3 interacting protein IPD3, is required for mycorrhizal symbiosis in rice. New Phytol..

[bib17] Chen T.W., Wardill T.J., Sun Y., Pulver S.R., Renninger S.L., Baohan A., Schreiter E.R., Kerr R.A., Orger M.B., Jayaraman V., Looger L.L. (2013). Ultrasensitive fluorescent proteins for imaging neuronal activity. Nature.

[bib18] Correns C. (1899).

[bib19] Davey M.L., Currah R.S. (2006). Interactions between mosses (Bryophyta) and fungi. Botany.

[bib20] Delaux P.-M. (2017). Comparative phylogenomics of symbiotic associations. New Phytol..

[bib21] Delaux P.-M., Séjalon-Delmas N., Bécard G., Ané J.-M. (2013). Evolution of the plant–microbe symbiotic ‘toolkit.’. Trends Plant Sci..

[bib22] Delaux P.-M., Radhakrishnan G.V., Jayaraman D., Cheema J., Malbreil M., Volkening J.D., Sekimoto H., Nishiyama T., Melkonian M., Pokorny L. (2015). Algal ancestor of land plants was preadapted for symbiosis. Proc. Natl. Acad. Sci. U S A.

[bib23] Delaux P.-M., Varala K., Edger P.P., Coruzzi G.M., Pires J.C., Ané J.M. (2014). Comparative phylogenomics uncovers the impact of symbiotic associations on host genome evolution. PLoS Genet..

[bib24] Doyle J.J. (2011). Phylogenetic perspectives on the origins of nodulation Mol. Plant Microbe Interact..

[bib25] Duckett J.G., Ligrone R. (1992). A survey of diaspore liberation mechanisms and germination patterns in mosses. J. Bryol..

[bib26] Fernandez-Pozo N., Haas F.B., Meyberg R., Ullrich K.K., Hiss M., Perroud P.F., Hanke S., Kratz V., Powell A.F., Vesty E.F., Daum C.G. (2020). PEATmoss (*Physcomitrella* Expression Atlas Tool): a unified gene expression atlas for the model plant *Physcomitrella patens*. Plant J..

[bib27] Edgar R.C. (2004). MUSCLE: a multiple sequence alignment method with reduced time and space complexity. BMC Bioinform..

[bib28] Ehrhardt D.W., Wais R., Long S.R. (1996). Calcium spiking in plant root hairs responding to Rhizobium nodulation signals. Cell.

[bib29] Ermolayeva E., Sanders D., Johannes E. (1997). Ionic mechanism and role of phytochrome-mediated membrane depolarisation in caulonemal side branch initial formation in the moss *Physcomitrella patens*. Planta.

[bib30] Fliegmann J., Canova S., Lachaud C., Uhlenbroich S., Gasciolli V., Pichereaux C., Rossignol M., Rosenberg C., Cumener M., Pitorre D. (2013). Lipo-chitooligosaccharidic symbiotic signals are recognized by LysM receptor-like kinase LYR3 in the legume *Medicago truncatula*. ACS Chem. Biol..

[bib31] Frank M.H., Scanlon M.J. (2015). Cell-specific transcriptomic analyses of three-dimensional shoot development in the moss *Physcomitrella patens*. Plant J..

[bib32] Galotto G., Abreu I., Sherman C., Liu B., Gonzalez-Guerrero M., Vidali L. (2020). Chitin triggers calcium-mediated immune response in the plant model Physcomitrella patens. Mol. Plant Microbe Interact..

[bib33] Garcia K., Delaux P., Cope K.R., Ané J. (2015). Molecular signals required for the establishment and maintenance of ectomycorrhizal symbioses. New Phytol..

[bib34] Garcia K., Chasman D., Roy S., Ané J.-M. (2017). Physiological responses and gene co-expression network of mycorrhizal roots under K^+^ deprivation. Plant Physiol..

[bib35] Genre A., Chabaud M., Faccio A., Barker D.G., Bonfante P. (2008). Prepenetration apparatus assembly precedes and predicts the colonization patterns of arbuscular mycorrhizal fungi within the root cortex of both *Medicago truncatula* and *Daucus carota*. Plant Cell.

[bib36] Gietz R.D., Schiestl R.H. (2007). Frozen competent yeast cells that can be transformed with high efficiency using the LiAc/SS carrier DNA/PEG method. Nat. Protoc..

[bib37] Gleason C., Chaudhuri S., Yang T., Munoz A., Poovaiah B.W., Oldroyd G.E.D. (2006). Nodulation independent of rhizobia induced by a calcium-activated kinase lacking autoinhibition. Nature.

[bib38] Gobbato E., Marsh J.F., Vernié T., Wang E., Maillet F., Kim J., Miller J.B., Sun J., Bano S.A., Ratet P. (2012). A GRAS-type transcription factor with a specific function in mycorrhizal signaling. Curr. Biol..

[bib39] Gromek K.A., Meddaugh H.R., Wrobel R.L., Suchy F.P., Bingman C.A., Primm J.G., Fox B.G. (2013). Improved expression and purification of sigma 1 receptor fused to maltose binding protein by alteration of linker sequence. Protein Expr. Purif..

[bib40] Hohe A., Egener T., Lucht J.M., Holtorf H., Reinhard C., Schween G., Reski R. (2004). An improved and highly standardised transformation procedure allows efficient production of single and multiple targeted gene-knockouts in a moss, *Physcomitrella patens*. Curr. Genet..

[bib41] Horváth B., Yeun L.H., Domonkos Á., Halász G., Gobbato E., Ayaydin F., Miró K., Hirsch S., Sun J., Tadege M. (2011). *Medicago truncatula IPD3* is a member of the common symbiotic signaling pathway required for rhizobial and mycorrhizal symbioses. Mol. Plant Microbe Interact..

[bib42] Huang S., Waadt R., Nuhkat M., Kollist H., Hedrich R., Roelfsema M.R.G. (2019). Calcium signals in guard cells enhance the efficiency by which abscisic acid triggers stomatal closure. New Phytol..

[bib43] Inoue M., Takeuchi A., Manita S., Horigane S.I., Sakamoto M., Kawakami R., Yamaguchi K., Otomo K., Yokoyama H., Kim R., Yokoyama T. (2019). Rational engineering of XCaMPs, a multicolor GECI suite for in vivo imaging of complex brain circuit dynamics. Cell.

[bib123] Jin Y., Chen Z., Yang J., Mysore K.S., Wen J., Huang J., Yu N., Wang E. (2018). IPD3 and IPD3L function redundantly in rhizobial and mycorrhizal symbioses. Front Plant Sci.

[bib44] Kamel L., Keller-Pearson M., Roux C., Ané J.-M. (2016). Biology and evolution of arbuscular mycorrhizal symbiosis in the light of genomics. New Phytol..

[bib45] Kleist T.J., Spencley A.L., Luan S. (2014). Comparative phylogenomics of the CBL-CIPK calcium-decoding network in the moss *Physcomitrella, Arabidopsis*, and other green lineages. Front. Plant Sci..

[bib46] Kleist T.J., Cartwright H.N., Perera A.M., Christianson M.L., Lemaux P.G., Luan S. (2017). Genetically encoded calcium indicators for fluorescence imaging in the moss *Physcomitrella* : GCaMP3 provides a bright new look. Plant Biotechnol. J..

[bib47] Komatsu K., Suzuki N., Kuwamura M., Nishikawa Y., Nakatani M., Ohtawa H., Takezawa D., Seki M., Tanaka M., Taji T. (2013). Group A PP2Cs evolved in land plants as key regulators of intrinsic desiccation tolerance. Nat. Commun..

[bib48] Koske R.E., Gemma J.N. (1989). A modified procedure for staining roots to detect VA mycorrhizas. Mycol. Res..

[bib49] Kosuta S., Hazledine S., Sun J., Miwa H., Morris R.J., Downie J.A., Oldroyd G.E. (2008). Differential and chaotic calcium signatures in the symbiosis signaling pathway of legumes. Proc. Natl. Acad. Sci. U S A.

[bib50] Kubo M., Imai A., Nishiyama T., Ishikawa M., Sato Y., Kurata T., Hiwatashi Y., Reski R., Hasebe M. (2013). System for stable β-estradiol-inducible gene expression in the moss *Physcomitrella patens*. PLoS One.

[bib51] Lang C., Smith L.S., Long S.R. (2018). Characterization of novel plant symbiosis mutants using a new multiple gene-expression reporter *Sinorhizobium meliloti* strain. Front. Plant Sci..

[bib52] Lang D., Ullrich K.K., Murat F., Fuchs J., Jenkins J., Haas F.B., Piednoel M., Gundlach H., Van Bel M., Meyberg R. (2018). The *Physcomitrella patens* chromosome-scale assembly reveals moss genome structure and evolution. Plant J..

[bib53] Levene H., Ingram O., Hotelling H. (1960). Contributions to Probability and Statistics: Essays in Honor of Harold Hotelling.

[bib54] Levy J., Bres C., Geurts R., Chalhoub B., Kulikova O., Duc G., Journet E.P., Ané J.M., Lauber E., Bisseling T., Denarie J. (2004). A putative Ca^2+^ and calmodulin-dependent protein kinase required for bacterial and fungal symbioses. Science.

[bib55] de León I.P., Hamberg M., Castresana C. (2015). Oxylipins in moss development and defense. Front. Plant Sci..

[bib120] Liu Y., Johnson M.G., Cox C.J., Medina R., Devos N., Vanderpoorten A., Hedenäs L., Bell N.E., Shevock J.R., Aguero B., Quandt D. (2019). Resolution of the ordinal phylogeny of mosses using targeted exons from organellar and nuclear genomes. Nat. Commun..

[bib56] Ma F., Lu R., Liu H., Shi B., Zhang J., Tan M., Zhang A., Jiang M. (2012). Nitric oxide-activated calcium/calmodulin-dependent protein kinase regulates the abscisic acid-induced antioxidant defence in maize. J. Exp. Bot..

[bib57] Maillet F., Poinsot V., Andre O., Puech-Pagès V., Haouy A., Gueunier M., Cromer L., Giraudet D., Formey D., Niebel A. (2011). Fungal lipochitooligosaccharide symbiotic signals in arbuscular mycorrhiza. Nature.

[bib58] Mann D.G.J., LaFayette P.R., Abercrombie L.L., King Z.R., Mazarei M., Halter M.C., Poovaiah C.R., Baxter H., Shen H., Dixon R.A. (2012). Gateway-compatible vectors for high-throughput gene functional analysis in switchgrass (*Panicum virgatum* L.) and other monocot species. Plant Biotechnol. J..

[bib59] Messinese E., Mun J.-H., Yeun L.H., Jayaraman D., Rougé P., Barre A., Lougnon G., Schornack S., Bono J.-J., Cook D.R. (2007). A novel nuclear protein interacts with the symbiotic DMI3 calcium-and calmodulin-dependent protein kinase of *Medicago truncatula*. Mol. Plant Microbe Interact..

[bib60] Miller J.B., Pratap A., Miyahara A., Zhou L., Bornemann S., Morris R.J., Oldroyd G.E.D. (2013). Calcium/Calmodulin-dependent protein kinase is negatively and positively regulated by calcium, providing a mechanism for decoding calcium responses during symbiosis signaling. Plant Cell.

[bib61] Miyata K., Kozaki T., Kouzai Y., Ozawa K., Ishii K., Asamizu E., Okabe Y., Umehara Y., Miyamoto A., Kobae Y. (2014). Bifunctional plant receptor, OsCERK1, regulates both chitin-triggered immunity and arbuscular mycorrhizal symbiosis in rice. Plant Cell Physiol.

[bib62] Moser M., Kirkpatrick A., Groves N.R., Meier I. (2020). LINC-complex mediated positioning of the vegetative nucleus is involved in calcium and ROS signaling in Arabidopsis pollen tubes. Nucleus.

[bib63] Mun T., Bachmann A., Gupta V., Stougaard J., Andersen S.U. (2016). Lotus Base: an integrated information portal for the model legume *Lotus japonicus*. Sci. Rep..

[bib119] Newton A.E., Cox C.J., Duckett J.G., Wheeler J.A., Goffinet B., Hedderson T.A., Mishler B.D. (2000). Evolution of the major moss lineages: phylogenetic analyses based on multiple gene sequences and morphology. Bryologist.

[bib64] Okada M., Takezawa D., Tachibanaki S., Kawamura S., Tokumitsu H., Kobayashi R. (2003). Neuronal calcium sensor proteins are direct targets of the insulinotropic agent repaglinide. Biochem. J..

[bib65] Oldroyd G.E.D. (2013). Speak, friend, and enter: signalling systems that promote beneficial symbiotic associations in plants. Nat. Rev. Microbiol..

[bib66] Oliver J.P., Castro A., Gaggero C., Cascón T., Schmelz E.A., Castresana C., de León I.P. (2009). *Pythium* infection activates conserved plant defense responses in mosses. Planta.

[bib67] Ondzighi-Assoume C.A., Chakraborty S., Harris J.M. (2016). Environmental nitrate stimulates abscisic acid accumulation in arabidopsis root tips by releasing it from inactive stores. Plant Cell.

[bib68] Ortiz-Ramírez C., Hernandez-Coronado M., Thamm A., Catarino B., Wang M., Dolan L., Feijó J.A.A., Becker J.D.D. (2016). A Transcriptome atlas of *Physcomitrella patens* provides insights into the evolution and development of land plants. Mol. Plant.

[bib69] Parniske M. (2008). Arbuscular mycorrhiza: the mother of plant root endosymbioses. Nat. Rev. Microbiol..

[bib70] Pirozynski K.A., Malloch D.W. (1975). The origin of land plants: a matter of mycotrophism. Biosystems.

[bib71] Ponce de León I. (2011). The moss *Physcomitrella patens* as a model system to study interactions between plants and phytopathogenic fungi and oomycetes. J. Pathog..

[bib72] Pressel S., Duckett J.G. (2010). Cytological insights into the desiccation biology of a model system: moss protonemata. New Phytol..

[bib73] Pressel S., Bidartondo M.I., Ligrone R., Duckett J.G. (2010). Fungal symbioses in bryophytes: new insights in the twenty first century. Phytotaxa.

[bib74] R Core Team (2014). https://www.R-project.org/.

[bib75] Rabatin S.C. (1980). The occurrence of the vesicular-arbuscular-mycorrhizal fungus *Glomus tenuis* with moss. Mycologia.

[bib76] Radhakrishnan G.V., Keller J., Rich M.K., Vernié T., Mbadinga Mbadinga D.L., Vigneron N., Cottret L., Clemente H.S., Libourel C., Cheema J. (2020). An ancestral signalling pathway is conserved in intracellular symbioses-forming plant lineages. Nat. Plants.

[bib77] Read D.J., Duckett J.G., Francis R., Ligrone R., Russell A. (2000). Symbiotic fungal associations in “lower” land plants. Philos. Trans. R. Soc. Lond. B. Biol. Sci..

[bib78] Redecker D., Kodner R., Graham L.E. (2000). Glomalean fungi from the Ordovician. Science.

[bib79] Remy W., Taylor T.N., Hass H., Kerp H. (1994). Four hundred-million-year-old vesicular arbuscular mycorrhizae. Proc. Natl. Acad. Sci. U S A.

[bib80] Rensing S.A., Lang D., Zimmer A.D., Terry A., Salamov A., Shapiro H., Nishiyama T., Perroud P.-F., Lindquist E.A., Kamisugi Y. (2008). The *Physcomitrella* genome reveals evolutionary insights into the conquest of land by plants. Science.

[bib81] Rensing S.A., Goffinet B., Meyberg R., Wu S.Z., Bezanilla M. (2020). The moss *Physcomitrium* (*Physcomitrella) patens*: a model organism for non-seed plants. Plant Cell.

[bib82] Routray P., Miller J.B., Du L., Oldroyd G., Poovaiah B.W. (2013). Phosphorylation of S344 in the calmodulin-binding domain negatively affects CCaMK function during bacterial and fungal symbioses. Plant J..

[bib83] Russell A.J., Cove D.J., Trewavas A.J., Wang T.L. (1998). Blue light but not red light induces a calcium transient in the moss *Physcomitrella patens* (Hedw.) B., S. &G. Planta.

[bib84] Schiessl K., Lilley J.L.S., Lee T., Tamvakis I., Kohlen W., Bailey P.C., Thomas A., Luptak J., Ramakrishnan K., Carpenter M.D. (2019). NODULE INCEPTION recruits the lateral root developmental program for symbiotic nodule Organogenesis in *Medicago truncatula*. Curr. Biol..

[bib85] Schindelin J., Arganda-Carreras I., Frise E., Kaynig V., Longair M., Pietzsch T., Preibisch S., Rueden C., Saalfeld S., Schmid B., Tinevez J.Y. (2012). Fiji: an open-source platform for biological-image analysis. Nat. Methods.

[bib86] Schnepf E., Reinhard C. (1997). Brachycytes in *Funaria* protonemate: induction by abscisic acid and fine structure. J. Plant Physiol..

[bib87] Schulze S., Dubeaux G., Ceciliato P.H., Munemasa S., Nuhkat M., Yarmolinsky D., Aguilar J., Diaz R., Azoulay-Shemer T., Steinhorst L., Offenborn J.N. (2021). A role for calcium-dependent protein kinases in differential CO_2_-and ABA-controlled stomatal closing and low CO_2_-induced stomatal opening in Arabidopsis. New Phytol..

[bib88] Shi B., Ni L., Zhang A., Cao J., Zhang H., Qin T., Tan M., Zhang J., Jiang M. (2012). *OsDMI3* is a novel component of abscisic acid signaling in the induction of antioxidant defense in leaves of rice. Mol. Plant.

[bib89] Shi B., Ni L., Liu Y., Zhang A., Tan M., Jiang M. (2014). OsDMI3-mediated activation of OsMPK1 regulates the activities of antioxidant enzymes in abscisic acid signalling in rice. Plant Cell Environ..

[bib90] Shimomura A., Naka A., Miyazaki N., Moriuchi S., Arima S., Sato S., Hirakawa H., Hayashi M., Maymon M., Hirsch A.M., Suzuki A. (2016). Blue light perception by both roots and rhizobia inhibits nodule formation in *Lotus japonicus*. Mol. Plant Microbe Interact..

[bib91] Shinde S., Nurul Islam M., Ng C.K. (2012). Dehydration stress-induced oscillations in LEA protein transcripts involves abscisic acid in the moss, *Physcomitrella patens*. New Phytol..

[bib92] Shinde S., Shinde R., Downey F., Ng C.K. (2013). Abiotic stress-induced oscillations in steady-state transcript levels of Group 3 LEA protein genes in the moss, *Physcomitrella patens*. Plant Signal. Behav..

[bib93] Singh S., Katzer K., Lambert J., Cerri M., Parniske M. (2014). CYCLOPS, A DNA-binding transcriptional activator, orchestrates symbiotic root nodule development. Cell Host Microbe.

[bib94] Smith S.E., Read D.J. (2010).

[bib95] Spatafora J.W., Chang Y., Benny G.L., Lazarus K., Smith M.E., Berbee M.L., Bonito G., Corradi N., Grigoriev I., Gryganskyi A. (2016). A phylum-level phylogenetic classification of zygomycete fungi based on genome-scale data. Mycologia.

[bib96] Stoelzle S., Kagawa T., Wada M., Hedrich R., Dietrich P. (2003). Blue light activates calcium-permeable channels in *Arabidopsis* mesophyll cells via the phototropin signaling pathway. Proc. Natl. Acad. Sci. U S A.

[bib97] Strullu-Derrien C., Kenrick P., Pressel S., Duckett J.G., Rioult J.P., Strullu D.G. (2014). Fungal associations in *Horneophyton ligneri* from the Rhynie Chert (c. 407 million year old) closely resemble those in extant lower land plants: novel insights into ancestral plant-fungus symbioses. New Phytol..

[bib98] Strullu-Derrien C., Wawrzyniak Z., Goral T., Kenrick P. (2015). Fungal colonization of the rooting system of the early land plant *Asteroxylon mackiei* from the 407-Myr-old Rhynie Chert (Scotland, UK). Bot. J. Linn. Soc..

[bib99] Sun J., Miller J.B., Granqvist E., Wiley-Kalil A., Gobbato E., Maillet F., Cottaz S., Samain E., Venkateshwaran M., Fort S. (2015). Activation of symbiosis signaling by arbuscular mycorrhizal fungi in legumes and rice. Plant Cell.

[bib100] Takeda N., Maekawa T., Hayashi M. (2012). Nuclear-localized and deregulated calcium- and calmodulin-dependent protein kinase activates rhizobial and mycorrhizal responses in *Lotus japonicus*. Plant Cell.

[bib101] Takezawa D., Watanabe N., Ghosh T.K., Saruhashi M., Suzuki A., Ishiyama K., Somemiya S., Kobayashi M., Sakata Y. (2015). Epoxycarotenoid-mediated synthesis of abscisic acid in *Physcomitrella patens* implicating conserved mechanisms for acclimation to hyperosmosis in embryophytes. New Phytol..

[bib102] Tang H., Krishnakumar V., Bidwell S., Rosen B., Chan A., Zhou S., Gentzbittel L., Childs K.L., Yandell M., Gundlach H. (2014). An improved genome release (version Mt4.0) for the model legume *Medicago truncatula*. BMC Genomics.

[bib103] Tirichine L., Imaizumi-Anraku H., Yoshida S., Murakami Y., Madsen L.H., Miwa H., Nakagawa T., Sandal N., Albrektsen A.S., Kawaguchi M. (2006). Deregulation of a Ca^2+^/calmodulin-dependent kinase leads to spontaneous nodule development. Nature.

[bib104] Tukey J.W. (1949). Comparing individual means in the analysis of variance. Biometrics.

[bib105] Valdés-López O., Jayaraman D., Maeda J., Delaux P.M., Venkateshwaran M., Isidra-Arellano M.C., Reyero-Saavedra M.D.R., Sánchez-Correa M.D.S., Verastegui-Vidal M.A., Delgado-Buenrostro N. (2019). A novel positive regulator of the early stages of root nodule symbiosis identified by phosphoproteomics. Plant Cell Physiol.

[bib106] Venkateshwaran M., Volkening J.D., Sussman M.R., Ané J.-M. (2013). Symbiosis and the social network of higher plants. Curr. Opin. Plant Biol..

[bib107] Vesty E.F., Saidi Y., Moody L.A., Holloway D., Whitbread A., Needs S., Choudhary A., Burns B., McLeod D., Bradshaw S.J. (2016). The decision to germinate is regulated by divergent molecular networks in spores and seeds. New Phytol..

[bib108] Wang B., Qiu Y.-L.Y.L. (2006). Phylogenetic distribution and evolution of mycorrhizas in land plants. Mycorrhiza.

[bib109] Wang B., Yeun L.H., Xue J.Y., Liu Y., Ané J.-M., Qiu Y.L. (2010). Presence of three mycorrhizal genes in the common ancestor of land plants suggests a key role of mycorrhizas in the colonization of land by plants. New Phytol..

[bib110] Wang C., Liu Y., Li S.-S., Han G.-Z. (2015). Insights into the origin and evolution of the plant hormone signaling machinery. Plant Physiol..

[bib111] Webb A.A., Larman M.G., Montgomery L.T., Taylor J.E., Hetherington A.M. (2001). The role of calcium in ABA-induced gene expression and stomatal movements. Plant J..

[bib112] Xue L., Cui H., Buer B., Vijayakumar V., Delaux P.-M., Junkermann S., Bucher M. (2015). Network of GRAS transcription factors involved in the control of arbuscule development in *Lotus japonicus*. Plant Physiol..

[bib113] Yan J., Guan L., Sun Y., Zhu Y., Liu L., Lu R., Jiang M., Tan M., Zhang A. (2015). Calcium and ZmCCaMK are involved in brassinosteroid-induced antioxidant defense in maize leaves. Plant Cell Physiol..

[bib114] Yang C., Li A., Zhao Y., Zhang Z., Zhu Y., Tan X., Geng S., Guo H., Zhang X., Kang Z. (2011). Overexpression of a wheat *CCaMK* gene reduces ABA sensitivity of *Arabidopsis thaliana* during seed germination and seedling growth. Plant Mol. Biol. Rep..

[bib115] Yano K., Yoshida S., Müller J., Singh S., Banba M., Vickers K., Markmann K., White C., Schuller B., Sato S. (2008). CYCLOPS, a mediator of symbiotic intracellular accommodation. Proc. Natl. Acad. Sci. U S A.

[bib116] Yotsui I., Saruhashi M., Kawato T., Taji T., Hayashi T., Quatrano R.S., Sakata Y. (2013). ABSCISIC ACID INSENSITIVE3 regulates abscisic acid-responsive gene expression with the nuclear factor Y complex through the ACTT-core element in *Physcomitrella patens*. New Phytol..

[bib118] Zhou X.X., Chung H.K., Lam A.J., Lin M.Z. (2012). Optical control of protein activity by fluorescent protein domains. Science.

