# Supplementary material for: Stress-associated developmental reprogramming in moss protonemata by synthetic activation of the common symbiosis pathway
Source: iScience. 2022 Jan 11;25(2):103754. doi: 10.1016/j.isci.2022.103754 (PMC8819110; doi:10.1016/j.isci.2022.103754)
Supplement: Document S1. Figures S1–S10 and Tables S1 — –S3 [file mmc1.pdf]

iScience, Volume *25*

Supplemental information

Stress-associated developmental reprogramming in moss protonemata by synthetic activation of the common symbiosis pathway

Thomas J. Kleist, Anthony Bortolazzo, Zachary P. Keyser, Adele M. Perera, Thomas B. Irving, Muthusubramanian Venkateshwaran, Fatiha Atanjaoui, Ren-Jie Tang, Junko Maeda, Heather N. Cartwright, Michael L. Christianson, Peggy G. Lemaux, Sheng Luan, and Jean-Michel Ané

A

Locus ID	E-Value	Description	Reciprocal Locus ID	Reciprocal Description	Reciprocal E-Value
Pp3c21_15330V3	0	CCaMK	Medtr8g043970	CCaMK / DMI3	0
Pp3c19_20580V3	2×10^{-169}	CCaMKb	Medtr8g043970	CCaMK / DMI3	8×10^{-159}
Pp3c4_7390V3	3×10^{-71}	CDPK	Medtr5g089320	CDPK17-Like	0
Pp3c8_690V3	1×10^{-70}	CDPK	Medtr5g089320	CDPK17-Like	0
Pp3c3_37890V3	9×10^{-69}	MAPK-activated CDPK	Medtr8g099095	CDPK3-Like	0

B

Locus ID	E-Value	Description	Reciprocal Locus ID	Reciprocal Description	Reciprocal E-Value
Pp3c21_15330V3	0	CCaMK	Lj3g3v1739280	CCaMK	0
Pp3c19_20580V3	6×10^{-171}	CCaMKb	Lj3g3v1739280	CCaMK	6×10^{-176}
Pp3c8_690V3	1×10^{-69}	CDPK	Lj4g3v3002290	CDPK3-Like	0
Pp3c9_21410V3	2×10^{-69}	CDPK	Lj4g3v3002290	CDPK3-Like	0
Pp3c4_7390V3	5×10^{-69}	CDPK	Lj4g3v3002290	CDPK3-Like	0

C

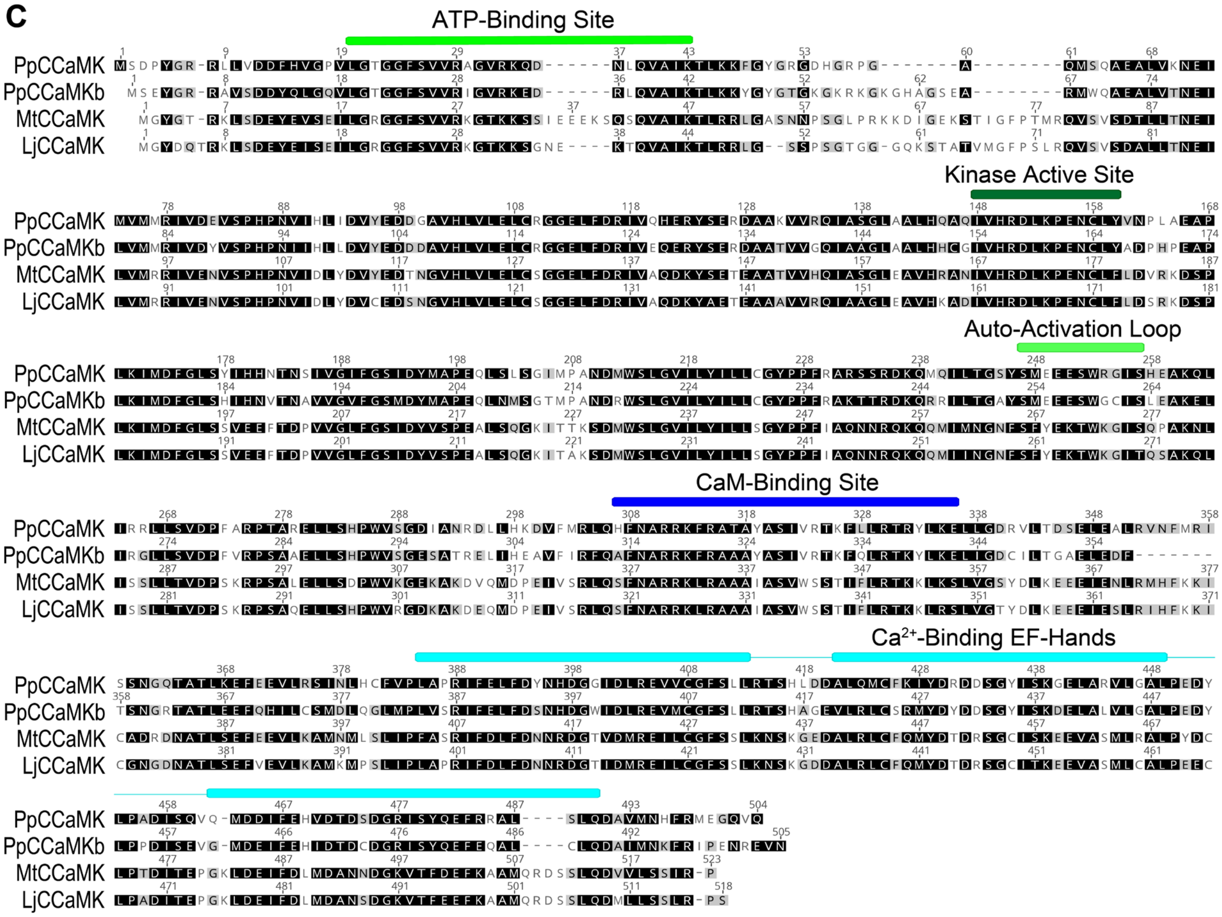

Figure S1: Bioinformatic analysis of Physcomitrium CCaMK/CCaMKb compared to characterized homologs, related to Table 1.

(**A**) Results of BLASTp search of Physcomitrium predicted proteome using Medicago CCaMK/DMI3 as query. Genomic locus ID, expectation (E)-values, and gene descriptions are provided. (Shaded) Reciprocal BLASTp with E-values for each reciprocal best hit against the Medicago predicted proteome. CDPK: Ca^{2+}-dependent protein kinase, MAPK: Mitogen-activated PK.

(**B**) Results of BLASTp search of Physcomitrium predicted proteome using Lotus CCaMK as query, and reciprocal BLAST for each against the Lotus predicted proteome. Table format is identical to (A).

(**C**) Alignment of CCaMK protein sequences made using MUSCLE. Functional predictions are annotated above the alignment. Detailed descriptions are provided in STAR Methods.

A

Locus ID	E-Value	Description	Reciprocal Locus ID	Reciprocal Description	Reciprocal E-Value
Pp3c23_22500V3	1×10^{-21}	IPD3	Medtr5g026850	IPD3	1×10^{-16}
Pp3c24_11410V3	2	Galactan 1,3-β-Galactosidase	Medtr5g015460	Glycosyl Hydrolase	8×10^{-153}
Pp3c16_24380V3	3	CCHC-Type Zinc Finger Protein	Medtr8g468600	CCHC-Type Zinc Finger Protein	2×10^{-59}
Pp3c1_2420V3	20	Xyloglucan Endotransglycosylase	Medtr4g045853	Xyloglucan Endotransglycosylase	4×10^{-55}
Pp3c25_860V3	30	Alkaline Ceramidase-Related	Medtr1g096320	Alkaline Ceramidase-Related	2×10^{-170}

B

Locus ID	E-Value	Description	Reciprocal Locus ID	Reciprocal Description	Reciprocal E-Value
Pp3c23_22500V3	1×10^{-22}	IPD3	Lj2g3v1549600	IPD3 / CYCLOPS	5×10^{-24}
Pp3c5_28790V3	4	Clathrin Assembly Protein	Lj4g3v1881980	ENTH/ANTH/VHS Family	3×10^{-6}
Pp3c24_11410V3	7	Galactan 1,3-β-Galactosidase	Lj2g3v1968430	Glycosyl Hydrolase	9×10^{-153}
Pp3c22_12090V3	10	Protein Kinase Superfamily	Lj4g3v0462390	Protein Kinase Superfamily	6×10^{-113}
Pp3c14_4840V3	10	Small Acidic Protein Family	Lj0g3v0243989	Arg/Ser-Rich Coiled Coil Protein	5×10^{-34}

C

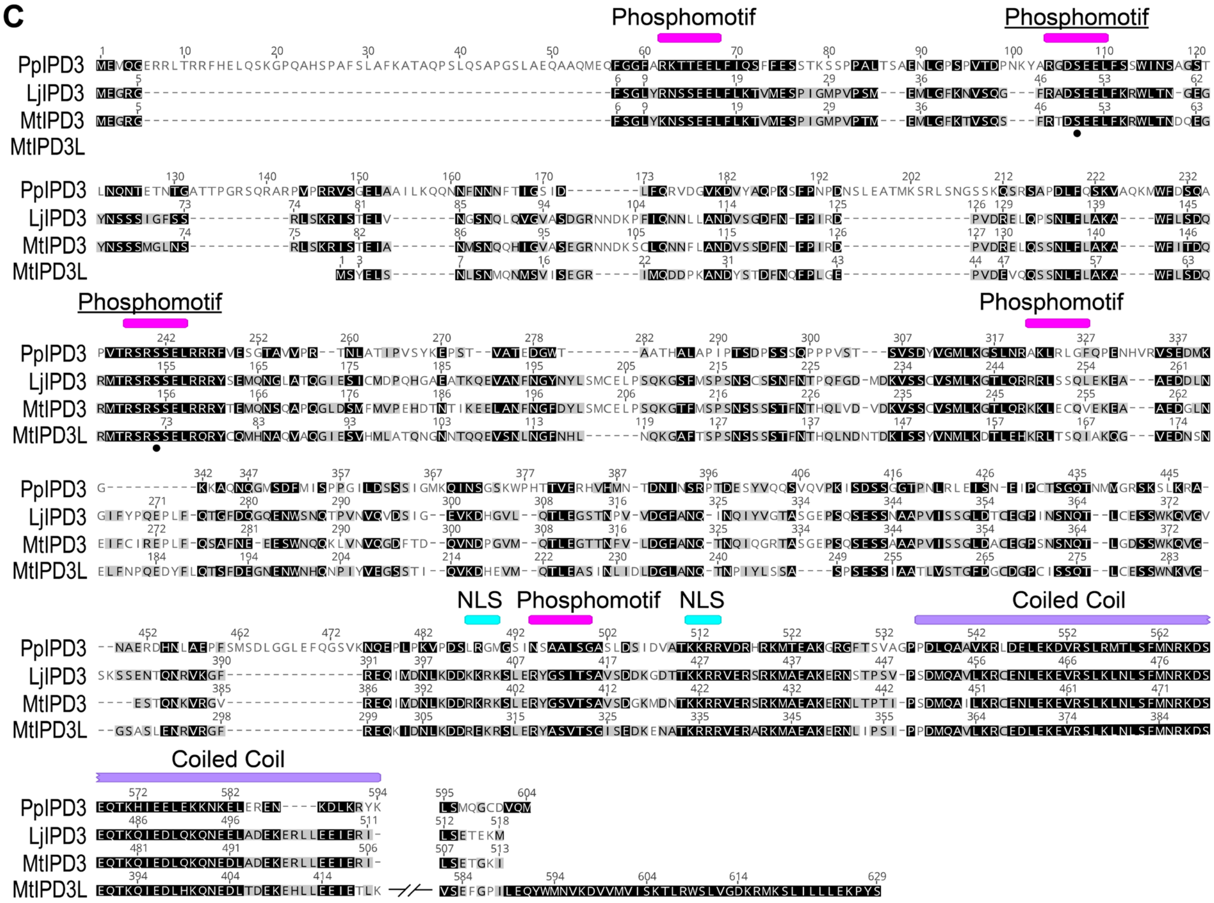

Figure S2: Bioinformatic analysis of Physcomitrium IPD3 compared to characterized homologs, related to Table 1.
(**A**) Results of BLASTp search of Physcomitrium predicted proteome using Medicago IPD3 as query. Genomic locus ID, expectation (E)-values, and gene descriptions are provided. (Shaded) Reciprocal BLASTp with E-values for each reciprocal best hit against the Medicago predicted proteome.
(**B**) Results of BLASTp search of Physcomitrium predicted proteome using Lotus IPD3/CYCLOPS as query, and reciprocal BLAST for each against the Lotus predicted proteome. Table format is identical to (A).
(**C**) Alignment of CCaMK protein sequences made using MUSCLE. Functional predictions are annotated above the alignment. Underlined phosphomotifs are critical for LjIPD3/CYCLOPS function. NLS: Nuclear localization sequence. Dashed lines indicate omitted portion of sequence for MtIPD3L.
Detailed descriptions are provided in STAR Methods.

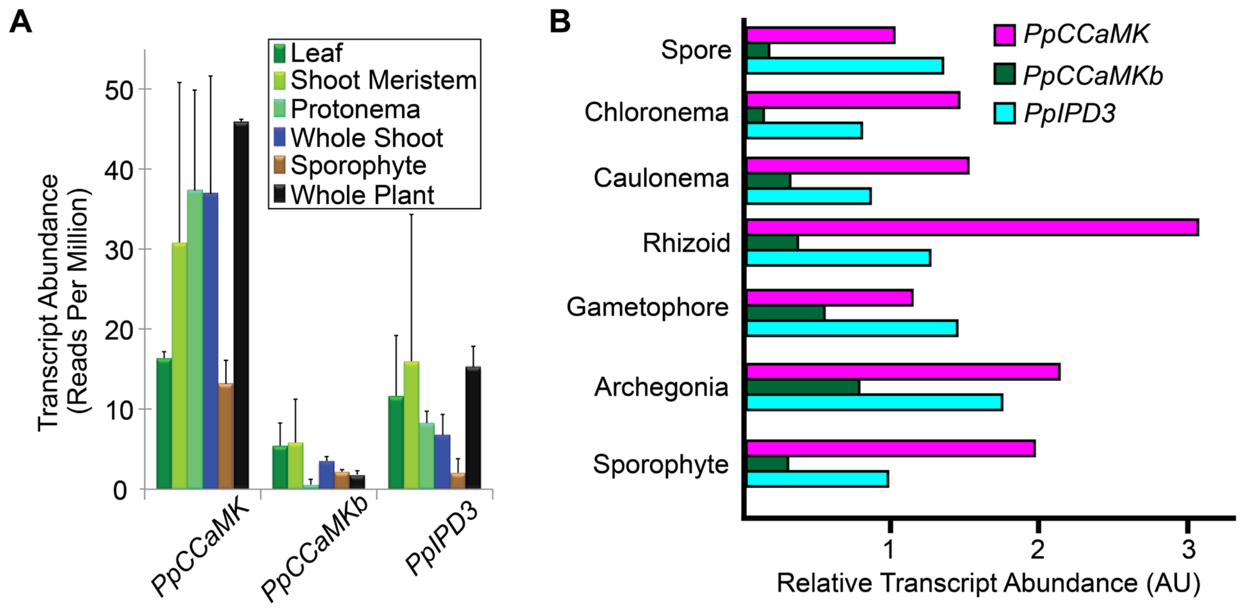

Figure S3: Transcript abundance profiles for *PpCCaMK*, *PpCCaMKb*, and *PpIPD3*. Related to Figure 1.
(**A**) Inferred transcript abundance, expressed in reads per million sequenced reads, for *PpIPD3*, *PpCCaMKb*, and *PpIPD3* across tissues sampled by laser-microdissection RNA-seq analysis (Frank and Scanlon, 2015). Error bars indicate standard error of the mean (SEM) among biological samples.
(**B**) Inferred relative transcript abundance, expressed in arbitrary units (AU) for *PpIPD3*, *PpCCaMKb*, and *PpIPD3* from a published transcriptome atlas based on RNA-seq data (Ortiz-Ramírez et al., 2016).

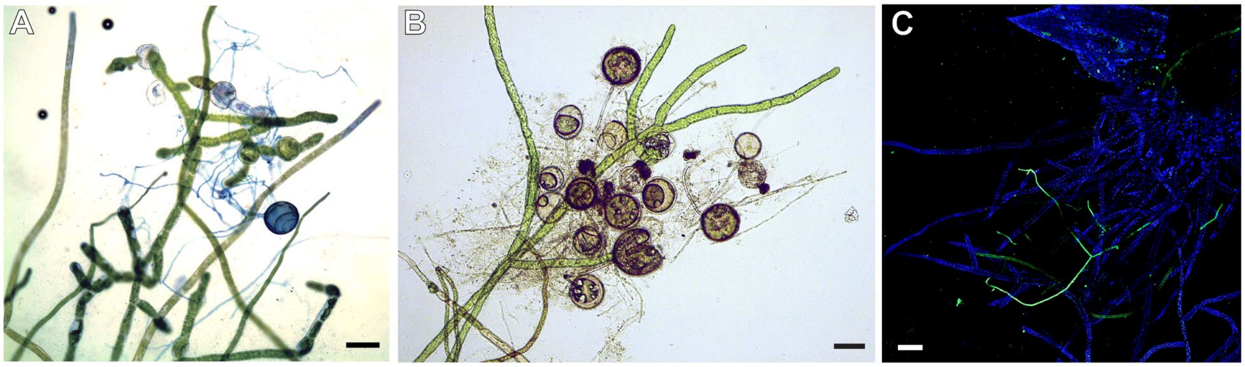

Figure S4: Co-culture of arbuscular mycorrhizal fungus *Rhizophagus irregularis* with Physcomitrium, related to Figure 1.
(**A**) Bright-field micrograph showing Physcomitrium cells from a sample that was co-cultured with *Rhizophagus irregularis* for three months. Fungal hyphae were stained with trypan blue. No evidence for intracellular colonization was observed.
(**B**) Bright-field micrograph showing Physcomitrium cells from a sample that was co-cultured with *Rhizophagus irregularis* for six months. Note that, despite extensive contact between fungal hyphae and Physcomitrium cells, no hyphal penetration was observed.
(**C**) Confocal micrograph showing Physcomitrium cells from a sample that was co-cultured with *Rhizophagus irregularis* for three months. Chlorophyll autofluorescence (blue) and fungal hyphae stained with AlexaFluor 488 (green) conjugated to wheat germ agglutinin (WGA) are shown in pseudocolor.
All scale bars = 100 µm.

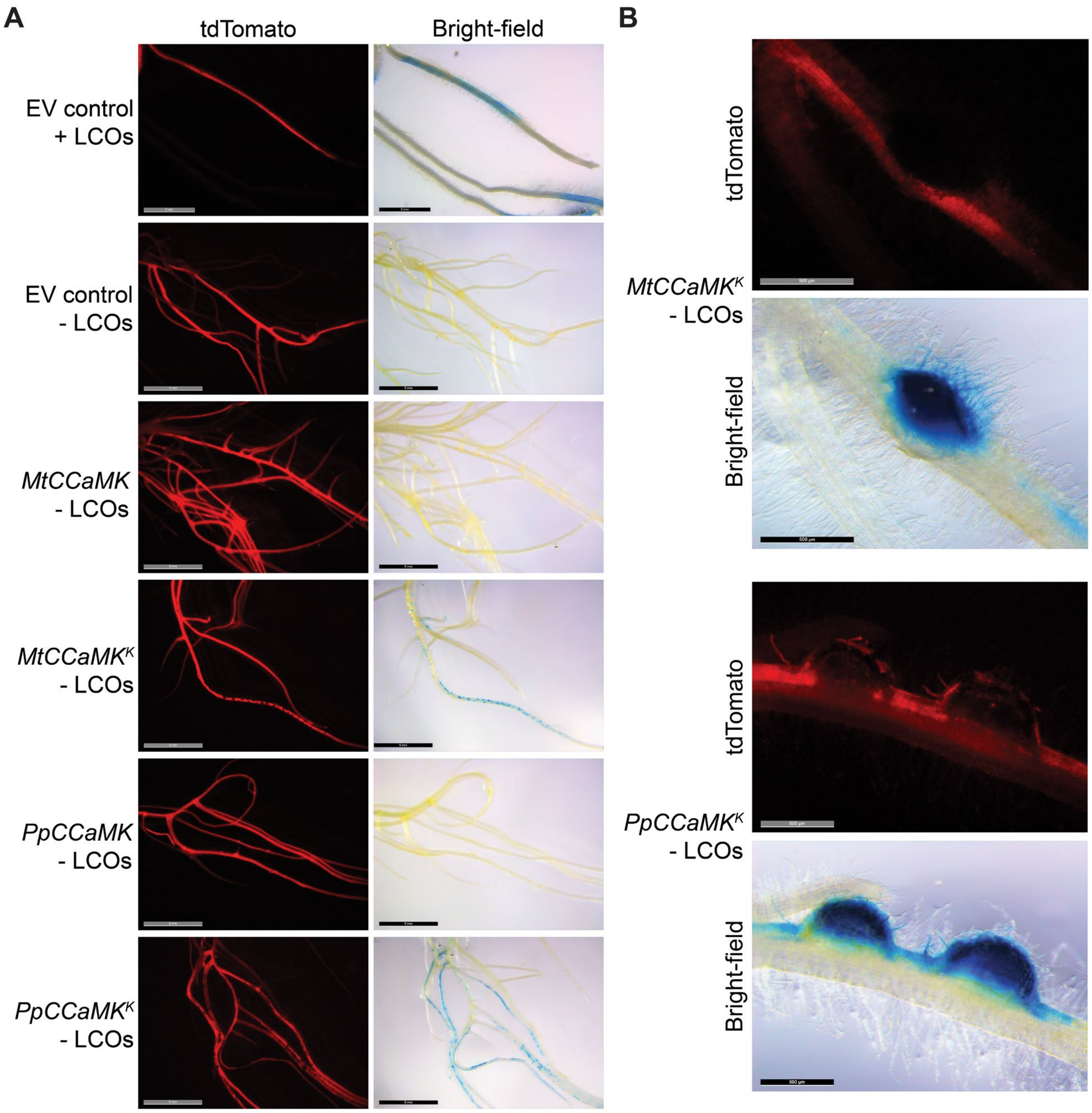

Figure S5: Deletion of the C-terminal autoinhibition domain in *PpCCaMK* results in a gain-of-function isoform (*PpCCaMK*^{K}). Related to Figure 1 and Table 1.

(**A**) Medicago roots transformed with *PpCCaMK*^{K}, or a *MtCCaMK*^{K} positive control, strongly expressed *pENOD11::GUS* without addition of LCOs or rhizobia. Scale bars for EV control plants = 2 mm; for other plants = 5 mm.

(**B**) Medicago roots expressing engineered forms of *CCaMK* from Physcomitrium (*PpCCaMK*^{K}) or Medicago (*MtCCaMK*^{K}) developed nodules in the absence of rhizobia. Scale bars = 500 µm.

The fluorescent protein tdTomato was used as a visual marker for transformation.

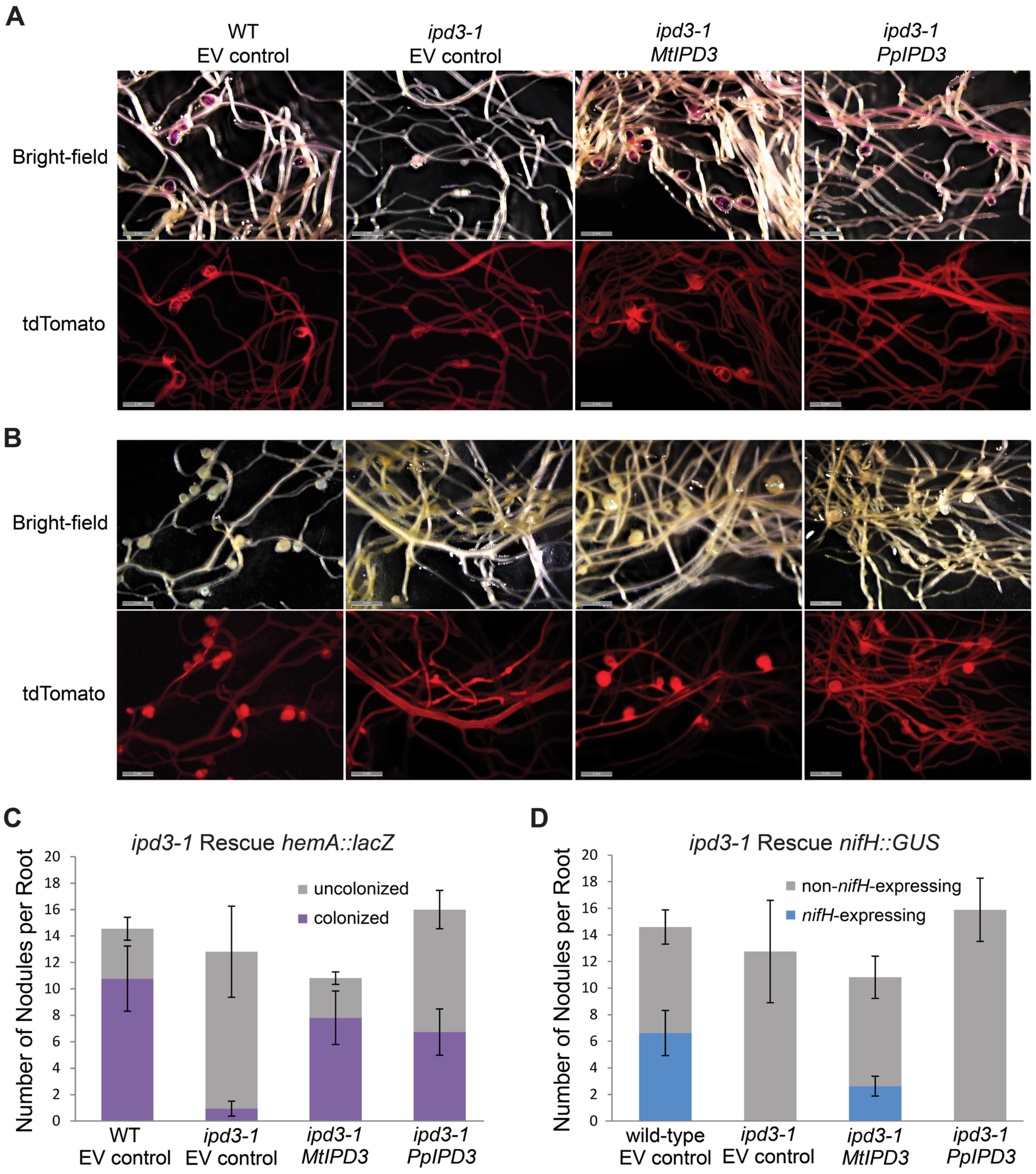

Figure S6: *PpIPD3* partially rescues nodule defects of *Medicago truncatula ipd3-1* mutants, related to Figure 1.
(**A**) Roots transformed with *PpIPD3* developed colonized nodules after inoculation with *Sinorhizobium meliloti*. EV: empty vector.
(**B**) GUS staining indicated that rhizobia inside nodules of *PpIPD3*-complemented roots did not express *nifH*, in contrast to nodules in roots complemented with *MtIPD3* (positive control). Scale bars = 2 mm.
(**C**) A statistically significant difference in the number of colonized nodules per root was calculated by Dunn's test between *ipd3-1 MtIPD3* and *ipd3-1* EV control (p-value = 2.15 x 10^{-3}) and between *ipd3-1* PpIPD3 and *ipd3-1* EV control (p-value = 1.84 x 10^{-3}).
(**D**) For number of nodules containing rhizobia expressing *nifH*, a statistically significant difference was observed using a Dunn's test between *ipd3-1* MtIPD3 and *ipd3-1* EV control (p-value = 2.73 x 10^{-3}).
Errors bars indicate standard error of mean (SEM) values (n = 16).

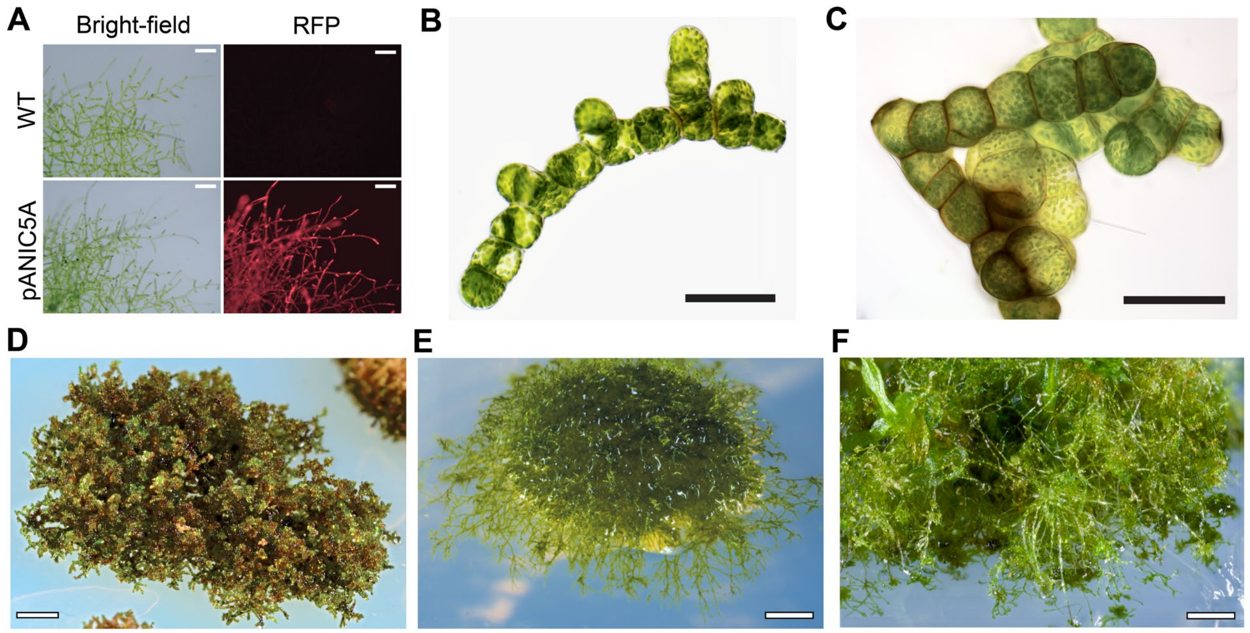

Figure S7: Growth phenotypes of PpCCaMK or PpIPD3 gain-of-function lines, related to Figure 2.
(**A**) Physcomitrium genetically transformed with the empty pANIC5A vector, which was used to drive expression of each of native or modified forms of PpCCaMK or PpIPD3 in this study, did not show any growth defects or phenotypic aberrations compared to WT. Fluorescence from a red fluorescent protein (RFP) visual marker was used to validate transformation. Scale bars = 200 µm.
(**B**) Example of juvenile cluster of cells expressing PpIPD3^{DD}. Sample was subcultured less than two weeks before imaging. Scale bar = 50 µm.
(**C**) Example of cluster of cells expressing PpIPD3^{DD} approximately three weeks after subculture. Note the promiment reddish cell wall thickenings. Scale bar = 50 µm.
(**D**) Example of Physcomitrium line expressing PpIPD3^{DD} more than 5 weeks after subculture. Note the dark pigmentation and that the entire visible population of cells are brood cells. Scale bar = 500 µm.
(**E**) Example of Physcomitrium expressing PpCCaMK^{D}. Note the abundant presence of brood cells and the absence of gametophores. Scale bar = 500 µm.
(**F**) Example of Physcomitrium expressing PpCCaMK^{D}. Note the presence of brood cells and that gametophores are stunted and malformed. Scale bar = 500 µm.
All samples were grown in BCDAT medium with 16-hour days and approximately 50 mol m^{-2} s^{-1} light intensity at 22° Celsius.

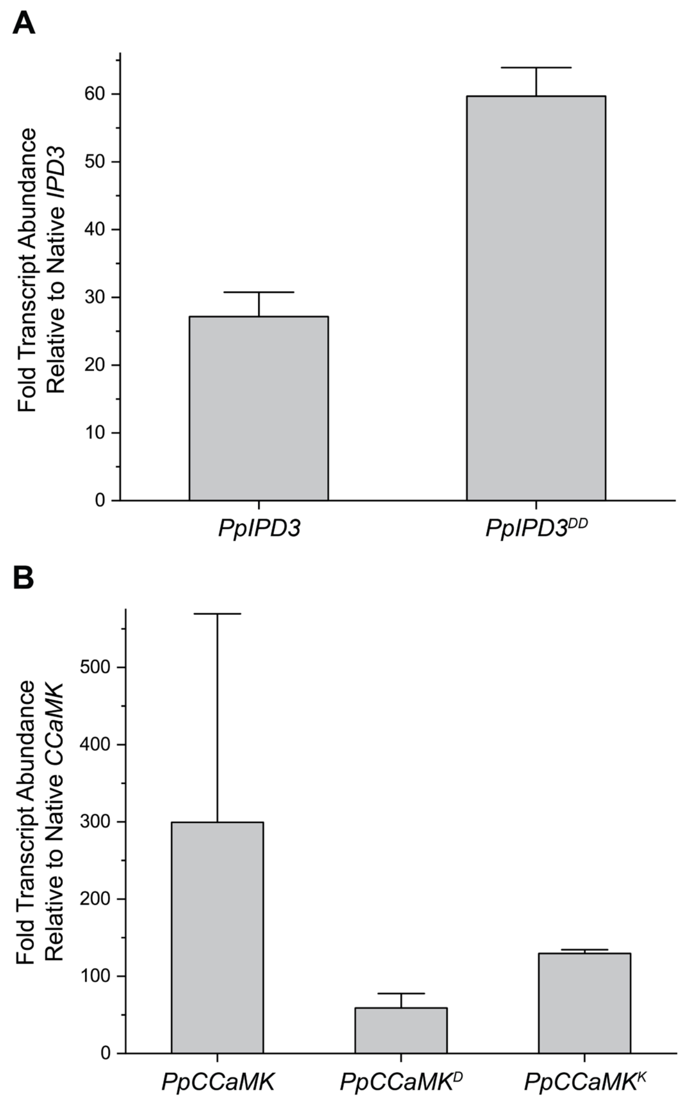

Figure S8: Quantification of transcript abundances for transgenes driven by *Zea mays UBIQUITIN1* promoter. Related to Figure 2 and Figure 3.
(**A**) Quantitative real-time PCR analysis of transcript abundances for ectopically expressed *IPD3* constructs relative to native *PpIPD3*. Mean values for two independently transformed lines (three technical replicates each) are shown. Error bars: standard error of mean (SEM).
(**B**) Quantitative real-time PCR analysis of transcript abundances for ectopically expressed *CCaMK* constructs relative to native *PpCCaMK*. Mean values for two independently transformed lines (three technical replicates each) are shown. Error bars: SEM.
Transgene nomenclature is summarized in Table 1.

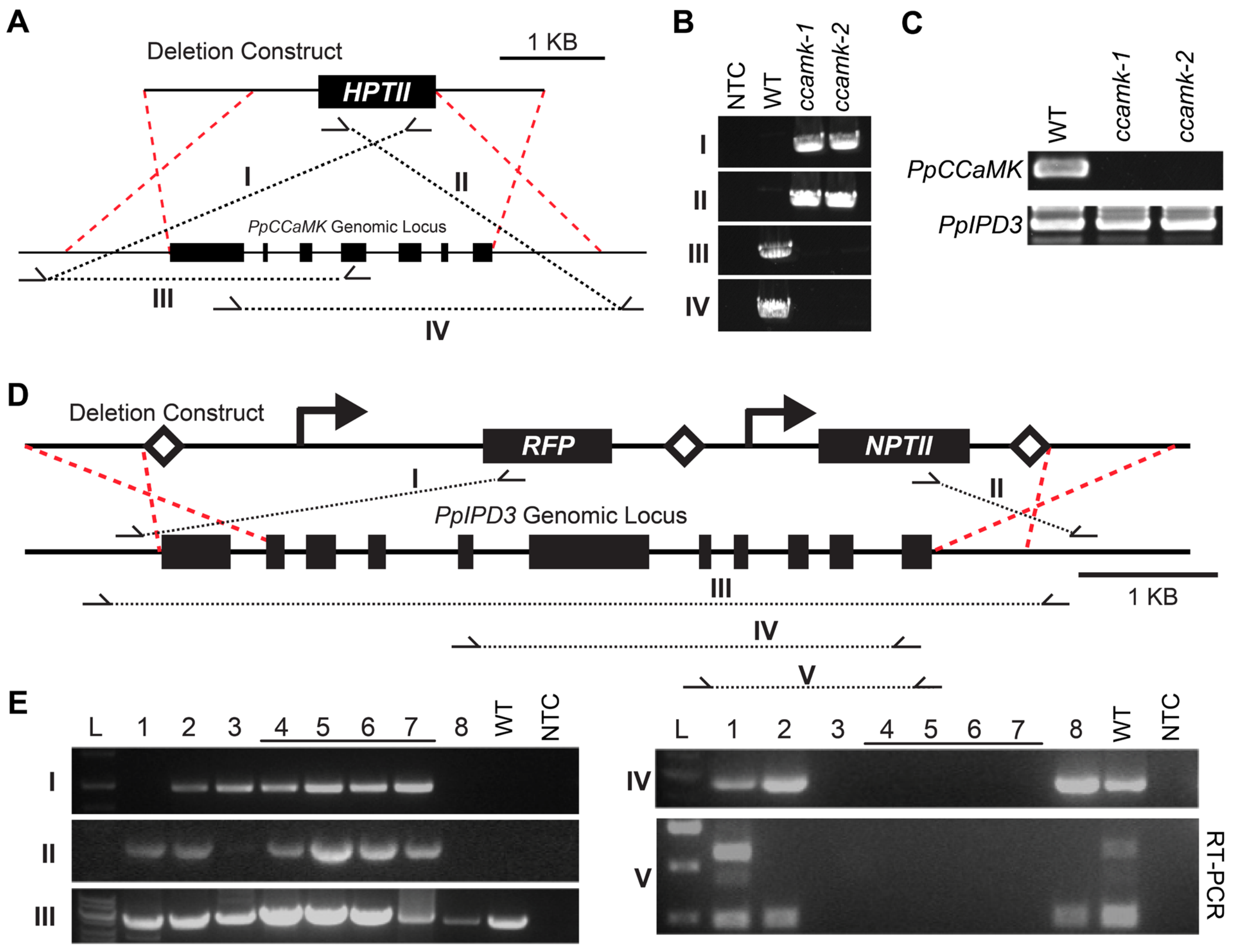

Figure S9: Genotypic characterization of transformants and identification of Physcomitrium *ccamk* and *ipd3* deletion lines generated by homologous recombination-mediated gene targeting. Related to Figure 6A-B.

(**A**) Diagram summarizing strategy for genetic deletion of *IPD3*. Roughly 1 kilobase (KB) of genomic DNA was cloned upstream or downstream of the first and last exons of *IPD3*. The two fragments were used to sandwich a hygromycin B selectable marker gene (*HPTII*) construct. Primers specific to the flanking regions of the native and recombined locus were used to test for recombination events at the targeted locus.

(**B**) Results of PCR reactions using genomic DNA extracted from two independent *ccamk* (*ccamk-1* and *ccamk-2*) deletion mutants, WT, and no template control (NTC). PCR amplicon indicated in A by roman numerals (I-IV).

(**C**) RT-PCR analysis of *ccamk-1* and *ccamk-2* using oligonucleotide primers specific to the coding sequence (CDS) of *PpCCaMK*. Amplification of *PpIPD3* from the same cDNA samples was used to validate cDNA quality.

(**D**) Diagram illustrating strategy for deletion of IPD3 and identification of knockout mutants. Deletion construct containing a red fluorescent protein (*RFP*) visible marker gene expression construct and neomycin resistance selectable marker gene (*NPTII*) expression construct is shown above the exon-intron structure of *IPD3*. Expected homologous recombination events are depicted in red dashed lines. PCR assays with five different primer pairs (I-V) were used to identify *ipd3* deletion mutants. Expected PCR products are represented by arrows and dotted lines.

(**E**) Results of PCR genotyping and RT-PCR. Panels I-IV show PCR results using genomic DNA samples. Panel V shows RT-PCR results. In each case, samples from eight lines stably transformed with *ipd3* deletion constructs (1-8) were analyzed alongside DNA ladder (L), WT sample, and NTC.

See STAR METHODS and Table S3 for further details.

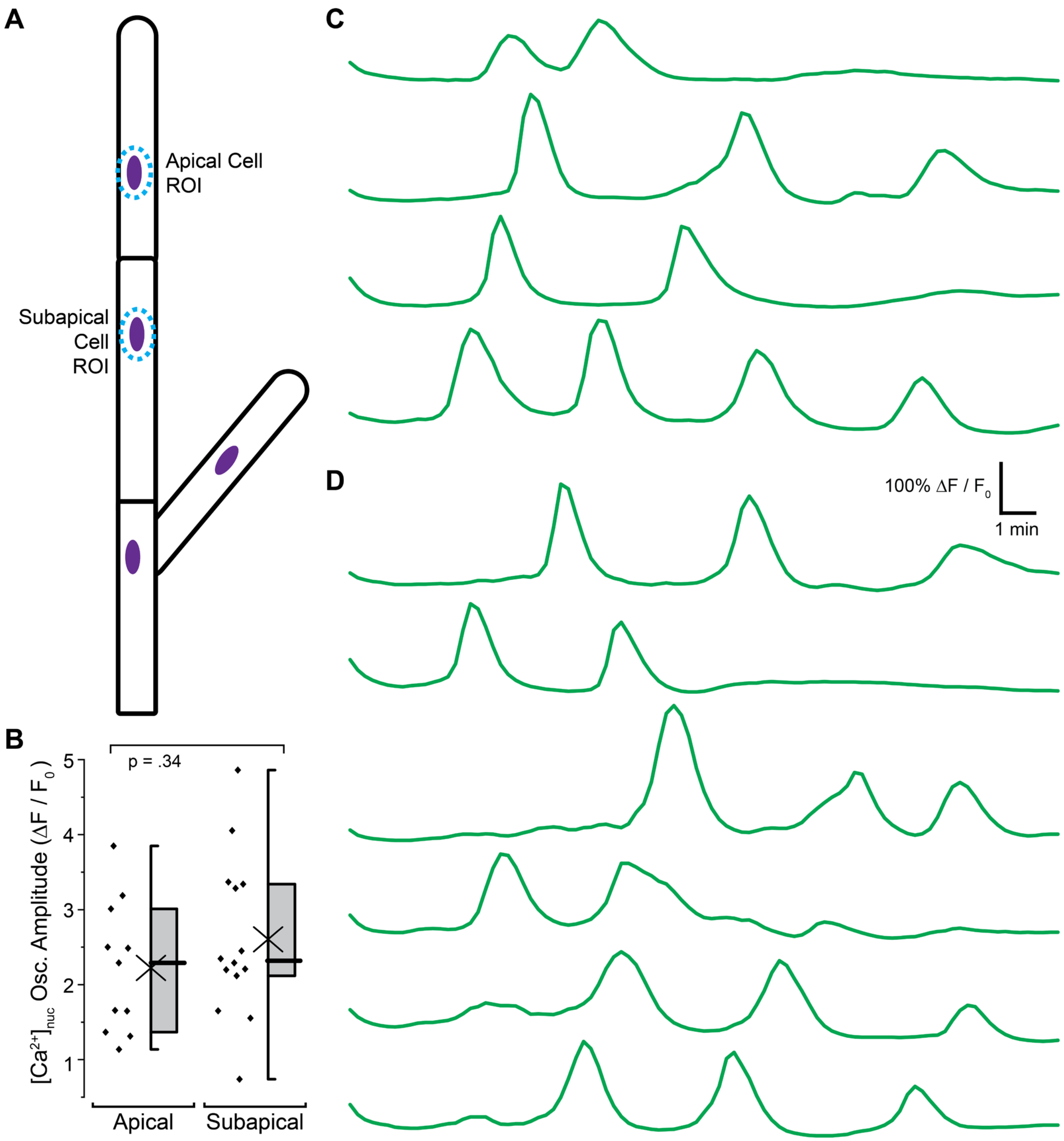

Figure S10: Spontaneous calcium spiking in protonemal nuclei, related to Figure 7.
(**A**) Schematic showing definition of apical and subapical protonemal cells and strategy for manual selection of regions of interest (ROIs, cyan dashed ellipses). Nuclei (nuc) are depicted in purple.
(**B**) Quantitative analysis of $[Ca^{2+}]_{nuc}$ oscillation (Osc.) amplitudes (ΔF / F_0) in apical and subapical cells. Statistics presented in the same format as Figure 7. Displayed p-value was calculated using Welch's t-test (n = 11 - 14). Note that data are from a separate experiment from traces shown in Figure 7C, with differences in acquisition settings accounting for magnitude discrepancies in fluorescence intensity changes.
(**C**) Examples of timecourse quantification of calcium spiking in individual apical (primary) cell nuclei.
(**D**) Examples of timecourse quantification of calcium spiking in individual subapical (secondary) cell nuclei.
Intensiometric cpGFP fluorescence of individual, manually drawn nuclear regions of interest (ROIs) is shown. Data are from an independent experiment from Figure 7C acquired using higher numerical aperture (NA) acquisition settings. Note the fluorescence intensity change (ΔF / F_0) scale (y-axis) and time scale (x-axis). See STAR Methods for further details.

	Medicago truncatula	***Lotus japonicus***	***Arabidopsis thaliana***	***Populus trichocarpa***	***Marchantia paleacea***	***Marchantia polymorpha***	***Physcomitrium patens***
DMI2	Medtr5g030920	Lj2g3v1467920	-	Potri.007G004700	1KP: LFVP_12404	-	Pp3c3_28930
DMI1	Medtr7g117580	Lj6g3v2275020<br>Lj1g3v5061360	AT5G49960	Potri.003G008800<br>Potri.004G223400	1KP: LFVP_10987	1KP: JPYU_1254	Pp3c6_21060
NUP85	Medtr1g006690	Lj1g3v0318210	AT4G32910	Potri.018G044300	1KP: IHWO_2064309	Mapoly0014s0066	Pp3c16_3750
NUP133	Medtr5g097260	Lj2g3v3337540	AT2G05120	Potri.002G221300	1KP: IHWO_2012039	Mapoly0061s0124	Pp3c21_14430
CCaMK	Medtr8g043970	Lj3g3v1739280	-	Potri.008G011400<br>Potri.010G247400	1KP: IHWO_2068101	-	Pp3c21_15330<br>Pp3c19_20580
IPD3	Medtr5g026850<br>NCBI: MG788323	Lj2g3v1549600	-	Potri.001G130800<br>Potri.003G103100	1KP: LFVP_585	-	Pp3c23_22500
NSP1	Medtr8g020840	Lj3g3v2579340.1	AT3G13840	Potri.001G168800<br>Potri.003G065400	-	1KP: JPYU_8216	Pp3c2_9730
NSP2	Medtr3g072710	Lj1g3v0785930	AT4G08250	Potri.002G086100<br>Potri.005G175300	-	-	-
RAM1	Medtr7g027190	NCBI: KU557503	-	Potri.001G326000	-	-	-
RAM2	Medtr1g040500	Lj1g3v2301880	-	Potri.006G198100<br>Potri.016G063900	1KP: LFVP_13817_3<br>1KP: LFVP_85930_3<br>1KP: LFVP_15570_2	1KP: JPYU_7323<br>1KP: JPYU_5852	Pp3c5_1510<br>Pp3c6_29200<br>Pp3c6_29290
STR	Medtr8g107450	Lj4g3v3115140	-	Potri.012G045100<br>Potri.015G036100	1KP: LFPV_2015253	-	-
STR2	Medtr5g030910	Lj0g3v0104499	-	Potri.007G004800	1KP: LFPV_2012505	-	-
VAPYRIN	Medtr6g027840	Lj0g3v0049599<br>Lj1g3v3975850	-	Potri.013G062000<br>Potri.010G185200	1KP: HMHL_4710	1KP: JPYU_2960	-

Table S1: Database accession numbers for genes referenced in Figure 1A.
Accessions from the National Center for Biotechnology Information (NCBI, accessible at https://ncbi.nlm.nih.gov) or the 1000 Plants Initiative (1KP, accessible at https://www.onekp.com) are marked. Other accessions follow standard genomic locus identified for each species. The *Lotus japonicus* genome is accessible at https://lotus.au.dk. All other data are accessible through Phytozome 12 (https://phytozome.jgi.doe.gov). Dashes indicate inferred likely genomic losses of orthologs.

A

	Mean difference	95 % CI of difference	q-Value	DF	Significance
WT vs. CCaMK	-2.692	-9.981 to 4.597	1.523	85	ns
WT vs. $CCaMK^{K}$	-10.43	-17.71 to -3.136	5.898	85	**
WT vs. $CCaMK^{D}$	-8.425	-15.71 to -1.136	4.766	85	*
WT vs. IPD3	-1.492	-8.781 to 5.797	8.439	85	ns
WT vs. $IPD3^{DD}$	-30.29	-37.58 to -23.00	17.14	85	****
CCaMK vs. $CCaMK^{K}$	-7.733	-15.14 to -0.3276	4.306	85	*
CCaMK vs. $CCaMK^{D}$	-5.733	-13.14 to 1.672	3.192	85	ns
CCaMK vs. IPD3	1.200	-6.206 to 8.606	6.682	85	ns
CCaMK vs. $IPD3^{DD}$	-27.60	-35.01 to -20.19	15.37	85	****
$CCaMK^{K}$ vs. $CCaMK^{D}$	2.000	-5.406 to 9.406	1.114	85	ns
$CCaMK^{K}$ vs. IPD3	8.933	1.528 to 16.34	4.974	85	**
$CCaMK^{K}$ vs. $IPD3^{DD}$	-19.87	-27.27 to -12.46	11.06	85	****
$CCaMK^{D}$ vs. IPD3	6.933	-0.4724 to 14.34	3.860	85	ns
$CCaMK^{D}$ vs. $IPD3^{DD}$	-21.87	-29.27 to -14.46	12.18	85	****
IPD3 vs. $IPD3^{DD}$	-28.80	-36.21 to -21.39	16.04	85	****

B

	Mean difference	95 % CI of difference	q-Value	DF	Significance
WT vs. CCaMK	-40.86	-75.76 to -5.953	4.827	85	*
WT vs. $CCaMK^{K}$	76.88	41.97 to 111.8	9.082	85	****
WT vs. $CCaMK^{D}$	31.48	-3.430 to 66.38	3.718	85	ns
WT vs. IPD3	1.942	-32.96 to 36.85	2.294	85	ns
WT vs. $IPD3^{DD}$	71.48	36.57 to 106.4	8.444	85	****
CCaMK vs. $CCaMK^{K}$	117.7	82.27 to 153.2	13.69	85	****
CCaMK vs. $CCaMK^{D}$	72.33	36.87 to 107.8	8.410	85	****
CCaMK vs. IPD3	42.80	7.336 to 78.26	4.976	85	**
CCaMK vs. $IPD3^{DD}$	112.3	76.87 to 147.8	13.06	85	****
$CCaMK^{K}$ vs. $CCaMK^{D}$	-45.40	-80.86 to -9.936	5.279	85	**
$CCaMK^{K}$ vs. IPD3	-74.93	-110.4 to -39.47	8.713	85	****
$CCaMK^{K}$ vs. $IPD3^{DD}$	-5.400	-40.86 to 30.06	6.279	85	ns
$CCaMK^{D}$ vs. IPD3	-29.53	-65.00 to 5.931	3.434	85	ns
$CCaMK^{D}$ vs. $IPD3^{DD}$	40.00	4.536 to 75.46	4.651	85	*
IPD3 vs. $IPD3^{DD}$	69.53	34.07 to 105.0	8.085	85	****

Table S2: Summary of the one-way ANOVA analysis (Tukey's multiple comparisons test) of protonemal cell dimensions of lines expressing native or modified forms of CCaMK or IPD3. Related to Figure 3.
(**A**) Comparison between measured protonemal cell widths of gain-of-function CCaMK, IPD3 in and wild-type (WT) controls.
(**B**) Comparison between measured protonemal cell lengths of gain-of-function CCaMK, IPD3 and comparison to WT controls.
Listed are the mean differences, confidence intervals (CIs), q-Values, degrees of freedom (DF), and measurements of statistical power (ns: $p > 0.05$ [non-significant]; * : $p \leq 0.05$; ** : $p \leq 0.01$; *** : $p \leq 0.001$; **** : $p \leq 0.0001$).

Primer Name	Gene	Locus ID	Purpose	Orientation	Extension Type	Primer Extension Sequence (5' to 3')	Primer Sequence (5' to 3')	Source
CCaMK_CDS-F1g	CCaMK	Pp3c21_15330	Cloning Coding Sequence	Forward	Gateway AttB1	GGGGACAAGTTTGTACAAAAAAGCAGGCTTC	ATGAGTGATCCATATGGG	This study
CCaMK_CDS-R1g	CCaMK	Pp3c21_15330	Cloning Coding Sequence	Reverse	Gateway AttB2	GGGGACCACTTTGTACAAGAAAGCTGGGTC	ATTTCACTGGACCTGACC	This study
CCaMK_CDS-R2g	CCaMK	Pp3c21_15330	Cloning Truncated Coding Sequence	Reverse	Gateway AttB2 + Stop Codon	GGGGACCACTTTGTACAAGAAAGCTGGGTCTCA	TTGTAAGCGCATGAAAACG	This study
CCaMKb_CDS-F1g	CCaMKb	Pp3c19_20580	Cloning Coding Sequence	Forward	Gateway AttB1	GGGGACAAGTTTGTACAAAAAAGCAGGCTTC	AGGATGAGTGAGTATGGG	This study
CCaMKb_CDS-R1g	CCaMKb	Pp3c19_20580	Cloning Coding Sequence	Reverse	Gateway AttB2	GGGGACCACTTTGTACAAGAAAGCTGGGTC	TCAGTTCACCTCTCTGTTC	This study
IPD3_CDS-F1g	IPD3	Pp3c23_22500	Cloning Coding Sequence	Forward	Gateway AttB1	GGGGACAAGTTTGTACAAAAAAGCAGGCTTC	ATGGAGATGCAGGGAGAGC	This study
IPD3_CDS-R1g	IPD3	Pp3c23_22500	Cloning Coding Sequence	Reverse	Gateway AttB2	GGGGACCACTTTGTACAAGAAAGCTGGGTC	TTATGACTCGGGATTAATCAGTG	This study
CCaMK_S252D-F1	CCaMK	-	Site-directed Mutagenesis	Forward	-	-	CATGGAAGAAGAAGATTGGAGAGGTATTTC	This study
CCaMK_S252D-R1	CCaMK	-	Site-directed Mutagenesis	Reverse	-	-	GAAATACCTCTCCAATCTTCTTCTTCCATG	This study
IPD3_S107D-F1	IPD3	-	Site-directed Mutagenesis	Forward	-	-	GCTAGGGGAGACGACGAGGAGCTCTTC	This study
IPD3_S107D-R1	IPD3	-	Site-directed Mutagenesis	Reverse	-	-	GAAGAGCTCCTCGTCGTCTCCCCTAGC	This study
IPD3_S241D-F1	IPD3	-	Site-directed Mutagenesis	Forward	-	-	GTAACACGCAGTCGCGATTCAGAACTGAGG	This study
IPD3_S241D-R1	IPD3	-	Site-directed Mutagenesis	Reverse	-	-	CCTCAGTTCTGAATCGCGACTGCGTGTTAC	This study
IPD3_pro_cloning_F	IPD3	Medtr5g026850	Promoter Sequence Cloning	Forward	Golden-Gate L0 PU	TGTGGTCTCAGGAG	ATAGGAACCAAAAGTAGTGATTG	This study
IPD3_pro_cloning_R	IPD3	Medtr5g026850	Promoter Sequence Cloning	Reverse	Golden-Gate L0 PU	CGTGGTCTCACATT	TTCAACACTTTAAAGATGCTTGATTA	This study
IPD3_term_cloning_F	IPD3	Medtr5g026850	Terminator Sequence Cloning	Forward	Golden-Gate L0 T	TGTGGTCTCAGCTT	TGTTTTGTTTCGCTGTTATATCCTTA	This study
IPD3_term_cloning_R	IPD3	Medtr5g026850	Terminator Sequence Cloning	Reverse	Golden-Gate L0 T	CGTGGTCTCAAGCG	TTGAATGAATAGATAAGATACC	This study
eGFP-F1g	-	-	N-terminal tagging with eGFP	Forward	Gateway AttB1	GGGGACAAGTTTGTACAAAAAAGCAGGCTTC	ATGGTGAGCAAGGGCGAG	This study
eGFP_R1+linker	-	-	N-terminal tagging with eGFP	Reverse	Poly-glycine Linker	GCCTCCACCTCCGCC	CTTGTACAGCTCGTCCATGC	This study
IPD3_CDS-F1+linker	IPD3	Pp3c23_22500	N-terminal tagging with eGFP	Forward	Poly-glycine Linker	CAAGGGCGGAGGTGGAGGC	ATGGAGATGCAGGGAGAGC	This study
IPD3_CDS-R2g	IPD3	Pp3c23_22500	N-terminal tagging with eGFP	Reverse	Gateway AttB2	GGGGACCACTTTGTACAAGAAAGCTGGGTC	TTACATCTGCACATCACATCCC	This study
Phypa_166566-F1	LEA3.1	Pp3c12_22320,30	qPCR Query	Forward	-	-	GCAGACTGGCAACTATGTGTCCGA	Shinde *et al.*, 2012
Phypa_166566-R1	LEA3.1	Pp3c12_22320,30	qPCR Query	Reverse	-	-	ATCCCACTCAACCCTACTGTTTGG	Shinde *et al.*, 2012
Phypa_166566-F2	LEA3.1	Pp3c12_22320,30	qPCR Query	Forward	-	-	GACCTATGAACAGACCAAGGAG	This study
Phypa_166566-R2	LEA3.1	Pp3c12_22320,30	qPCR Query	Reverse	-	-	GTTTTGTCACCTACTTGCTGG	This study
Phypa_211998-F1	LEA3.2	Pp3c7_13140	qPCR Query	Forward	-	-	TGCTTCAACCATGCTAGTTAGGCG	Shinde *et al.*, 2012
Phypa_211998-R1	LEA3.2	Pp3c7_13140	qPCR Query	Reverse	-	-	TCCACCTGGATCTACGGAAGTTGA	Shinde et al., 2012
Phypa_211998-F2	LEA3.2	Pp3c7_13140	qPCR Query	Forward	-	-	AAAAGAAGGATCAAGCATACGAC	This study
Phypa_211998-R2	LEA3.2	Pp3c7_13140	qPCR Query	Reverse	-	-	GTCTGTTTTGTTCCTTCCCATG	This study
Phypa_178090-F	-	Pp3c1_35630	qPCR Reference	Forward	-	-	GCAAGCCAATCAGTACACTC	LeBail *et al.*, 2013
Phypa_178090-R	-	Pp3c1_35630	qPCR Reference	Reverse	-	-	ATCTTAGCCAACAACCAATAACC	LeBail *et al.*, 2013
Phypa_430768-F	-	Pp3c14_21480	qPCR Reference	Forward	-	-	TACGGACCCTAATCCAGATGAC	LeBail *et al.*, 2013
Phypa_430768-R	-	Pp3c14_21480	qPCR Reference	Reverse	-	-	CAACCCATTGCATACTTCTGAG	LeBail *et al.*, 2013
Phypa_438496-F	-	Pp3c14_7550	qPCR Reference	Forward	-	-	ACGGACATTGCATTTAAGACCT	LeBail *et al.*, 2013
Phypa_438496-R	-	Pp3c14_7550	qPCR Reference	Reverse	-	-	GTCGATTACCTGTGGAGAAGAC	LeBail *et al.*, 2013
Phypa_443007-F	-	Pp3c8_16590	qPCR Reference	Forward	-	-	AGTATAGTCTAGAGTATGGTACCG	LeBail *et al.*, 2013
Phypa_443007-R	-	Pp3c8_16590	qPCR Reference	Reverse	-	-	TAGCAATTTGATGGCAGCTC	LeBail *et al.*, 2013
Phypa_451689-F	-	Pp3c27_6310	qPCR Reference	Forward	-	-	GTCTAGTTAGTCCTTTGGTCCT	LeBail *et al.*, 2013
Phypa_451689-R	-	Pp3c27_6310	qPCR Reference	Reverse	-	-	GCCTATTTCTATAATGACTCCGT	LeBail *et al.*, 2013
CCaMK-qF	-	Pp3c21_15330	qPCR	Forward	-	-	TATGCGGGTATCCTCCATTTCG	This study
CCaMK-qR	-	Pp3c21_15330	qPCR	Reverse	-	-	AATAACTCTCGAGCAGTGGGAC	This study
IPD3-qF	-	Pp3c23_22500	qPCR	Forward	-	-	GAGTTGGAAAAGGATGTTCGG	This study
IPD3-qR	-	Pp3c23_22500	qPCR	Reverse	-	-	CACATCACATCCCTGCATGC	This study
IPD3_GT-F1g	IPD3	Pp3c23_22500	5' Gene Targeting Flank	Forward	Gateway AttB1	GGGGACAAGTTTGTACAAAAAAGCAGGCTTA	TCGCATCTTCAGGTCCTTTCG	This study
IPD3_GT-R1g	IPD3	Pp3c23_22500	5' Gene Targeting Flank	Reverse	Gateway AttB4	GGGGACAACTTTGTATAGAAAAGTTGGGTG	TAAGGTTACGATCGGCCATCTC	This study
IPD3_GT-F2g	IPD3	Pp3c23_22500	3' Gene Targeting Flank	Forward	Gateway AttB3	GGGGACAACTTTGTATAATAAAGTTGTA	GTATCACTGATTAATCCCG	This study
IPD3_GT-R2g	IPD3	Pp3c23_22500	3' Gene Targeting Flank	Reverse	Gateway AttB2	GGGGACCACTTTGTACAAGAAAGCTGGGTA	ATGCAATATGCATAGCC	This study
RFP-F1g	-	-	Visual Marker Construct	Forward	Gateway AttB4r	GGGGACAACTTTTCTATACAAAGTTGTA	TTCAGCTGCAGGTTTGG	This study
RFP-R1g	-	-	Visual Marker Construct	Reverse	Gateway AttB5r	GGGGACAACTTTTGTATACAAAGTTGT	TCCCGATCTAGTAACATAGATG	This study
NPTII-F1g	-	-	Selectable Marker Construct	Forward	Gateway AttB5	GGGGACAACTTTGTATACAAAAGTTGTA	CTACTCCAAAAATGTCAAAG	This study
NPTII-R1g	-	-	Selectable Marker Construct	Reverse	Gateway AttB3r	GGGGACAACTTTATTATACAAAGTTGT	TGATCTGGATTTTAGTACTGG	This study
IPD3_KCA-F1	-	-	Knockout Construct Amplification	Forward	-	-	CGTTTCTTTTCTGCTCACTAGGTTTCTC	This study
IPD3_KCA-R1	-	-	Knockout Construct Amplification	Reverse	-	-	GTTTCCAAAACCGTCAGCGTTGAGC	This study
HPTII-F1	-	-	Genotyping -- 3' region (II)	Forward	-	-	GATGTGATTATCGTGAGATGTTTA	This study
HPTII-R2	-	-	Genotyping -- 5' region (I)	Reverse	-	-	TATCTGGGAACTACTCACACATTA	This study
CCaMK-CDS-R1	CCaMK	Pp3c21_15330	Genotyping -- 5' region (III)	Reverse	-	-	ACGCATGAAATTTACACGCA	This study
CCaMK-CDS-F2	CCaMK	Pp3c21_15330	Genotyping -- 3' region (IV)	Forward	-	-	GTTTGTACGTGAACCCCTTG	This study
CCaMK-gDNA-flank-F1	CCaMK	Pp3c21_15330	Genotyping--5' region (I/III)	Forward	-	-	AATAACGTGGCTTTTGGAAG	This study
CCaMK-gDNA-flank-R1	CCaMK	Pp3c21_15330	Genotyping--5' region (II/IV)	Reverse	-	-	AAAGGATCCAATCAACTCCA	This study
TB79-F	CCaMK	Pp3c21_15330	Knockout Construct Amplification	Forward	-	-	TGACCACATTCCCTTCTTCC	This study
TB79-R	CCaMK	Pp3c21_15330	Knockout Construct Amplification	Reverse	-	-	TTTCTTGTTCATATCCAAAAGACATTG	This study
IPD3_Ge-F1	IPD3	Pp3c23_22500	Genotyping -- 5' region (I)	Forward	-	-	TTCAGGTCCTTTCGTCATTTCCCTC	This study
RFP_Ge-R1	-	-	Genotyping -- 5' region (I)	Reverse	-	-	TACTCGTGCTAGTGGAAACCAAACAC	This study
NPTII_Ge-F2	-	-	Genotyping -- 3' region (II)	Forward	-	-	CATCGCCTTCTATCGCCTTC	This study
IPD3_Ge-R2	IPD3	Pp3c23_22500	Genotyping -- 3' region (II)	Reverse	-	-	ATGCATAGCCATTGTGACCATGACG	This study
IPD3_Ge-F3	IPD3	Pp3c23_22500	Genotyping -- entire locus (III)	Forward	-	-	TTGCATTCCCAATCCTCCAG	This study
IPD3_Ge-R3	IPD3	Pp3c23_22500	Genotyping -- entire locus (III)	Reverse	-	-	GTAAGGTCGGTTATTTGCTTGC	This study
IPD3_Ge-F4	IPD3	Pp3c23_22500	Genotyping -- deleted region (IV)	Forward	-	-	ATGTGGTTCGATTCACAGG	This study
IPD3_Ge-R4	IPD3	Pp3c23_22500	Genotyping -- deleted region (IV)	Reverse	-	-	CATCTGCACATCACATCCC	This study
IPD3_Ge-F5	IPD3	Pp3c23_22500	Genotyping -- RT-PCR (V)	Forward	-	-	TTAAGAACCAAGAACCCCTACC	This study
IPD3_Ge-R5	IPD3	Pp3c23_22500	Genotyping -- RT-PCR (V)	Reverse	-	-	TTCGGCATCAAAACTAAAACAG	This study

Table S3: Sequences and descriptions of oligonucleotide primers used in this study. Related to STAR Methods.
Names, descriptions, and DNA sequences (from 5' to 3') are provided. Locus IDs are for *Physcomitrium patens* genome version 3.3 or *Medicago truncatula* genome version Mt4.0. Primer pairs used in previously published studies include the citation for the source.
